# Supplementary material for: Are there ethnic and religious variations in uptake of bowel cancer screening? A retrospective cohort study among 1.7 million people in Scotland
Source: BMJ Open. 2020 Oct 7;10(10):e037011. doi: 10.1136/bmjopen-2020-037011 (PMC7542953; doi:10.1136/bmjopen-2020-037011)
Supplement: Supplementary data [file bmjopen-2020-037011supp001.pdf]

Supplementary Materials - Campbell et al Ethic and religious variations in bowel cancer screening in Scotland

Supplementary Material Figure 1

Directed Acrylic Graph (DAG) of factors influencing ethnicity and bowel screening uptake

A Directed Acrylic Graph (DAG) was developed, based on evidence form the literature, and discussion of study results. Initially 26 variables were identified as potential influencing factors to bowel screening uptake and a cross-matrix via expert opinion about the direction of influence between each of these variables was developed and tested for the causal effect identification in DAGitty v2.3 (http://dagitty.net/dags.html).

However, the initial model didn't allow estimation of the direct effect, and most of these variables were unobserved (latent) variables. The model was then stepwise reduced until a model was identified which allowed the estimation of the direct effecs. The figure shows the final model for the minimal sufficient adjustment set for estimating the direct effect of ethnicity on bowel screeming uptake, which included the variables age, country of birth, education, houshold tenure, religion, socioeconomic status (SES), and sex. This model informed the interpretation of our results and highlights areas for further investigation.

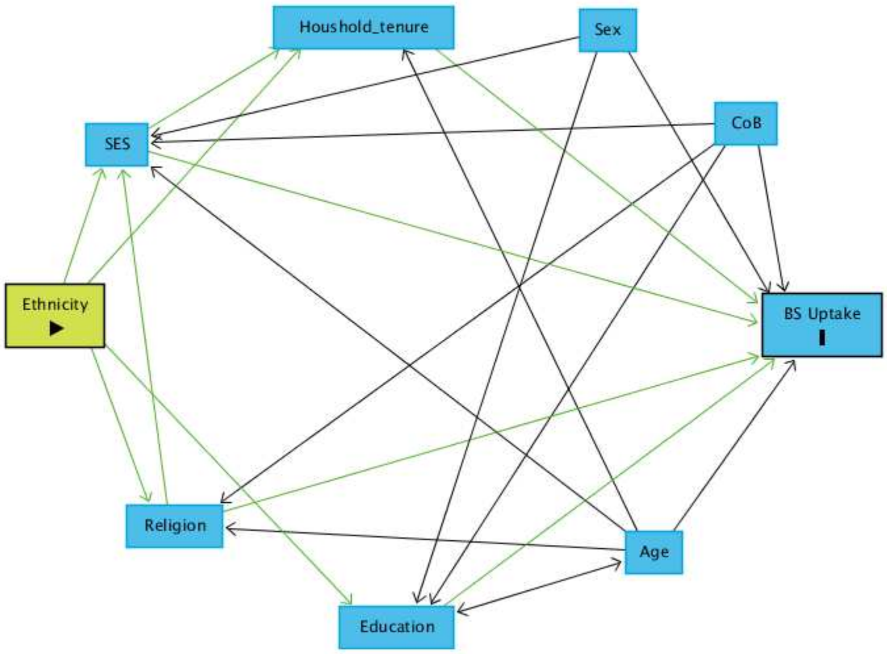

Initial factor set of 26 identified possible variables (observed variables in bold):

- **Ethnicity (Exposure)**
- **Bowel screening uptake (Outcome)**
- **Age**
- **Sex**
- Language
- Knowledge of screening
- Knowledge of cancer
- Previous experience of screening
- Health literacy
- **Education**
- Fatalism
- Perceptions of risk
- Fear of cancer
- Other priorities
- Self-efficacy
- Family history of cancer /illness
- **Country of birth (CoB)**
- **Socio-economic status**
- Marital status/ partnership
- **Religion**
- Cultural - including social norms
- Acculturation
- Frailty / co-morbidity
- **Rurality**
- Clinical support
- access to services
