# Supplementary material for: Are there ethnic and religious variations in uptake of bowel cancer screening? A retrospective cohort study among 1.7 million people in Scotland
Source: BMJ Open. 2020 Oct 7;10(10):e037011. doi: 10.1136/bmjopen-2020-037011 (PMC7542953; doi:10.1136/bmjopen-2020-037011)
Supplement: Supplementary data [file bmjopen-2020-037011supp002.pdf]

Supplementary Materials Tables for Campbell et al Are there ethnic and religious variations in uptake of bowel cancer screening in Scotland?

Supplementary Material Table 1A. Bowel cancer screening uptake (Round 1) by ethnic group in men: age adjusted rates and risk ratios (RR). RRs are adjusted for age, UK-born (versus born outside UK) and socio-economic status (household tenure and combined individual and household level education) with 95% confidence intervals (CIs).

				RRs (95% CIs): adjustment			
**Ethnic group**	**Completed screen kit returned**	**Invited into screening**	**Rates/ 100,000**	**Age**	**Age and UK/RoI-born**	**Age and 2 socio-economic variables**	**Age, UK/RoI-born and 2 socio-economic variables**
MEN							
White Scottish	362865	698715	51933	100	100	100	100
Other White British	37040	64855	57121	109.6 (108.8, 110.3)	109.5 (108.7, 110.2)	103 (102.2, 103.7)	102.9 (102.2, 103.7)
White Irish	4220	8155	51646	98.9 (96.9, 101)	98.9 (96.8, 101)	98.2 (96.2, 100.2)	98.2 (96.2, 100.2)
Other White	4045	8035	50320	98.1 (95.9, 100.2)	95.7 (93.4, 98.2)	94.7 (92.7, 96.8)	94.5 (92.2, 96.8)
Any Mixed Background	310	645	48094	94.1 (86.9, 102)	92.9 (85.7, 100.7)	94.5 (87.4, 102.3)	94.4 (87.2, 102.2)
Indian	705	1700	41495	80.5 (76.1, 85.1)	78 (73.6, 82.7)	75.4 (71.3, 79.7)	75.1 (70.9, 79.6)
Pakistani	1015	3040	33337	65.9 (62.7, 69.3)	63.7 (60.4, 67.2)	65.6 (62.4, 69)	65.4 (62, 68.9)
Bangladeshi	70	180	39013	76.6 (63.9, 91.9)	74.1 (61.8, 89)	77.9 (65.2, 93.1)	77.6 (64.9, 92.9)
Other South Asian	325	735	44492	88.6 (81.8, 96.1)	85.9 (79.1, 93.2)	86.4 (79.7, 93.7)	86.1 (79.4, 93.5)
Caribbean	90	200	45502	90.4 (77.7, 105.1)	88.5 (76.1, 102.9)	89.3 (76.7, 103.9)	89.1 (76.5, 103.7)
African	225	450	49560	99.1 (90.3, 108.7)	96.1 (87.5, 105.5)	99 (90.3, 108.6)	98.7 (89.9, 108.4)
Black Scottish or Other Black	45	110	40911	80.2 (64, 100.6)	79.2 (63.1, 99.4)	84 (67.2, 105)	83.9 (67.1, 104.9)
Chinese	990	1815	54579	107.2 (102.8, 111.8)	103.6 (99, 108.4)	107.4 (103, 112)	107 (102.3, 111.9)

240620

Supplementary Materials Tables for Campbell et al Are there ethnic and religious variations in uptake of bowel cancer screening in Scotland?

Supplementary Material Table 1B. Bowel cancer screening uptake (round 1) by ethnic group in women: age adjusted rates and risk ratios (RR). RRs are adjusted for age, UK-born (versus born outside UK) and socio-economic status (household tenure and combined individual and household level education) with 95% confidence intervals (CIs).

				RRs (95% CIs): adjustment			
**Ethnic group**	**Completed screen kit returned**	**Invited into screening**	**Rates/ 100000**	**Age**	**Age and UK/RoI-born**	**Age and 2 socio-economic variables**	**Age, UK/RoI-born and 2 socio-economic variables**
WOMEN							
White Scottish	444425	773555	57452	100	100	100	100
Other White British	42950	67160	63955	110.9 (110.2, 111.6)	110.8 (110.1, 111.4)	104.4 (103.8, 105.1)	104.4 (103.8, 105.1)
White Irish	5255	9005	58370	100.9 (99.2, 102.7)	100.9 (99.2, 102.7)	98.6 (96.9, 100.3)	98.6 (96.9, 100.3)
Other White	5840	9825	59472	103.8 (102.1, 105.5)	101 (99, 103)	97.8 (96.3, 99.4)	97.5 (95.6, 99.4)
Any Mixed Background	435	855	50564	89.2 (83.5, 95.4)	87.9 (82.3, 94)	89.2 (83.6, 95.2)	89 (83.4, 95.1)
Indian	690	1585	43409	76.1 (72, 80.5)	73.6 (69.5, 78)	72.3 (68.4, 76.4)	72 (68, 76.2)
Pakistani	870	2785	31233	55.5 (52.5, 58.6)	53.6 (50.6, 56.7)	56.7 (53.6, 59.9)	56.4 (53.3, 59.7)
Bangladeshi	40	130	32988	58.5 (45.6, 75.1)	56.7 (44.1, 72.8)	59.1 (46.2, 75.5)	58.8 (46, 75.2)
Other South Asian	225	500	45144	79.3 (72, 87.4)	77 (69.9, 84.8)	78.5 (71.5, 86.3)	78.2 (71.1, 86.1)
Caribbean	145	250	57998	102.6 (92.4, 113.9)	100.4 (90.4, 111.5)	97.3 (87.8, 107.8)	97 (87.5, 107.6)
African	190	330	57445	101.6 (92.6, 111.5)	98.4 (89.6, 108.1)	99.6 (90.8, 109.3)	99.2 (90.4, 109)
Black Scottish or Other Black	60	120	51665	90.2 (75.8, 107.2)	88.9 (74.7, 105.7)	92.7 (78.4, 109.6)	92.6 (78.3, 109.4)
Chinese	1210	1915	63190	112 (108.2, 115.9)	108 (104, 112.1)	111.7 (107.9, 115.6)	111.2 (107.1, 115.4)

240620

Supplementary Materials Tables for Campbell et al Are there ethnic and religious variations in uptake of bowel cancer screening in Scotland?

Supplementary Material Table 2A. Bowel cancer screening uptake (Round 2) by ethnic group in men: age adjusted rates and risk ratios (RRs). RRs are adjusted for age, UK/RoI-born (versus born outside UK/RoI) and socio-economic status (household tenure and combined individual and household level education) with 95% confidence intervals (CIs).

RRs (95% CIs): adjustment

Ethnic group	Completed screen kit returned	Invited into screening	Rates/ 100,000	Age	Age and UK/RoI-born	Age and 2 socio-economic variables	Age, UK/RoI-born and 2 socio-economic variables
MEN							
White Scottish	324520	590540	54952.7	100	100	100	100
Other White British	33190	55265	60056.8	108.5 (107.5, 109.6)	108.4 (107.3, 109.4)	102.4 (101.2, 103.7)	102.4 (101.2, 103.6)
White Irish	3770	6850	55073	99.7 (97.5, 101.9)	99.6 (97.5, 101.9)	99.2 (97, 101.4)	99.2 (97, 101.4)
Other White	3595	6650	54091.4	108 (91.8, 127.1)	104.3 (90.9, 119.6)	103.7 (89.5, 120.1)	102.3 (90.4, 115.8)
Any Mixed Background	265	505	52870.6	96.9 (89.2, 105.3)	95 (87.3, 103.4)	97.4 (89.7, 105.7)	96.6 (88.9, 105)
Indian	645	1440	44729	81.3 (76.8, 86.2)	77.6 (72.3, 83.3)	77.1 (72.8, 81.6)	75.7 (70.6, 81.1)
Pakistani	905	2565	35298.3	65.2 (61.7, 68.8)	62 (57.7, 66.7)	65.6 (62.1, 69.2)	64.3 (59.7, 69.2)
Bangladeshi	60	145	41666.1	76.5 (62.9, 92.9)	72.8 (59.6, 88.8)	77.1 (63.9, 93)	75.6 (62.4, 91.6)
Other South Asian	295	620	47895.8	89 (81.8, 96.8)	84.9 (77.4, 93.3)	87.1 (80.1, 94.6)	85.5 (78, 93.7)
Caribbean	75	160	46874.4	86.6 (73.5, 102.2)	83.8 (70.8, 99.1)	85.4 (72.4, 100.8)	84.3 (71.3, 99.7)
African	180	345	51872.5	96.1 (86.6, 106.6)	91.8 (82.1, 102.5)	95.7 (86.3, 106)	94 (84.2, 104.8)
Black Scottish or Other Black	45	90	46738.5	86.2 (69.3, 107.3)	84.6 (68, 105.3)	89.7 (72.3, 111.2)	89 (71.8, 110.4)
Chinese	890	1505	59162.6	108 (103.2, 113.1)	102.7 (96.3, 109.6)	109.6 (104.7, 114.8)	107.4 (100.5, 114.9)

240620

Supplementary Materials Tables for Campbell et al Are there ethnic and religious variations in uptake of bowel cancer screening in Scotland?

Supplementary Material Table 2B. Bowel cancer screening uptake (Round 2) by ethnic group in women: age adjusted rates and risk ratios (RRs). RRs are adjusted for age, UK/RoI-born (versus born outside UK/RoI) and socio-economic status (household tenure and combined individual and household level education) with 95% confidence intervals (CIs).

				RRs (95% CIs): adjustment			
**Ethnic group**	**Completed screen kit returned**	**Invited into screening**	**Rates/ 100.000**	**Age**	**Age and UK/RoI-born**	**Age and 2 socio-economic variables**	**Age, UK/RoI-born and 2 socio-economic variables**
WOMEN							
White Scottish	388730	654990	59348.8	100	100	100	100
Other White British	37600	57050	66369.9	110.7 (110, 111.4)	110.4 (109.8, 111.1)	104.5 (103.9, 105.1)	104.4 (103.8, 105.1)
White Irish	4610	7625	60891.6	101 (99.2, 102.9)	101 (99.2, 102.9)	98.9 (97.1, 100.7)	98.9 (97.1, 100.7)
Other White	5090	8215	107045.9	104.7 (102.9, 106.5)	100.2 (98.2, 102.3)	98.8 (97.2, 100.5)	97 (95, 98.9)
Any Mixed Background	390	710	55407.6	94.1 (88, 100.5)	91.8 (85.8, 98.1)	94.3 (88.3, 100.6)	93.2 (87.3, 99.5)
Indian	620	1355	45997	77.6 (73.3, 82.2)	73.5 (69.3, 78)	73.8 (69.6, 78.1)	72 (67.9, 76.4)
Pakistani	795	2330	34324.4	58.9 (55.6, 62.3)	55.6 (52.5, 59)	59.9 (56.7, 63.4)	58.5 (55.2, 62)
Bangladeshi	40	110	35432.3	60.7 (46.8, 78.7)	57.6 (44.4, 74.7)	60.4 (46.9, 77.8)	59 (45.8, 76.1)
Other South Asian	200	405	49852.6	84.2 (76.4, 92.9)	80.2 (72.7, 88.6)	83.1 (75.3, 91.6)	81.3 (73.6, 89.7)
Caribbean	120	210	57819.5	98.2 (87.5, 110.2)	94.6 (84.2, 106.1)	93.2 (83.1, 104.6)	91.7 (81.7, 102.8)
African	165	270	62375.3	105.8 (96.3, 116.2)	100.4 (91.3, 110.4)	103.1 (93.7, 113.5)	100.8 (91.5, 111.1)
Black Scottish or Other Black	45	95	49809.7	83.8 (68.6, 102.4)	81.7 (66.9, 99.8)	84.9 (70.1, 102.8)	83.9 (69.2, 101.6)
Chinese	1080	1605	67867.3	115.7 (111.8, 119.8)	109.1 (105.1, 113.3)	115.2 (111.3, 119.2)	112.2 (108.1, 116.5)

240620

Supplementary Materials Tables for Campbell et al Are there ethnic and religious variations in uptake of bowel cancer screening in Scotland?

Supplementary Material Table 3A. Bowel cancer screening uptake (Round 1) by country of birth in men: age adjusted rates and risk ratios (RRs). RRs are adjusted for age and socio-economic status (household tenure and combined individual and household level education) with 95% confidence intervals (CIs).

				RRs (95% CIs): adjustment	
**Country of birth**	**Completed screen kit returned**	**Invited into screening**	**Rates/ 100,000**	**Age**	**Age and 2 socio-economic variables**
MEN					
Scotland	355310	685975	51796.4	100	100
Northern Ireland	2720	4890	55376.5	107 (104.4, 109.7)	102.9 (100.4, 105.4)
Rep Ireland	1695	3240	52218.9	97.8 (94.6, 101.1)	99.3 (96.2, 102.6)
Other UK	42015	73785	56952.8	109.6 (108.9, 110.3)	103.2 (102.5, 103.9)
India	1070	2240	47726.8	90.1 (86.3, 94)	83.8 (80.3, 87.4)
Pakistan	855	2670	32067.9	63.8 (60.4, 67.4)	63.5 (60.1, 67.1)
Bangladesh	80	195	40936.1	81 (68.4, 96.1)	80.9 (68.4, 95.6)
China	185	345	53606.9	102.3 (92.8, 112.8)	100.1 (90.9, 110.2)
Hong Kong	815	1470	55631.6	110 (105.1, 115.2)	110 (105.1, 115.1)
Africa	1715	3330	51506.8	102.1 (98.8, 105.5)	96.2 (93.1, 99.4)
Caribbean and West Indies	130	245	52873.4	102.8 (91.5, 115.5)	96.2 (85.6, 108)
Rest of the World	5740	11135	51544.3	101.6 (99.8, 103.5)	97.7 (96, 99.5)

240620

Supplementary Materials Tables for Campbell et al Are there ethnic and religious variations in uptake of bowel cancer screening in Scotland?

Supplementary Material Table 3B. Bowel cancer screening uptake (Round 1) by country of birth in women: age adjusted rates and risk ratios (RRs). RRs are adjusted for age and socio-economic status (household tenure and combined individual and household level education) with 95% confidence intervals (CIs).

				RRs (95% CIs): adjustment	
**Country of birth**	**Completed screen kit returned**	**Invited into screening**	**Rates/ 100,000**	**Age**	**Age and 2 socio-economic variables**
WOMEN					
Scotland	435740	760005	57333.7	100	100
Northern Ireland	3410	5490	62120	108.1 (105.9, 110.4)	103.4 (101.4, 105.6)
Rep Ireland	2615	4365	59889.6	102.8 (100.3, 105.3)	101.7 (99.3, 104.1)
Other UK	47935	75540	63453.7	110.4 (109.7, 111)	104.3 (103.7, 104.9)
India	1065	2110	50385.7	86.9 (83.3, 90.6)	81.1 (77.8, 84.6)
Pakistan	780	2455	31807.7	56.6 (53.4, 60)	57.8 (54.6, 61.2)
Bangladesh	45	120	38227.6	67.8 (53.9, 85.4)	66.9 (53.3, 83.9)
China	275	435	62469.5	110.7 (103, 119.1)	108.5 (101, 116.7)
Hong Kong	850	1350	62987.5	112.6 (108, 117.3)	112.9 (108.4, 117.7)
Africa	1860	3110	59869.6	106 (103, 109.1)	99.5 (96.7, 102.4)
Caribbean and West Indies	220	335	66064.1	115.8 (107.3, 125.1)	105.7 (97.9, 114.1)
Rest of the World	8115	13755	59017.1	104.1 (102.7, 105.6)	98.9 (97.5, 100.2)

240620

Supplementary Materials Tables for Campbell et al Are there ethnic and religious variations in uptake of bowel cancer screening in Scotland?

Supplementary Material Table 4A. Bowel cancer screening uptake (Round 1) by religion of upbringing in men: age adjusted rates and risk ratios (RRs). RRs are adjusted for age, UK/RoI-born (versus born outside UK/RoI) and socio-economic status (household tenure and combined individual and household level education) with 95% confidence intervals (CIs).

				RRs (95% CIs): adjustment			
**Religion of upbringing**	**Completed screen kit returned**	**Invited into screening**	**Rates/ 100,000**	**Age**	**Age and UK/RoI-born**	**Age and 2 socio-economic variables**	**Age, UK/RoI-born and 2 socio-economic variables**
MEN							
Church of Scotland	243955	450020	54209.6	100	100	100	100
Roman Catholic	66100	134685	48802.5	91.9 (91.3, 92.4)	91.8 (91.2, 92.3)	94.6 (94, 95.1)	94.6 (94, 95.1)
Buddhist	250	495	50604.3	94.8 (86.9, 103.4)	91.9 (84.2, 100.3)	96.2 (88.2, 105)	96.1 (88, 105)
Hindu	325	730	44185.5	81.2 (74.9, 88)	78.5 (72.3, 85.2)	74.3 (68.5, 80.5)	74.2 (68.3, 80.6)
Muslim	1615	4410	36626.2	70 (67.4, 72.8)	67.7 (64.9, 70.5)	70.4 (67.7, 73.2)	70.4 (67.5, 73.3)
Sikh	310	800	38923.2	73.6 (67.5, 80.3)	71.5 (65.6, 78.1)	72.2 (66.3, 78.7)	72.2 (66.2, 78.7)
Jewish	585	1240	46939.9	86.3 (81.4, 91.6)	86.1 (81.1, 91.3)	81.6 (77, 86.6)	81.6 (77, 86.6)
Other Christian	42550	72280	58868.5	108.2 (107.5, 108.9)	108 (107.3, 108.7)	103 (102.3, 103.7)	103 (102.3, 103.7)
Other Religion	370	825	44848	85.2 (79, 91.9)	84.8 (78.6, 91.4)	84.3 (78.3, 90.9)	84.3 (78.3, 90.9)
None	38330	83260	46036.6	88.1 (87.4, 88.8)	88 (87.3, 88.7)	91.5 (90.8, 92.2)	91.5 (90.8, 92.2)

240620

Supplementary Materials Tables for Campbell et al Are there ethnic and religious variations in uptake of bowel cancer screening in Scotland?

Supplementary Material Table 4B. Bowel cancer screening uptake (Round 1) by religion of upbringing in women: age adjusted rates and risk ratios (RRs). RRs are adjusted for age, UK/RoI-born (versus born outside UK/RoI) and socio-economic status (household tenure and combined individual and household level education) with 95% confidence intervals (CIs).

				RRs (95% CIs): adjustment			
**Religion of upbringing**	**Completed screen kit returned**	**Invited into screening**	**Rates/ 100,000**	**Age**	**Age and UK/RoI-born**	**Age and 2 socio-economic variables**	**Age, UK/RoI-born and 2 socio-economic variables**
WOMEN							
Church of Scotland	300940	499325	60269	100	100	100	100
Roman Catholic	83420	156940	53154	88.9 (88.4, 89.4)	88.8 (88.3, 89.2)	91.5 (91.1, 92)	91.5 (91, 92)
Buddhist	400	710	56336.8	95.4 (89.4, 101.8)	90.1 (84.4, 96.2)	96.5 (90.5, 102.9)	95 (89, 101.4)
Hindu	280	645	43584.8	72.4 (66.3, 79)	68.3 (62.5, 74.6)	67.1 (61.5, 73.3)	66.1 (60.5, 72.2)
Muslim	1090	3265	33363.3	56.5 (53.8, 59.3)	53.3 (50.7, 56.1)	58.4 (55.6, 61.2)	57.4 (54.7, 60.4)
Sikh	340	790	43061.2	72.3 (66.7, 78.3)	68.9 (63.6, 74.7)	72.7 (67.2, 78.8)	71.8 (66.2, 77.8)
Jewish	705	1235	57014.2	93.7 (89.2, 98.3)	92.9 (88.5, 97.5)	88.4 (84.3, 92.8)	88.2 (84.1, 92.6)
Other Christian	55265	85070	64964.8	107.4 (106.9, 108)	107.1 (106.5, 107.7)	102.9 (102.4, 103.5)	102.8 (102.3, 103.4)
Other Religion	365	700	51924.7	87.2 (81.2, 93.6)	86.3 (80.4, 92.6)	88.6 (82.6, 95)	88.3 (82.4, 94.7)
None	34110	68420	49855.9	85 (84.3, 85.6)	84.8 (84.1, 85.5)	90.3 (89.6, 91)	90.2 (89.5, 90.9)

240620

Supplementary Materials Tables for Campbell et al Are there ethnic and religious variations in uptake of bowel cancer screening in Scotland?

Supplementary Material Table 5A. Positive screen test results (Round 2) by ethnic group in men: age adjusted rates and risk ratios (RRs). RRs are adjusted for age, UK/RoI-born (versus born outside UK/RoI) and socio-economic status (household tenure and combined individual and household level education) with 95% confidence intervals (CIs).

				RRs (95% CIs): adjustment			
**Ethnic group**	**Positive screen test results**	**Completed screen kit returned**	**Rates/ 100,000**	**Age**	**Age and UK/RoI-born**	**Age and 2 socio-economic variables**	**Age, UK/RoI-born and 2 socio-economic variables**
MEN							
White Scottish	10540	324520	3248.2	100	100	100	100
Other White British	705	33190	2124	65.3 (60.5, 70.4)	65.5 (60.8, 70.6)	74.3 (68.8, 80.2)	74.4 (68.9, 80.3)
White Irish	145	3770	3870.6	117.2 (100.1, 137.3)	117.3 (100.1, 137.3)	118 (100.8, 138.2)	118 (100.8, 138.2)
Other White	105	3595	2892.1	91.8 (75.9, 110.9)	101.2 (80.6, 126.9)	100 (82.7, 120.9)	104.7 (82.9, 132.1)
Any Mixed Background	10	265	4494.3	144.4 (83, 251.3)	152.4 (87, 267.1)	146.8 (84, 256.6)	150.3 (85.4, 264.6)
Indian	20	645	2790.7	86.3 (54.6, 136.4)	98.5 (60.5, 160.3)	100.1 (63.5, 157.9)	106.3 (65.4, 172.8)
Pakistani	25	905	2761.6	85.1 (57.6, 125.5)	97.8 (63.6, 150.4)	84.1 (57, 124.1)	89.6 (58.2, 138.2)
Other South Asian	10	355	3370.8	120 (68.8, 209.2)	137 (76.8, 244.3)	134.5 (77, 234.8)	142.7 (80, 254.6)
African origin	10	300	3020.1	107.3 (56.3, 204.2)	119.9 (62.1, 231.2)	113.9 (59.8, 216.9)	119.8 (62.1, 231)
Chinese	30	890	3254.7	111.8 (78.6, 159.1)	128.7 (87, 190.3)	107.6 (75.8, 152.8)	114.8 (77.6, 169.8)

240620

Supplementary Materials Tables for Campbell et al Are there ethnic and religious variations in uptake of bowel cancer screening in Scotland?

Supplementary Material Table 5B. Positive screen test results (Round 2) by ethnic group in women: age adjusted rates and risk ratios (RRs). RRs are adjusted for age, UK/RoI-born (versus born outside UK/RoI) and socio-economic status (household tenure and combined individual and household level education) with 95% confidence intervals (CIs).

RRs (95% CIs): adjustment

Ethnic group	Positive screen test results	Completed screen kit returned	Rates/ 100,000	Age	Age and UK/RoI-born	Age and 2 socio-economic variables	Age, UK/RoI-born and 2 socio-economic variables
WOMEN							
White Scottish	8050	388730	2070.9	100	100	100	100
Other White British	490	37600	1300.5	63.8 (58.3, 69.8)	64.2 (58.6, 70.2)	71.6 (65.4, 78.4)	71.8 (65.6, 78.7)
White Irish	90	4610	1973.9	91.8 (75, 112.3)	91.8 (75, 112.4)	93.9 (76.7, 114.9)	93.9 (76.7, 114.9)
Other White	70	5090	1375.5	68.3 (54.2, 86.2)	78.7 (60, 103.3)	76.8 (60.9, 96.9)	84 (63.7, 110.8)
Any Mixed Background	10	390	2561.7	132.2 (72.5, 241)	143.8 (77.9, 265.3)	134.2 (73.2, 246.1)	140.9 (76.1, 261.1)
Indian	10	620	1291.6	64.5 (32.2, 129)	77.1 (37.3, 159.3)	69.9 (35, 139.6)	78.1 (37.8, 161.3)
Pakistani	20	795	2644.8	141.7 (92.6, 216.9)	169.7 (107.5, 268)	132.7 (86.8, 202.8)	148.6 (93.8, 235.4)
Other South Asian	.	240					
African origin	10	330	2108.4	109.7 (52.6, 228.8)	127 (60.4, 267.4)	119.1 (57.3, 247.5)	130.4 (62.1, 273.6)
Chinese	20	1080	2032.6	112.6 (74.3, 170.7)	136.5 (85.8, 217.2)	108.5 (71.5, 164.7)	122.5 (76.7, 195.8)

240620

Supplementary Materials Tables for Campbell et al Are there ethnic and religious variations in uptake of bowel cancer screening in Scotland?

Supplementary Material Table 6A. Colonoscopy performed (Round 1) by ethnic group in men: age adjusted rates and risk ratios (RRs). Results exclude Grampian health board. RRs are adjusted for age, UK/RoI-born (versus born outside UK/RoI) and socio-economic status (household tenure and combined individual and household level education) with 95% confidence intervals (CIs).

				RRs (95% CIs): adjustment			
**Ethnic group**	**Colonoscopy performed**	**Positive screen test results**	**Rates/ 100,000**	**Age**	**Age and UK/RoI-born**	**Age and 2 socio-economic variables**	**Age, UK/RoI-born and 2 socio-economic variables**
MEN							
White Scottish	8380	10125	82765.4	100	100	100	100
Other White British	495	595	83172.7	100.6 (98.1, 103.1)	100.5 (98, 103)	100 (97.5, 102.5)	99.9 (97.4, 102.4)
White Irish	100	125	78144.9	94.5 (88, 101.5)	94.6 (88.1, 101.5)	94.7 (88.2, 101.7)	94.7 (88.2, 101.7)
Other White	80	95	83447.6	100.8 (95, 107)	97.6 (91.4, 104.2)	100.5 (94.7, 106.7)	97.5 (91.4, 104.1)
Any Mixed Background	.	10					
Indian	10	10	89866.7	108.9 (107.9, 109.8)	103.6 (99.7, 107.7)	108 (106.8, 109.1)	103.2 (99.2, 107.4)
Pakistani	15	20	76386.7	92.2 (76.7, 111)	88 (73, 106.2)	92 (76.3, 110.9)	88.2 (72.9, 106.5)
Other South Asian	10	10	79881.5	96.5 (76.4, 121.8)	92.8 (73.4, 117.4)	96.8 (76.3, 122.7)	93.4 (73.5, 118.7)
African origin	10	10	77028.6	93.1 (68.8, 125.9)	90.5 (67, 122.2)	93.1 (68.4, 126.6)	90.7 (66.8, 123.2)
Chinese	20	30	81307.9	98.3 (85.6, 112.9)	93.8 (81.4, 108.2)	98.1 (85.6, 112.5)	94 (81.6, 108.2)

240620

Supplementary Materials Tables for Campbell et al Are there ethnic and religious variations in uptake of bowel cancer screening in Scotland?

Supplementary Material Table 6B. Colonoscopy performed (Round 1) by ethnic group in women: age adjusted rates and risk ratios (RRs). Results exclude Grampian health board. RRs are adjusted for age, UK/RoI-born (versus born outside UK/RoI) and socio-economic status (household tenure and combined individual and household level education) with 95% confidence intervals (CIs).

				RRs (95% CIs): adjustment			
**Ethnic group**	**Colonoscopy performed**	**Positive screen test results**	**Rates/ 100,000**	**Age**	**Age and UK/RoI-born**	**Age and 2 socio-economic variables**	**Age, UK/RoI-born and 2 socio-economic variables**
WOMEN							
White Scottish	5870	7295	80466.1	100	100	100	100
Other White British	365	450	78820.6	98 (94.6, 101.4)	97.7 (94.3, 101.2)	97.4 (94.1, 100.9)	97.2 (93.9, 100.7)
White Irish	60	80	81980.5	102 (95.2, 109.2)	101.7 (95, 108.9)	102.5 (95.7, 109.7)	102.2 (95.5, 109.5)
Other White	60	85	75242.4	93.3 (84.7, 102.8)	89.7 (80.2, 100.4)	92.6 (84.1, 102)	89.3 (79.8, 99.9)
Any Mixed Background	10	10	77517.8	97.1 (74.6, 126.2)	95.6 (73.7, 124.1)	99 (76.3, 128.5)	97.6 (75.4, 126.4)
Indian	10	10	70873.4	87.9 (64.6, 119.6)	82.7 (60.3, 113.3)	86.6 (64.1, 116.9)	81.7 (60, 111.2)
Pakistani	10	15	88591.8	109.8 (108.2, 111.4)	103.3 (96.3, 110.8)	109.7 (107.3, 112.2)	103.5 (96.3, 111.3)
Other South Asian	.	.					
African origin	.	10					
Chinese	15	25	78820.6	93.3 (77.5, 112.3)	87.7 (72, 106.9)	92.3 (76.6, 111.2)	87.1 (71.4, 106.2)

240620

Supplementary Materials Tables for Campbell et al Are there ethnic and religious variations in uptake of bowel cancer screening in Scotland?

Supplementary Material Table 6C. Colonoscopy performed (Round 2) by ethnic group in men: age adjusted rates and risk ratios (RRs). Results exclude Grampian health board. RRs are adjusted for age, UK/RoI-born (versus born outside UK/RoI) and socio-economic status (household tenure and combined individual and household level education) with 95% confidence intervals (CIs).

				RRs (95% CIs): adjustment			
**Ethnic group**	**Colonoscopy performed**	**Positive screen test results**	**Rates/ 100,000**	**Age**	**Age and UK/RoI-born**	**Age and 2 socio-economic variables**	**Age, UK/RoI-born and 2 socio-economic variables**
MEN							
White Scottish	7935	9505	83482.4	100	100	100	100
Other White British	505	595	82759.7	99.3 (96.7, 101.9)	99.3 (96.7, 101.9)	98.8 (96.2, 101.5)	98.8 (96.2, 101.5)
White Irish	115	140	84825.9	101.7 (97.1, 106.5)	101.7 (97.1, 106.5)	102 (97.4, 106.9)	102 (97.4, 106.9)
Other White	85	95	84584.3	100.9 (95.5, 106.7)	101.3 (95.2, 107.7)	100.7 (95.1, 106.5)	101.4 (95.2, 108)
Any Mixed Background	10	10	90626	108.4 (107.4, 109.5)	108.6 (107.1, 110.1)	108.8 (107.2, 110.5)	109.1 (107.1, 111.1)
Indian	15	15	84961.9	101.3 (89.3, 115)	101.8 (89.3, 116.1)	101.1 (89.6, 114.1)	102.2 (90, 116)
Pakistani	20	25	78805.2	93.8 (80, 110.1)	94.3 (80.1, 110.9)	93.3 (79.5, 109.6)	94.3 (80.1, 111)
Other South Asian	10	10	90626	108.1 (107, 109.1)	108.6 (103.5, 113.9)	107.9 (105.9, 109.9)	109.1 (103.7, 114.6)
African origin	10	10	90626	107.4 (106.4, 108.5)	107.8 (103.7, 112.2)	107.8 (105.5, 110.2)	108.8 (104.1, 113.6)
Chinese	25	25	90626	108.3 (107.5, 109.2)	108.9 (103.6, 114.5)	108.6 (107.3, 109.9)	109.9 (104.4, 115.6)

240620

Supplementary Materials Tables for Campbell et al Are there ethnic and religious variations in uptake of bowel cancer screening in Scotland?

Supplementary Material Table 6D. Colonoscopy performed (Round 2) by ethnic group in women: age adjusted rates and risk ratios (RRs). Results exclude Grampian health board. RRs are adjusted for age, UK/RoI-born (versus born outside UK/RoI) and socio-economic status (household tenure and combined individual and household level education) with 95% confidence intervals (CIs).

				RRs (95% CIs): adjustment			
**Ethnic group**	**Colonoscopy performed**	**Positive screen test results**	**Rates/ 100,000**	**Age**	**Age and UK/RoI-born**	**Age and 2 socio-economic variables**	**Age, UK/RoI-born and 2 socio-economic variables**
WOMEN							
White Scottish	6080	7355	82664.9	100	100	100	100
Other White British	365	435	82495	99.8 (96.8, 102.9)	99.9 (96.9, 103)	99.4 (96.4, 102.5)	99.5 (96.5, 102.6)
White Irish	70	85	79995.2	96.9 (89.7, 104.8)	96.9 (89.6, 104.8)	96.8 (89.5, 104.7)	96.8 (89.5, 104.7)
Other White	55	65	84093.3	101.8 (95.2, 108.8)	104 (94.9, 114)	101.9 (95.3, 109)	104.3 (95.2, 114.1)
Any Mixed Background	10	10	89994.6	109.1 (107.8, 110.3)	110.1 (106.7, 113.5)	109.2 (107.9, 110.6)	110.3 (106.9, 113.8)
Indian	10	10	89994.6	107.6 (106.4, 108.8)	110.1 (102.7, 118.2)	107.1 (105.4, 108.8)	109.7 (102.2, 117.8)
Pakistani	15	20	64996.1	78.1 (58.6, 104.1)	80.6 (59.6, 108.9)	78.1 (58.7, 103.9)	80.6 (59.7, 108.9)
Other South Asian	.	.					
African origin	.	10					
Chinese	20	20	85258	102.6 (92.2, 114.1)	105.6 (92.1, 121.1)	101.8 (91.5, 113.3)	105 (91.6, 120.4)

240620

Supplementary Materials Tables for Campbell et al Are there ethnic and religious variations in uptake of bowel cancer screening in Scotland?

Supplementary Material Table 7A. Pathology detected (polyps, adenomas, cancers) (Rounds 1 & 2) by ethnic group in men: age adjusted rates and risk ratios (RRs). Results exclude Grampian health board. RRs are adjusted for age, UK/RoI-born (versus born outside UK/RoI) and socio-economic status (household tenure and combined individual and household level education) with 95% confidence intervals (CIs).

				RRs (95% CIs): adjustment			
**Ethnic group**	**Pathology detected**	**Positive screen test results**	**Rates/ 100,000**	**Age**	**Age and UK/RoI-born**	**Age and 2 socio-economic variables**	**Age, UK/RoI-born and 2 socio-economic variables**
MEN							
White Scottish	10140	19630	51655.6	100	100	100	100
Other White British	615	1190	51922	100 (94.4, 105.8)	99.9 (94.4, 105.8)	97 (91.6, 102.7)	97 (91.5, 102.7)
White Irish	140	265	52073.3	100 (89, 112.4)	100 (89.1, 112.4)	100.9 (89.9, 113.3)	100.9 (89.9, 113.3)
Other White	95	190	50291.3	98.3 (85.3, 113.3)	96.8 (82, 114.3)	96.4 (83.7, 111.1)	96.2 (81.6, 113.4)
Any Mixed Background	10	15	46756.9	90.3 (56.4, 144.6)	89.7 (55.8, 144.1)	91 (57, 145.1)	90.9 (56.9, 145.2)
Indian	15	30	48832.3	95.6 (65.8, 139.1)	93.4 (63, 138.7)	93.3 (64.2, 135.5)	93 (62.7, 138)
Pakistani	15	45	32456.4	64.5 (42.5, 97.7)	63 (40.8, 97.4)	64.2 (42.5, 97)	64.1 (41.6, 98.6)
Other South Asian	10	20	36533	73.1 (40.7, 131.3)	71.6 (39.3, 130.4)	71.2 (39.5, 128.1)	71 (38.9, 129.6)
African origin	10	15	49766.5	99.1 (60.7, 162.1)	97.4 (59, 160.9)	98.4 (60.4, 160.4)	98.2 (59.7, 161.6)
Chinese	25	55	43452	84.5 (62.5, 114.3)	82.5 (59.5, 114.4)	85.5 (63.2, 115.8)	85.3 (61.4, 118.5)

240620

BMJ Publishing Group Limited (BMJ) disclaims all liability and responsibility arising from any reliance placed on this supplemental material which has been supplied by the author(s)

Supplementary Materials Tables for Campbell et al Are there ethnic and religious variations in uptake of bowel cancer screening in Scotland?

Supplementary Material Table 7B. Pathology detected (polyps, adenomas, cancers) (Rounds 1 & 2) by ethnic group in women: age adjusted rates and risk ratios (RRs). Results exclude Grampian health board. RRs are adjusted for age, UK/RoI-born (versus born outside UK/RoI) and socio-economic status (household tenure and combined individual and household level education) with 95% confidence intervals (CIs).

				RRs (95% CIs): adjustment			
**Ethnic group**	**Pathology detected**	**Positive screen test results**	**Rates**	**Age/ 100,000**	**Age and UK/RoI-born**	**Age and 2 socio-economic variables**	**Age, UK/RoI-born and 2 socio-economic variables**
WOMEN							
White Scottish	5275	14650	36006.8	100	100	100	100
Other White British	340	880	38451.2	107 (98.2, 116.7)	107 (98.1, 116.7)	103.4 (94.8, 112.8)	103.4 (94.8, 112.9)
White Irish	70	165	40946.5	111.9 (93, 134.5)	111.8 (93, 134.5)	111.9 (93.2, 134.4)	111.9 (93.2, 134.4)
Other White	50	150	32755.6	92.2 (73.3, 115.8)	91.5 (69.9, 119.7)	89.9 (71.6, 112.9)	90.2 (69.1, 117.9)
Any Mixed Background	10	20	30072.4	85 (44, 164.2)	84.7 (43.8, 163.9)	86.4 (44, 169.9)	86.6 (44, 170.4)
Indian	10	20	41942.6	126.4 (74.2, 215.1)	125.1 (71.4, 219.2)	124 (73, 210.3)	124.6 (71.3, 217.7)
Pakistani	.	35					
Other South Asian	.	.					
African origin	.	10					
Chinese	15	45	28605.7	83.9 (52.9, 132.9)	82.9 (50.1, 137.4)	82.3 (52, 130.5)	82.8 (50.1, 137)

240620
