# Supplementary material for: Are there ethnic and religious variations in uptake of bowel cancer screening? A retrospective cohort study among 1.7 million people in Scotland
Source: BMJ Open. 2020 Oct 7;10(10):e037011. doi: 10.1136/bmjopen-2020-037011 (PMC7542953; doi:10.1136/bmjopen-2020-037011)
Supplement: Author's manuscript [file bmjopen-2020-037011.draft_revisions.pdf]

BMJ Open is committed to open peer review. As part of this commitment we make the peer review history of every article we publish publicly available.

When an article is published we post the peer reviewers' comments and the authors' responses online. We also post the versions of the paper that were used during peer review. These are the versions that the peer review comments apply to.

The versions of the paper that follow are the versions that were submitted during the peer review process. They are not the versions of record or the final published versions. They should not be cited or distributed as the published version of this manuscript.

BMJ Open is an open access journal and the full, final, typeset and author-corrected version of record of the manuscript is available on our site with no access controls, subscription charges or pay-per-view fees (http://bmjopen.bmj.com).

If you have any questions on BMJ Open's open peer review process please email info.bmjopen@bmj.com

BMJ Open

Ethnic and religious variations in uptake of bowel cancer screening among 1.7 million people in Scotland

Journal:	*BMJ Open*
Manuscript ID	bmjopen-2020-037011
Article Type:	Original research
Date Submitted by the Author:	17-Jan-2020
Complete List of Authors:	Campbell, Christine; University of Edinburgh, Usher Institute<br>Douglas, Anne; University of Edinburgh, Centre for Population Health Sciences<br>Williams, Linda; University of Edinburgh, Usher Institute<br>Cezard, Genevieve; University of St Andrews<br>Brewster, David; University of Edinburgh, Centre for Population Heath Sciences<br>Buchanan, Duncan; ISD Scotland,<br>Robb, Katie; University of Glasgow,<br>Stanners, Greg; Information Services Division<br>Weller, David; University of Edinburgh, General Practice<br>Steele, Robert; University of Dundee, Surgery and Molecular Oncology<br>Steiner, Markus; University of Aberdeen,<br>Bhopal, Raj; University of Edinburgh, Centre for Population Health Sciences
Keywords:	Gastrointestinal tumours < ONCOLOGY, PUBLIC HEALTH, Health policy < HEALTH SERVICES ADMINISTRATION & MANAGEMENT, PREVENTIVE MEDICINE

SCHOLARONE™
Manuscripts

I, the Submitting Author has the right to grant and does grant on behalf of all authors of the Work (as defined in the below author licence), an exclusive licence and/or a non-exclusive licence for contributions from authors who are: i) UK Crown employees; ii) where BMJ has agreed a CC-BY licence shall apply, and/or iii) in accordance with the terms applicable for US Federal Government officers or employees acting as part of their official duties; on a worldwide, perpetual, irrevocable, royalty-free basis to BMJ Publishing Group Ltd ("BMJ") its licensees and where the relevant Journal is co-owned by BMJ to the co-owners of the Journal, to publish the Work in this journal and any other BMJ products and to exploit all rights, as set out in our licence.

The Submitting Author accepts and understands that any supply made under these terms is made by BMJ to the Submitting Author unless you are acting as an employee on behalf of your employer or a postgraduate student of an affiliated institution which is paying any applicable article publishing charge ("APC") for Open Access articles. Where the Submitting Author wishes to make the Work available on an Open Access basis (and intends to pay the relevant APC), the terms of reuse of such Open Access shall be governed by a Creative Commons licence – details of these licences and which Creative Commons licence will apply to this Work are set out in our licence referred to above.

Other than as permitted in any relevant BMJ Author's Self Archiving Policies, I confirm this Work has not been accepted for publication elsewhere, is not being considered for publication elsewhere and does not duplicate material already published. I confirm all authors consent to publication of this Work and authorise the granting of this licence.

For peer review only

Ethnic and religious variations in uptake of bowel cancer screening among 1.7 million people in Scotland

Running title: Ethnic variation in bowel screening uptake…

Christine Campbell[1], Anne Douglas[1], Linda Williams[1], Geneviève Cezard[2], David H Brewster[3], Duncan Buchanan[4], Kathryn A. Robb[5], Greig Stanners[6], David Weller[1], Robert Steele[7], Markus Steiner[8], Raj Bhopal[1]

[1] Usher Institute, University of Edinburgh, Edinburgh, EH8 9DX, Scotland, UK

[2] Population and Health research group, School of Geography and Sustainable Development, University of St Andrews, Irvine building, St Andrews, KY16 9AL, Scotland, UK

[3] Scottish Cancer Registry, Information Services Division, Public Health & Intelligence, NHS National Services Scotland, Gyle Square, Edinburgh, EH12 9EB, Scotland, UK

[4] Information Services, NHS National Services Scotland, Gyle Square, Edinburgh, EH12 9EB, Scotland, UK

[5] Institute of Health & Wellbeing, University of Glasgow, Gartnavel Royal Hospital, 1055 Great Western Road, Glasgow G12 0XH, Scotland, UK

[6] Population Health Team, Information Services Division, NHS National Services Scotland, Meridian Court, Glasgow G2 6QE, Scotland, UK

[7] Centre for Research into Cancer Prevention and Screening (CRiPS), Medical Research Institute, Ninewells Hospital and Medical School, Dundee, DD1 9SY, Scotland, UK

[8] School of Medicine, University of Aberdeen, Aberdeen, AB25 2ZG, Scotland, UK

Corresponding Author:

Dr Christine Campbell, Usher Institute, University of Edinburgh, Teviot Place, Edinburgh, EH8 9DX, Scotland, UK; tel: +44 (0)131 650 9252; Christine.Campbell@ed.ac.uk

For peer review only
For peer review only - http://bmjopen.bmj.com/site/about/guidelines.xhtml

Key terms: colorectal screening, ethnic minority, religion, uptake

Words = 3,949

For peer review only

For peer review only - http://bmjopen.bmj.com/site/about/guidelines.xhtml

ABSTRACT

Objective

Cancer screening should be equitably accessed by all populations. Uptake of colorectal cancer screening was examined using the Scottish Health and Ethnicity Linkage Study that links the Scottish Census 2001 to health data by individual-level self-reported ethnicity and religion.

Setting

Data on 1.7 million individuals in two rounds of the Scottish Bowel Cancer Screening Programme (2007- 2013) were linked to the 2001 Census using the Scottish Community Health Index number.

Main outcome measure

Uptake of colorectal cancer screening, reported as age-adjusted rate ratios (RRs) by ethnic group and religion were calculated for men and women with 95% confidence intervals (CI).

Results

In the first, incidence screening round, compared to White Scottish men, Other White British (RR 109.6, 95% CI 108.8, 110.3) and Chinese (107.2, CI 102.8, 111.8) men had higher uptake. In contrast, men of all South Asian groups had lower uptake (Indian RR 80.5, CI 76.1, 85.1; Pakistani RR 65.9, CI 62.7, 69.3; Bangladeshi RR 76.6, CI 63.9, 91.9; Other South Asian RR 88.6, CI 81.8, 96.1). Comparable patterns were seen among women in all ethnic groups, e.g. Pakistani (RR 55.5, CI 52.5, 58.8). Variation in uptake was also observed by reli-gion, with lower rates among Hindu (RR [95%CI]: 78.4 [71.8; 85.6]), Muslim (69.5 [66.7; 72.3]), and Sikh (73.4 [67.1; 80.3]) men compared to the reference population (Church of Scotland), with similar variation among women: lower rates were also seen among those who reported being Jewish, Roman Catholic, or with no religion.

For peer review only - http://bmjopen.bmj.com/site/about/guidelines.xhtml

For peer review only

Conclusions

There are important variations in uptake of bowel cancer screening by ethnic group and religion in Scotland, for both sexes that require further research and targeted interventions.

Trial registration: n/a

Strengths and limitations of this study

- The most fine-grained analyses of bowel screening uptake by ethnicity reported to date, with a nationally tested classification of ethnic groups
- The study benefits from high overall linkage rates of census and NHS CHI numbers, with a large national population, a high linkage rate with the Bowel Screening data
- The small numbers of outcomes for some non-White populations has required aggregation of data for some ethnic groups, restricting reporting of invasive cancer for some ethnic groups due potentially disclosive numbers
- Patterns of immigration to Scotland over the last 18 years have changed, in particular among those from Eastern Europe, and we do not report on bowel screening uptake among these populations
- The reported screening uptake rates are descriptive and not explanatory: although we adjusted for determinants of ethnic inequalities in bowel screening such as SES and UK-birth but these made little difference to the patterns observed, and further potential mechanisms need to be explored

For peer review only - http://bmjopen.bmj.com/site/about/guidelines.xhtml

BACKGROUND

Bowel cancer is the third most commonly diagnosed cancer in both men and women in Scotland.[1] Bowel cancer screening using the faecal occult blood test (FOBt) was started across all NHS boards (health authorities) in Scotland between June 2007 and December 2009, with those aged between 50- 74 years and registered with a general practice invited to participate every two years.[2] Routine use of a faecal immunochemical test (FIT) was introduced in November 2017. Although progress has been made, substantial variation in uptake is still observed by deprivation,[3] however, variation by ethnicity in Scotland has not been studied.

There is growing recognition of the challenges to minimising inequalities in cancer outcomes in minority ethnic populations across the UK. Recent work has demonstrated lower awareness of the breast and cervical screening programmes compared to White survey participants and very low (less than 30% of respondents) awareness of bowel screening overall.[4] Lower attendance among Asian invitees in the UK Flexible Sigmoidoscopy Trial has also been reported.[5] The reasons for these differences are likely to include the approach of services, cultural beliefs and attitudes, and health communication and literacy barriers.[6,7]

Reporting of inequalities in uptake of cancer screening by minority ethnic group has been limited by a failure in most health systems to routinely code ethnicity accurately. As a consequence, our understanding is based on area-based measures,[8,9] self-selecting responders to surveys including items on ethnicity,[5] or name recognition software (e.g. Nam Pehchan).[10] Existing evidence is further limited by the use of very broad categories for ethnicity e.g. Indian subcontinent;[8] White/Black/Asian;[5] Hindu-Gujerati/Hindu-Other/Muslim/Sikh/Other Asian.[10] Within these constraints, variation in uptake has been observed internationally,[11] in the FOBt bowel screening pilots in England,[10,12,13] and has been reported in the English Screening Programme.[14] UK bowel screening databases (including Scotland) do not routinely include an ethnic code[15] so reported estimated uptake rates by ethnicity are based on area-

For peer review only - http://bmjopen.bmj.com/site/about/guidelines.xhtml

level characteristics rather than individual level data. Findings from other parts of the UK may not be generalisable due to differences in composition of ethnic minority groups and religious affiliations, cultural background and service provision. A better understanding of both screening uptake and screening outcomes, analysed by minority ethnic groups and by religion, will inform more targeted education and recruitment strategies.

This study made use of a unique UK resource, the Scottish Health and Ethnicity Linkage Study (SHELS): linkage of the 2001 Census in Scotland (with individual level self-reported ethnicity, country of birth, religion and a range of socio-demographic characteristics), with the Community Health Index (CHI) register number and through that to other health databases.[16,17] Linkage to the breast screening programme dataset enabled SHELS[18] to demonstrate lower uptake of breast screening among minority ethnic women in Scotland, even when adjusted for several confounding factors. This paper provides the first data from the UK where bowel screening uptake rates are examined by a full breakdown of ethnic groups including of White Scottish, Other White British, White Irish, Other White, Indian, Pakistani, Bangladeshi, Other South Asian, Caribbean, African Other Black, Chinese, in addition to self-reported religious affiliation.

For peer review only

METHODS

Data linkage

Methods of SHELS retrospective cohort studies have been published.[16,17] We followed a strict protocol that preserved anonymity and maintained separation of personal data from the Census and National Health Service (NHS), and clinical data. SHELS used computerised, probability matching of names, addresses, sex and dates of birth to link the Census 2001 for Scotland, to the CHI, which is a register of patients using the NHS. This created a file containing the linked encrypted CHI and encrypted census numbers for a cohort of 4.62 million people (95% of those completing the census and 90% of the estimated Scottish population in 2001). We used this file to link census variables to a previously linked Scottish Bowel Cancer Screening Programme (SBCSP) and Scottish Cancer Registry (SMR06) database.

Ethnicity and religious data

The Scottish Census 2001 provided ethnic group as reported by either individuals or the householder completing the form based on a question followed by a choice of 14 categories. Unless stated otherwise we have used the official categories, capitalising them as in census reports. Ethnic group is a legally required field that was well completed (95.8%) and, after imputation (4.3%), available for 100% of those completing the census form.[19] If necessary because of small numbers, we aggregated the Bangladeshi group with the Other South Asian group; and the Caribbean, African, and Black Scottish or other Black groups into one African origin group. Further grouping into a wider South Asian group was sometimes necessary because of small numbers in analysis of colonoscopy results. Following our analytical strategy, ethnic groups were only omitted to avoid potential disclosure of identity. We did not report results for the All other ethnic group as this is an exceptionally diverse group of people and it is difficult to interpret results in any meaningful way. Religion was recorded on the Scottish Census 2001 in specific categories based on both current religion and religion of upbringing.

Screening uptake

For peer review only - http://bmjopen.bmj.com/site/about/guidelines.xhtml

Individuals aged 50-74 years are invited to participate in bowel screening in Scotland every two years (a screening 'round'). Analyses were restricted by age to 50-74 years as the age range invited to participate in the screening programme, but we also examined screening uptake in the over 75s who chose to 'opt-in'.[2] Uptake of bowel screening was defined as a completed screening round using the FOBt (i.e. screenee received a positive or negative test result).

Socio-demographic data

Census data included age, sex, country of birth (UK/Republic of Ireland (RoI) born or born outside UK/RoI) and socio-economic status. Four socio-economic indicators were used: (1) the postcode-based Scottish Index of Multiple Deprivation (SIMD), (2) highest qualification of the individual, (3) a combined measure of highest qualification (individual level for people aged 16-74 and household level for children and elderly, as individual data are not collected for these groups) and (4) household tenure.

99% of the White Scottish group, 50% of the Indian group, 59% of the Pakistani group, 42% of the Other South Asian group, 41% of the African origin group, 36% of the Chinese group and 28% of the Other White group were born in the UK/RoI in our linked census database.

Outcomes

We primarily analysed uptake (persons successfully completing a kit and getting a final result i.e. an outright positive or negative result) of bowel cancer screening between 2007 and 2013 in Scotland. First and second round (i.e. where eligible participants are invited every two years) of screening were analysed separately. We further analysed the rate of positive screening test results in this participating population, and bowel cancer detection rates. The cohort of screening invitees analysed were those included in the Scottish Census 2001 who subsequently were still living in Scotland at the time of screening invitation. Round 2 figures

For peer review only - http://bmjopen.bmj.com/site/about/guidelines.xhtml

include many of the same people as in round 1 results plus some newly entering the eligible age group, and who were resident at the 2001 Census.

Data analysis

We followed a pre-specified analysis plan (https://www.ed.ac.uk/usher/scottish-health-ethnicity-linkage/key-information). We calculated, for each outcome, by sex and ethnic group: uptake in screening in both round 1 and round 2; age-adjusted rates per 100,000 population; rate ratios (RRs) and their 95% confidence intervals (CI) using Poisson regression with robust variance adjusted for age and subsequently adjusted for socio-economic status and country of birth. We multiplied the estimates by 100 to facilitate the interpretation of the results as percentages, as per the SHELS policy and analysis plan. We adopted a previously published our approach for choosing variables that were potential confounding showing consistency across ethnic groups.[16] Two socioeconomic status (SES) indicators (household tenure and combined qualification) were consistently associated with the outcome across ethnic groups. The standard reference population was the White Scottish population. We also compared uptake rates by religion, and by country of birth, separately for men and women.

Data were analysed using SAS V.9.4 (SAS Institute Inc, Cary, North Carolina, USA).

Limited availability of Grampian data

For technical reasons, data on colonoscopy, pathology (polyps, adenoma, cancer) and invasive cancer were unavailable from Grampian Health Board.

Ethics and disclosure

The work was approved by the Multicentre Research Ethics Committee for Scotland and the Privacy Advisory Committee of NHS National Services Scotland. Caldicott Guardian approval was obtained for access to the SBCSP data. The ethical and other permissions and related

For peer review only - http://bmjopen.bmj.com/site/about/guidelines.xhtml

issues have been reported in detail including an independent assessment of SHELS' approach by an ethicist.[16,17,20] To comply with the Data Protection Act and safe-setting rules the data set only contained specific disease outcomes. Other outcomes were excluded to minimise risks of inadvertent disclosure of identity. The analysis was conducted on a standalone computer in a locked room in the National Records of Scotland (NRS), by named researchers with appropriate clearance and training (LW, GC, and MS) and following a strict disclosure protocol.

Outputs leaving the safe setting as well as this manuscript were reviewed by the NRS Disclosure Committee. The analysis was done on exact numbers. However, the released numerators and denominators were rounded to the nearest 5.

Authors developed a Directed Acyclic Graph (DAG) to aid the interpretation of results and help generate areas for further investigation (Supplementary Materials Figure 1).

Patient and Public Involvement

SHELS established a Public Engagement Panel, comprised of a mix of ethnic groups, sexes and ages. This Public Engagement Panel provided PPI perspectives on SHELS methodological approach, including the research questions and design of this study. At the end of the study, results were shared with the Panel who commented on the findings and contributed to the dissemination plan.

For peer review only - http://bmjopen.bmj.com/site/about/guidelines.xhtml

RESULTS

Linkage

Linked data were available for 1,666,575 of 1,926,060 individuals invited to participate in round 1 screening, a linkage rate of 86.5%. Of the 1.67 million matched at round 1, 1,407,835 individuals were invited to round 2.

Uptake of bowel screening by ethnic group and country of birth

Uptake in specific ethnic groups were compared to the White Scottish population, unless specified otherwise. Figure 1 shows bowel cancer screening uptake in men and women for round 1 by ethnic group. For men, age-adjusted RRs were higher in the Other White British (RR [95%CI]: 109.6 [108.8; 110.3]) and Chinese (107.2 [102.8; 111.8]) groups as they were more likely to return their kit once invited to screening compared to White Scottish men. Uptake was comparatively lower in other ethnic groups and especially so in Indian (80.5 [76.1; 85.1]), Pakistani (65.9 [62.7, 69.3]), Bangladeshi (76.6 [63.9; 91.9]) and Other South Asian (88.6 [81.8; 96.1]) men. Further adjustment for UK/RoI-birth and socio-economic status did not greatly alter the associations apart from adjustment for UK/RoI-birth in Chinese men making their uptake converge towards the levels of uptake of White Scottish men (Supplementary Materials Table 1A).

Similarly in women (Figure 1), age-adjusted RRs were higher in both Other White British (110.9 [110.2; 111.6]) and Chinese (112 [108.2; 115.9) women compared to White Scottish women, and again uptake was comparatively lower in women from Indian (76.1 [72; 80.5]), Pakistani (55.5 [52.5; 58.8]), Bangladeshi 58.5 [45.6; 75.1]) and Other South Asian (79.3 [72; 107.2]) ethnic groups. Further adjustment for UK-birth and socio-economic status did not alter the associations observed (Supplementary Materials Table 1B).

For peer review only - http://bmjopen.bmj.com/site/about/guidelines.xhtml

Screening uptake rates by ethnic group for round 2 for men (Supplementary Materials Table 2A) and women (Supplementary Materials Table 2B) showed similar patterns.

Country of birth analyses for both men (Supplementary Materials Table 3A) and women (Supplementary Materials Table 3B) were consistent with the findings by ethnic group. In particular, uptake rates for men and women born in India, Pakistan or Bangladesh were lower than for those born in Scotland, while uptake rates were higher for men born in Hong Kong and for women born in either Hong Kong or China. Further adjustment for socio-economic status did not alter the associations observed.

Uptake of bowel screening by current religion and religion of upbringing

Figure 2 shows bowel cancer screening uptake in men and women for round 1 by self-reported current religion as in the 2001 Census. Age-adjusted RRs were lower among Hindu (RR [95%CI]: 78.4 [71.8; 85.6]), Muslim (69.5 [66.7; 72.3]0, and Sikh (73.4 [67.1; 80.3]) men compared to those who identified current religion as Church of Scotland. Smaller differences compared to the reference population were observed among those who reported being Jewish (87.3 [81.8, 93.2]), Roman Catholic (91.4 [90.8, 92]), or None (no religion) (97.7 [97.2, 98.2]). Further adjustment for UK/RoI-birth and socio-economic status did not alter the trends observed.

In women, there was generally a lower uptake across groups compared to those who identified current religion as Church of Scotland apart from the Other Christian, with age-adjusted RRs being lower among Hindu (73.2 [67; 80]), Muslim (57.8 [55.2; 60.5]), and Sikh (73.2 [67.4; 79.5]) women compared to the reference Church of Scotland population. Age-adjusted RRs for Roman Catholic women were lower (87.9 [87.4; 88.4]) compared to the reference Church of Scotland populations; further adjustment for UK-birth and socio-economic status only modestly reduced the differences.

For peer review only - http://bmjopen.bmj.com/site/about/guidelines.xhtml

Screening uptake rates by religion of upbringing for men (Supplementary Materials Table 4A) and women (Supplementary Materials Table 4B) showed overall similar patterns.

Positivity of bowel screening test by ethnic group

Table 1A shows that age-adjusted RRs for positivity of FOBt were lower in Other White British (60.5 [56.1; 65.3]) men compared to White Scottish men in round 1. For women, positivity by ethnic group in round 1 (Table 1B) also showed lower positivity for the other White British group (67.3 [61.6; 73.5]) compared to White Scottish women. However for selected other ethnic groups including Indian and Pakistani women there is some indication of lower test positivity rates compared to White Scottish women but CIs were wide due to the small sample size and straddling the reference value of 100. In round 2 age-adjusted RRs for FOBt positivity were lower in Other White British men compared to White Scottish men (Supplementary Materials Table 5A). Lower age-adjusted RRs were seen for Indian and Pakistani men compared with White Scottish men, although with wide confidence intervals. For women in round 2, no clear patterns were observed apart from for Other White British women who had lower test positivity compared to White Scottish women (Supplementary Materials Table 5B).

Bowel cancer detection, colonoscopy and pathology by ethnic group

Table 2 shows bowel cancer detection rates via the screening test by ethnic group, for rounds 1 and 2, and for men and women combined, as this was necessary given the small numbers. Compared to the White Scottish population, Other White British individuals had a lower age-adjusted relative risk of a diagnosis of screen-detected invasive cancer (84 [71; 99.3]); this result was not greatly altered after adjustment for UK-born and socio-economic status. Over the two rounds of screening, the number of invasive cancers found in individuals from other ethnic groups were too small to report for the risk of disclosure.

Supplementary Materials Tables 6A- 6D show that the age-adjusted relative risk of completion of screening colonoscopy following a positive FOBt did not differ for either men or women by

For peer review only - http://bmjopen.bmj.com/site/about/guidelines.xhtml

ethnic group in screening rounds 1 and 2 compared to the White Scottish population, with the exception of slightly lower RRs for Pakistani men and Indian women. The CIs for all other populations overlapped with that of the White Scottish population, for both men and women.

Supplementary Materials Tables 7A and 7B show age-adjusted rates and relative risks for pathology detected, for polyps, adenomas and cancer combined, for men and women respectively. In comparison to the White Scottish population, numbers were small in each of the other ethnic groups. Only for Pakistani men was a lower rate of pathology detected (64.5 [42.5; 97.7]) compared to White Scottish men.

Uptake of bowel screening in older individuals

Individuals aged 75 and older are able to opt-in to bowel screening in Scotland. Table 3A and 3B show age-adjusted relative risks for screening uptake by ethnic group for men and women respectively. Chinese men had higher uptake (112.8 [113.3; 114.3]) compared to White Scottish men (Table 3A), as did Chinese women compared to White Scottish women (Table 3B: 116.7 [115; 118.5]). Adjustment for SES did not greatly affect this association in either Chinese men or women, however further adjustment for UK-birth in Chinese men the relative risk converged towards that of White Scottish men.

For peer review only

DISCUSSION

Summary of findings

Although ethnic variation in colorectal screening uptake is increasingly recognised internationally,[21] detailed description in relation to specific ethnic groups is lacking. We report complex patterns of variation in colorectal cancer screening uptake by ethnic group in Scotland, with pronounced lower screening uptake among the South Asian groups compared to the White Scottish population, and higher uptake among the Chinese and Other White British populations. Differences in breast screening uptake by ethnic group in Scotland have been reported previously by our group,[18] as has variation in relation to numerous other health outcomes.[22-25]

Strengths and Limitations of the study

Our results are to our knowledge the most fine-grained analyses of bowel screening uptake by ethnicity reported to date, and with a nationally tested classification of ethnic groups. For the first time, national Scottish Census 2001 data were used to show differences in uptake for separate Indian, Pakistani and Bangladeshi groups, for separate White groups, and for the first time showing uptake among Caribbean, African, and Chinese groups as well as by religious groups. Additionally, SHELS benefits from high overall linkage rates of census and NHS CHI numbers (95%), with a large national population (4.62 million people), and in this study a high linkage rate with the Bowel Screening data (86%). However, we acknowledge that the small numbers of outcomes for some non-White populations has required aggregation of data for some ethnic groups; e.g. for invasive cancers we were unable to report on some ethnic groups due to reporting restrictions on potentially disclosive numbers (Table 3).

We are reporting on 2001 Census data. Immigration to Scotland over the last 18 years has affected the distribution of ethnic groups within Scotland:[26,27] in particular, we do not report on bowel screening uptake among the Polish population, now one of Scotland's largest ethnic groups, where breast screening uptake is low.[28] Such analyses are not possible routinely and

For peer review only - http://bmjopen.bmj.com/site/about/guidelines.xhtml

require a new study with linkage of bowel screening data to the 2011 Census. Nonetheless, the results reported here provide important insights into recent uptake patterns and set a benchmark for any future variation in bowel screening uptake rates as the population profile changes.

Finally, we recognise that the reported screening uptake rates are descriptive and not explanatory. We adjusted for determinants of ethnic inequalities in bowel screening such as SES and UK-birth but these made little difference to the patterns observed. Further potential mechanisms need to be explored, including cultural and religious beliefs, and the influence (if any) of knowledge of or exposure to screening programmes in other health systems.

Data on a number of variables (colonoscopy, pathology (polyps, adenoma, cancer) and invasive cancers) were unavailable from Grampian Health Board: sensitivity analyses (available on request) indicates that approximately 10-12% of the denominator in the Scottish population were missing for these variables in the Scottish population. Data on uptake rates however were complete.

Existing literature

We found lower rates of screening uptake in South Asian populations, reflected in both ethnic group and current religion Lower screening uptake among South Asian communities in the UK has been a feature of the screening programme since its inception[8,9,12] and the factors influencing this are increasingly being understood. Many factors such as lack of awareness and understanding of the purpose of screening, and fear and fatalism about cancer are seen across all ethnic groups.[6,29-32] However, limitations with English-language screening materials (translated materials often require request), the need to rely on younger family members, cultural difficulties associated with handling of faeces, and social norms are additional barriers among South Asian ethnic groups.[7,32]

For peer review only - http://bmjopen.bmj.com/site/about/guidelines.xhtml
For peer review only

Previous SHELS work has published lower directly age-standardised rates and ratios of colorectal cancer in the South Asian population in Scotland (especially in Pakistani men), as well as in Chinese men.[22] The RRs we report here suggesting lower RRs of FOBt positivity in Indian and Pakistani men (Tables 2A and 2B) are consistent with this, although need to be interpreted with care due to wide confidence intervals. Lower colorectal cancer rates in some ethnic communities may result in less perceived personal relevance and hence tailored educational interventions will need to acknowledge lower colorectal cancer rates while also addressing the identified barriers and facilitators.[33-36] There is a need for open discussion within bowel screening programmes and policy making of potentially variable benefits for different ethnic groups of screening uptake. The lower uptake rates *may* be appropriate for some groups, and genuine informed consent may require acknowledgement that some have less to gain in terms of absolute risk reduction. At a programmatic level, there is a balance between lower cancer risk and uptake of screening, and further work is warranted to address how issues of equality of access, cost-effectiveness, and effectiveness are maintained. Although at a population level the risk may be lower, messages aimed at the individual level need to communicate clearly the potential advantages of screening uptake within an informed choice framework.

The relatively high uptake rates among both Chinese men and women compared to the White Scottish men and women were unexpected, and not previously recognised in the Scottish population. Bansal and colleagues found that age-adjusted RRs for breast screening uptake were similar among Chinese women compared to White Scottish women.[18] High FOBt positivity rates were observed in both Chinese men and women in both rounds 1 and 2; this is despite the lack of evidence of higher incidence of colorectal cancer in the Chinese community in earlier SHELS work.[22] Further research is warranted, not only to determine if these findings can be replicated in other Chinese communities in the UK, but also to explore any cultural or other factors underlying high screening uptake. Low awareness of colorectal cancer screening was found among Chinese participants in an EthniBus survey.[4] Importantly,

For peer review only - http://bmjopen.bmj.com/site/about/guidelines.xhtml

though, as noted above low rates of colorectal screening uptake (by flexible sigmoidoscopy) have been reported in areas of high non-White ethnicity but these were not broken down by ethnic group.[37]

While numbers were relatively small, only limited variation in colorectal screening uptake was seen in the over 75 population; there is, however, some indication that South Asian men and women were less likely to opt-in.This is a self-selecting group of individuals who are likely to differ from their peers in terms of other health behaviours, motivation and levels of co-morbidity. The low overall number of opt-ins is consistent with findings from the Bowel Screening Pilot in England.[8]

Implications for policy and practice

Addressing observed inequalities in screening uptake will require multi-faceted interventions. Telephone-based interventions have been shown to increase colorectal screening uptake in ethnically-diverse areas of London[38] but have resource implications. Patient navigators have been shown to be effective in some settings.[39] Further exploratory work and engagement with local communities is needed to develop, refine and test interventions with salience to different ethnic groups. Our reported variations in uptake by religion are, seemingly, novel: in particular, the lower uptake among Roman Catholic populations compared to the reference population, persisting even when adjusted for socio-economic variables, is puzzling. Addressing such variation by religion may be amenable to targeted faith-based interventions.[40] Others have found variable influence of religiosity on screening uptake, with social support only partially mediating the relationship between religiosity and bowel screening uptake.[41] Comparing facilitators and barriers across groups may provide fresh insight into potential interventions.[41,42] Further, the introduction of the faecal immunochemical test (FIT) in the Scottish Bowel Screening Programme in late 2017 has been shown to increase overall screening uptake:[2] this provides an impetus to monitor the impact within ethnic groups over time (work currently underway by authors).

For peer review only - http://bmjopen.bmj.com/site/about/guidelines.xhtml

ADDITIONAL INFORMATION

Ethics approval and consent to participate

The work was approved by the Multicentre Research Ethics Committee for Scotland (16/12/2013, Scotland A REC Ref: 13/SS/0225) and the Privacy Advisory Committee of NHS National Services Scotland**.** The study was performed in accordance with the Declaration of Helsinki.

Availability of data and material

Researchers who wish to access SHELS data should apply to National Records of Scotland (https://www.nrscotland.gov.uk/) and ISD (http://www.isdscotland.org/). They are maintained in a secure environment and governed by ethical and other restrictions on access.

Conflict of interest

The authors declare no conflict of interest

Funding

This work was supported by the Chief Scientist's Office (grant number CZH/4/878), Cancer Research UK (grant number C3743/A16594), and supplementary funding from NHS Health Scotland. ISD and National Records of Scotland both made 'in-house' contributions to the work.

Authors' contributions

All authors meet authorship criteria. RB was the PI of SHELS, and CC was the PI of this specific component of the research. RB, CC, AD, DHB and DW conceived the study and planned it along with KAR, GS, and RS. LW, GC and MS carried out data analysis. All authors contributed to the writing of the paper. CC is the guarantor.

For peer review only

REFERENCES

1. Information Statistics Division (ISD) Scotland Cancer Statistics http://www.isdscotland.org/Health-Topics/Cancer/Cancer-Statistics/Colorectal/ *Accessed 23/08/19*

2. National Services Division NHS Scotland Bowel Cancer Screening Programme http://www.nsd.scot.nhs.uk/services/screening/bowelscreening/index.html *Accessed 23/08/19*

3. Quyn AJ, Fraser CG, Stanners G, Carey FA, Carden C, Shaukat A, et al. Uptake trends in the Scottish Bowel Screening Programme and the influences of age, sex, and deprivation. J Med Screen. 2018 Mar;25(1):24-31. doi: 10.1177/0969141317694065. Epub 2017 Mar 24.

4. Robb K, Wardle J, Stubbings S, Ramirez A, Austoker J, Macleod U, et al. Ethnic disparities in knowledge of cancer screening programmes in the UK. J Med Screen. 2010;17(3):125-31. doi: 10.1258/jms.2010.009112.

5. Robb KA, Power E, Atkin W, Wardle J. Ethnic differences in participation in flexible sigmoidoscopy screening in the UK. J Med Screen. 2008;15(3):130-6. doi: 10.1258/jms.2008.007112.

6. Austin KL, Power E, Solarin I, Atkin WS, Wardle J, Robb KA. Perceived barriers to flexible sigmoidoscopy screening for colorectal cancer among UK ethnic minority groups: a qualitative study. J Med Screen. 2009;16(4):174-9. doi: 10.1258/jms.2009.009080.

7. Lo SH, Waller J, Vrinten C, Kobayashi L, von Wagner C. Social Cognitive Mediators of Sociodemographic Differences in Colorectal Cancer Screening Uptake. Biomed Res Int. 2015;2015:165074. doi: 10.1155/2015/165074. Epub 2015 Oct 4.

8. Weller D, Coleman D, Robertson R, Butler P, Melia J, Campbell C, et al. The UK colorectal cancer screening pilot: results of the second round of screening in England. Br J Cancer. 2007 Dec 17;97(12):1601-5. Epub 2007 Nov 20.

9. von Wagner C, Baio G, Raine R, Snowball J, Morris S, Atkin W, et al. Inequalities in participation in an organized national colorectal cancer screening programme: results from

For peer review only - http://bmjopen.bmj.com/site/about/guidelines.xhtml

the first 2.6 million invitations in England. Int J Epidemiol. 2011 Jun;40(3):712-8. doi: 10.1093/ije/dyr008. Epub 2011 Feb 17.

10. Szczepura A, Johnson M, Orbell S, Gumber A, O'Sullivan I, Clay D, et al. Ethnicity: UK Colorectal Cancer Screening Pilot. 2003 https://webarchive.nationalarchives.gov.uk/20120511035604/http://www.cancerscreening.nhs.uk/bowel/ethnicity-finalreport.pdf *Accessed 23/08/19*

11. Lansdorp-Vogelaar I, Kuntz KM, Knudsen AB, van BM, Zauber AG, Jemal A. Contribution of screening and survival differences to racial disparities in colorectal cancer rates. Cancer Epidemiol Biomarkers Prev 2012 May;21(5):728-36

12. Price CL, Szczepura AK, Gumber AK, Patnick J. Comparison of breast and bowel cancer screening uptake patterns in a common cohort of South Asian women in England. BMC Health Serv Res. 2010 Apr 27;10:103.

13. Moss SM, Campbell C, Melia J, Coleman D, Smith S, Parker R, et al. Performance measures in three rounds of the English bowel cancer screening pilot. Gut. 2012 Jan;61(1):101-7. Epub 2011 May 10.

14. Logan RF, Patnick J, Nickerson C, Coleman L, Rutter MD, von Wagner C; on behalf of the English Bowel Cancer Screening Evaluation Committee. Outcomes of the Bowel Cancer Screening Programme (BCSP) in England after the first 1 million tests. Gut 2011 Dec 7. [Epub ahead of print]

15. Allaby M, Mulhall C, Lines C. Analyses of inequality in national screening programmes using existing data systems. A supplementary report for the UK National Screening Committee. Oxford: Public Health Resource Unit February 2009

16. Bhopal R, Fischbacher C, Povey C, Chalmers J, Mueller G, Steiner M, et al. Cohort profile: Scottish health and ethnicity linkage study of 4.65 million people exploring ethnic variations in disease in Scotland. Int J Epidemiol. 2011 Oct;40(5):1168-75. Epub 2010 Jul 24

17. Fischbacher CM, Bhopal R, Povey C, Steiner M, Chalmers J, Mueller G, et al. Record linked retrospective cohort study of 4.6 million people exploring ethnic variations in disease: myocardial infarction in South Asians. BMC Public Health. 2007 Jul 5;7:142.

For peer review only - http://bmjopen.bmj.com/site/about/guidelines.xhtml

18. Bansal N, Bhopal RS, Steiner MF, Brewster DH; Scottish Health and Ethnicity Linkage Study. Major ethnic group differences in breast cancer screening uptake in Scotland are not extinguished by adjustment for indices of geographical residence, area deprivation, long-term illness and education. Br J Cancer. 2012 Apr 10;106(8):1361-6. doi: 10.1038/bjc.2012.83. Epub 2012 Mar 13

19. National Records of Scotland 2001 Census Information https://www.nrscotland.gov.uk/statistics-and-data/census/2001-census *Accessed 23/08/19*

20. Boyd KM. Ethnicity and the ethics of data linkage. BMC Public Health 2007;7:318

21. de Klerk CM, Gupta S, Dekker E, Essink-Bot ML; Expert Working Group 'Coalition to reduce inequities in colorectal cancer screening' of the World Endoscopy Organization. Socioeconomic and ethnic inequities within organised colorectal cancer screening programmes worldwide. Gut. 2018 Apr;67(4):679-687. doi: 10.1136/gutjnl-2016-313311. Epub 2017 Jan 10.

22. Bhopal RS, Bansal N, Steiner M, Brewster DH; Scottish Health and Ethnicity Linkage Study. Does the 'Scottish effect' apply to all ethnic groups? All-cancer, lung, colorectal, breast and prostate cancer in the Scottish Health and Ethnicity Linkage Cohort Study. BMJ Open. 2012 Sep 25;2(5). pii: e001957. doi: 10.1136/bmjopen-2012-001957. Print 2012.

23. Cezard GI, Bhopal RS, Ward HJ, Bansal N, Bhala N; SHELS researchers. Ethnic variations in upper gastrointestinal hospitalizations and deaths: the Scottish Health and Ethnicity Linkage Study. Eur J Public Health. 2016 Apr;26(2):254-60. doi: 10.1093/eurpub/ckv182. Epub 2015 Oct 9.

24. Bhopal RS, Bansal N, Fischbacher C, Brown H, Capewell S; Scottish Health and Ethnicity Linkage Study (SHELS). Ethnic variations in chest pain and angina in men and women: Scottish Ethnicity and Health Linkage Study of 4.65 million people. Eur J Prev Cardiol. 2012 Dec;19(6):1250-7. doi: 10.1177/1741826711425775. Epub 2011 Oct 5

For peer review only

25. Bhopal RS, Cezard G, Bansal N, Ward HJ, Bhala N; SHELS researchers. Ethnic variations in five lower gastrointestinal diseases: Scottish health and ethnicity linkage study. BMJ Open. 2014 Oct 21;4(10):e006120. doi: 10.1136/bmjopen-2014-006120

26. National Records of Scotland Census 2011: Population Estimates for Scotland https://www.nrscotland.gov.uk/news/2012/census-2011-population-estimates-for-scotland *Accessed 23/08/19*

27. Census 2011: Detailed characteristics on Ethnicity, Identity, Language and Religion in Scotland – Release 3A https://www.scotlandscensus.gov.uk/news/census-2011-detailed-characteristics-ethnicity-identity-language-and-religion-scotland-%E2%80%93 *Accessed 23/08/19*

28. Gorman DR, Porteous LA. Influences on Polish migrants' breast screening uptake in Lothian, Scotland. Public Health. 2018 May;158:86-92. doi: 10.1016/j.puhe.2017.11.026. Epub 2018 Feb 13

29. Dharni N, Armstrong D, Chung-Faye G, Wright AJ. Factors influencing participation in colorectal cancer screening—a qualitative study in an ethnic and socio-economically diverse inner city population. Health Expectations. 2017 Aug;20(4):608-17

30. Vrinten C, Waller J, von Wagner C, Wardle J. Cancer fear: facilitator and deterrent to participation in colorectal cancer screening. Cancer Epidemiol Biomarkers Prev. 2015 Feb;24(2):400-5. doi: 10.1158/1055-9965.EPI-14-0967.

31. Taskila T, Wilson S, Damery S, Roalfe A, Redman V, Ismail T, et al. Factors affecting attitudes toward colorectal cancer screening in the primary care population. Br J Cancer. 2009 Jul 21;101(2):250-5. doi: 10.1038/sj.bjc.6605130. Epub 2009 Jun 23.

32. Palmer CK, Thomas MC, McGregor LM, von Wagner C, Raine R. Understanding low colorectal cancer screening uptake in South Asian faith communities in England--a qualitative study. BMC Public Health. 2015 Oct 1;15:998. doi: 10.1186/s12889-015-2334-9.

33. Padmanabhan H, Widlak M, Nevill A, McKaig B, Brookes M, Veitch A. Ethnic variation in colorectal cancer risk following a positive faecal occult blood test in an English bowel cancer

For peer review only

screening programme centre. Eur J Gastroenterol Hepatol. 2015 Nov;27(11):1281-5. doi: 10.1097/MEG.0000000000000443.

34. Trinh QD, Nguyen PL, Leow JJ, Dalela D, Chao GF, Mahal BA, et al. Cancer-specific mortality of Asian Americans diagnosed with cancer: a nationwide population-based assessment. J Natl Cancer Inst. 2015 Mar 20;107(6):djv054. doi: 10.1093/jnci/djv054. Print 2015 Jun.

35. Jerant A, To P, Franks P. The effects of tailoring knowledge acquisition on colorectal cancer screening self-efficacy. J Health Commun. 2015;20(6):697-709. doi: 10.1080/10810730.2015.1018562. Epub 2015 Apr 30.

36. Gaduputi V, Chandrala C, Tariq H, Sakam S, Dev A, Chilimuri S. Influence of perception of colorectal cancer risk and patient bowel preparation behaviors: a study in minority populations. Clin Exp Gastroenterol. 2015 Jan 28;8:69-75. doi: 10.2147/CEG.S75593. eCollection 2015.

37. McGregor LM, Bonello B, Kerrison RS, Nickerson C, Baio G, Berkman L, et al. Uptake of Bowel Scope (Flexible Sigmoidoscopy) Screening in the English National Programme: the first 14 months. J Med Screen. 2015 Sep 20. pii: 0969141315604659. [Epub ahead of print]

38. Shankleman J, Massat NJ, Khagram L, Ariyanayagam S, Garner A, Khatoon S, et al. Evaluation of a service intervention to improve awareness and uptake of bowel cancer screening in ethnically-diverse areas. Br J Cancer. 2014 Sep 23;111(7):1440-7. doi: 10.1038/bjc.2014.363. Epub 2014 Jul 1.

39. Devereux PG, Gray J, Robinson S, Galvin J, Gutierrez J. Using Community Engagement and Navigators to Increase Colon Cancer Screening and Patient Outcomes. Health Promot Pract. 2019 Jan;20(1):85-93. doi: 10.1177/1524839918757485. Epub 2018 Feb 9**.**

40. Hou SI, Cao X. A Systematic Review of Promising Strategies of Faith-Based Cancer Education and Lifestyle Interventions Among Racial/Ethnic Minority Groups. J Cancer Educ. 2018 Dec;33(6):1161-1175. doi: 10.1007/s13187-017-1277-5. Review

For peer review only - http://bmjopen.bmj.com/site/about/guidelines.xhtml

41. Leyva B, Nguyen AB, Allen JD, Taplin SH, Moser RP. Is Religiosity Associated with Cancer Screening? Results from a National Survey. J Relig Health. 2015 Jun;54(3):998-1013. doi: 10.1007/s10943-014-9843-1.

42. Davidson EM, Liu JJ, Bhopal R, White M, Johnson MR, Netto G, et al. Behavior change interventions to improve the health of racial and ethnic minority populations: a tool kit of adaptation approaches. Milbank Q. 2013 Dec;91(4):811-51. doi: 10.1111/1468-0009.12034. Review.

For peer review only

Table 1A. Age adjusted rates and risk ratios (RRs) for positive screen test results (Round 1) by ethnic group in men. RRs are adjusted for age, UK/RoI-born (versus born outside UK/RoI) and socio-economic status (household tenure and combined individual and household level education) with 95% confidence intervals (CIs).

				RRs (95% CIs): adjustment			
**Ethnic group**	**Positive screen test results**	**Completed screen kit returned**	**Rates/ 100,000**	**Age**	**Age and UK/RoI-born**	**Age and 2 socio-economic variables**	**Age, UK/RoI-born and 2 socio-economic variables**
MEN							
White Scottish	11100	362865	3060	100	100	100	100
Other White British	685	37040	1844	60.5 (56.1, 65.3)	60.9 (56.4, 65.7)	68.9 (63.8, 74.5)	69.2 (64.1, 74.7)
White Irish	130	4220	3081	98.6 (83.2, 116.8)	98.7 (83.3, 116.9)	99.5 (84, 117.8)	99.5 (84, 117.9)
Other White	110	4045	2770	96 (80, 115.2)	111 (90.3, 136.5)	104.6 (87.2, 125.5)	115.3 (93.3, 142.5)
Any Mixed Background	10	310	2913	101.6 (53.1, 194.5)	111.8 (58.4, 213.9)	108.3 (56.8, 206.6)	114.7 (60.2, 218.7)
Indian	15	705	1844	58.7 (34.3, 100.5)	71.6 (41.1, 124.6)	67.6 (39.5, 115.7)	76.9 (44.1, 134)
Pakistani	20	1015	2172	74.8 (49.9, 112.3)	92 (59.7, 141.6)	74.5 (49.6, 111.7)	85.3 (55.3, 131.6)
Other South Asian	10	400	2764	105.2 (59.1, 187)	126.9 (70.4, 228.6)	113.2 (64, 200.2)	127.3 (71.2, 227.7)
African origin	10	360	2500	97.4 (51.1, 185.7)	114.7 (59.4, 221.2)	102.5 (53.8, 195.4)	113.7 (58.9, 219.4)
Chinese	30	990	3128	114.2 (80.9, 161.3)	141.1 (96.7, 205.7)	109.4 (77.4, 154.5)	125.7 (86, 183.7)

For peer review only - http://bmjopen.bmj.com/site/about/guidelines.xhtml

Table 1B. Age adjusted rates and risk ratios (RRs) for positive screen test results (Round 1) by ethnic group in women. RRs are adjusted for age, UK/RoI-born (versus born outside UK/RoI) and socio-economic status (household tenure and combined individual and household level education) with 95% confidence intervals (CIs).

				RRs (95% CIs): adjustment			
**Ethnic group**	**Positive screen test results**	**Completed screen kit returned**	**Rates/ 100,000**	**Age**	**Age and UK/RoI-born**	**Age and 2 socio-economic variables**	**Age, UK/RoI-born and 2 socio-economic variables**
WOMEN							
White Scottish	8015	444425	1803	100	100	100	100
Other White British	510	42950	1187	67.3 (61.6, 73.5)	67.3 (61.5, 73.5)	76.4 (69.9, 83.5)	76.2 (69.7, 83.3)
White Irish	80	5255	1542	82.8 (66.7, 102.7)	82.8 (66.7, 102.7)	86.2 (69.5, 107)	86.2 (69.5, 107)
Other White	90	5840	1523	86.9 (70.7, 106.8)	86.8 (67.1, 112.3)	98.8 (80.3, 121.5)	92.8 (71.2, 120.8)
Any Mixed Background	10	435	2771	169.8 (96.7, 297.9)	169.6 (95.7, 300.7)	173.6 (99.4, 303.4)	167.5 (94.9, 295.4)
Indian	10	690	1744	100.8 (58.2, 174.5)	100.6 (57.1, 177.3)	110.5 (63.8, 191.4)	102.6 (58.1, 181.3)
Pakistani	15	870	1724	106.5 (64.6, 175.5)	106.2 (62.9, 179.3)	98.6 (59.8, 162.6)	91.2 (53.9, 154.5)
Other South Asian	.	265					
African origin	10	395	1515	98.2 (44.3, 217.6)	98.1 (44, 218.7)	107.2 (48.5, 236.9)	101.1 (45.4, 225.2)
Chinese	25	1210	2066	130.6 (88.9, 191.8)	130.3 (85, 199.9)	125.2 (85.4, 183.5)	115.2 (75, 177)

For peer review only

For peer review only - http://bmjopen.bmj.com/site/about/guidelines.xhtml

Table 2. Age adjusted rates and risk ratios (RRs) for screen detected invasive cancer by ethnic group (Rounds 1 & 2; men & women combined). Results exclude Grampian health board. RRs are adjusted for sex, age, UK/RoI-born (versus born outside UK/RoI) and socio-economic status (household tenure and combined individual and household level education) with 95% confidence intervals (CIs).

				RRs (95% CIs): adjustment			
**Ethnic group**	**Cancers**	**Invited into screening**	**Rates/ 100,000**	**Age**	**Age and UK/RoI-born**	**Age and 2 socio-economic variables**	**Age, UK/RoI-born and 2 socio-economic variables**
White Scottish	2025	2428585	83.4	100	100	100	100
Other White British	145	205420	70.5	84 (71, 99.3)	85 (71.8, 100.5)	79.5 (67.1, 94.1)	80.4 (67.9, 95.2)
White Irish	25	29770	77.3	86.5 (57.4, 130.4)	86.6 (57.5, 130.5)	86.7 (57.5, 130.6)	86.7 (57.5, 130.7)
Other White *	20	28620	71.2	92.5 (59.6, 143.7)	130 (71.5, 236.4)	88.9 (57.2, 138)	127.1 (70.4, 229.7)

* Results for Any Mixed Background, South Asian (Indian, Pakistan or other), or Chinese ethnic groups are not provided as they are so few as to be potentially disclosive

(see Methods)

For peer review only - http://bmjopen.bmj.com/site/about/guidelines.xhtml

Table 3A. Age adjusted rates and risk ratios (RRs) for bowel cancer screening uptake (Round 1) by ethnic group in men aged 75 years and over. RRs are adjusted for UK/RoI-born (versus born outside UK/RoI) and socio-economic status (household tenure and combined individual and household level education) with 95%

				RRs (95% CIs): adjustment			
**Ethnic group**	**Completed screen kit returned**	**Requested screening**	**Rates/ 100,000**	**Unadjusted**	**UK/RoI-born**	**2 socio-economic variables**	**UK/RoI-born and 2 socio-economic variables**
MEN							
White Scottish	2460	2780	88489	100	100	100	100
Other White British	180	215	84186	95 (89.5, 100.8)	94.7 (89.2, 100.5)	94.4 (88.8, 100.3)	94.2 (88.6, 100)
White Irish	30	30	87500	98.7 (86.6, 112.6)	98.5 (86.4, 112.3)	98.5 (86.3, 112.4)	98.3 (86.1, 112.2)
Other White	20	25	88000	99.3 (85.9, 114.8)	93.2 (78.9, 110.1)	98.2 (85, 113.4)	92.6 (78.6, 109.2)
Any Mixed Background	.	.					
South Asian	15	20	76190.5	86 (67.7, 109.2)	78.3 (60.8, 100.8)	85.1 (67.3, 107.7)	78.1 (60.8, 100.4)
Chinese	10	10	100000	112.8 (111.3, 114.3)	102.3 (93.6, 111.7)	115.1 (110.3, 120.1)	105.2 (95.3, 116)

For peer review only

For peer review only - http://bmjopen.bmj.com/site/about/guidelines.xhtml

Table 3B. Age adjusted rates and risk ratios (RRs) for bowel cancer screening uptake (Round 1) by ethnic group in women aged 75 years and over. RRs are adjusted for UK/RoI-born (versus born outside UK/RoI) and socio-economic status (household tenure and combined individual and household level education) with 95% confidence intervals (CIs).

				RRs (95% CIs): adjustment			
**Ethnic group**	**Complete d screen kit returned**	**Requested screening**	**Rates 100,000**	**Unadjusted**	**UK/RoI-born**	**2 socio-economic variables**	**UK/RoI-born and 2 socio-economic variables**
WOMEN							
White Scottish	2470	2885	85615.3	100	100	100	100
Other White British	170	195	87113.4	101.7 (96.1, 107.5)	101.6 (96.1, 107.5)	100.9 (95.3, 106.8)	100.9 (95.3, 106.8)
White Irish	30	30	90322.6	105.4 (93.9, 118.4)	105.4 (93.9, 118.4)	105.7 (94.1, 118.9)	105.7 (94.1, 118.9)
Other White	40	40	95000	110.9 (103.1, 119.2)	110.3 (99.7, 122.2)	110.3 (102.5, 118.7)	109.9 (99.2, 121.8)
Any Mixed Background	.	.					
South Asian	10	15	64705.9	75.5 (53.1, 107.3)	75.1 (52.2, 107.9)	75.3 (53.1, 106.8)	75 (52.3, 107.6)
Chinese	10	10	100000	116.7 (115, 118.5)	116 (104.3, 129)	117.7 (114.5, 121)	117.2 (105.1, 130.7)

For peer review only - http://bmjopen.bmj.com/site/about/guidelines.xhtml

For peer review only

FIGURE LEGENDS

Figure 1 **Age-adjusted rates ratios for uptake by ethnicity for Round 1 relative to the White Scottish population**

Figure 2 **Age-adjusted rates ratios for uptake by religion for Round 1 relative to the reference population (Church of Scotland)**

For peer review only

For peer review only - http://bmjopen.bmj.com/site/about/guidelines.xhtml

Campbell et al Ethnic and religious variations in participation in bowel cancer screening in Scotland

Figure 1 **Age-adjusted rates ratios for uptake by ethnicity for Round 1 relative to the White Scottish population**

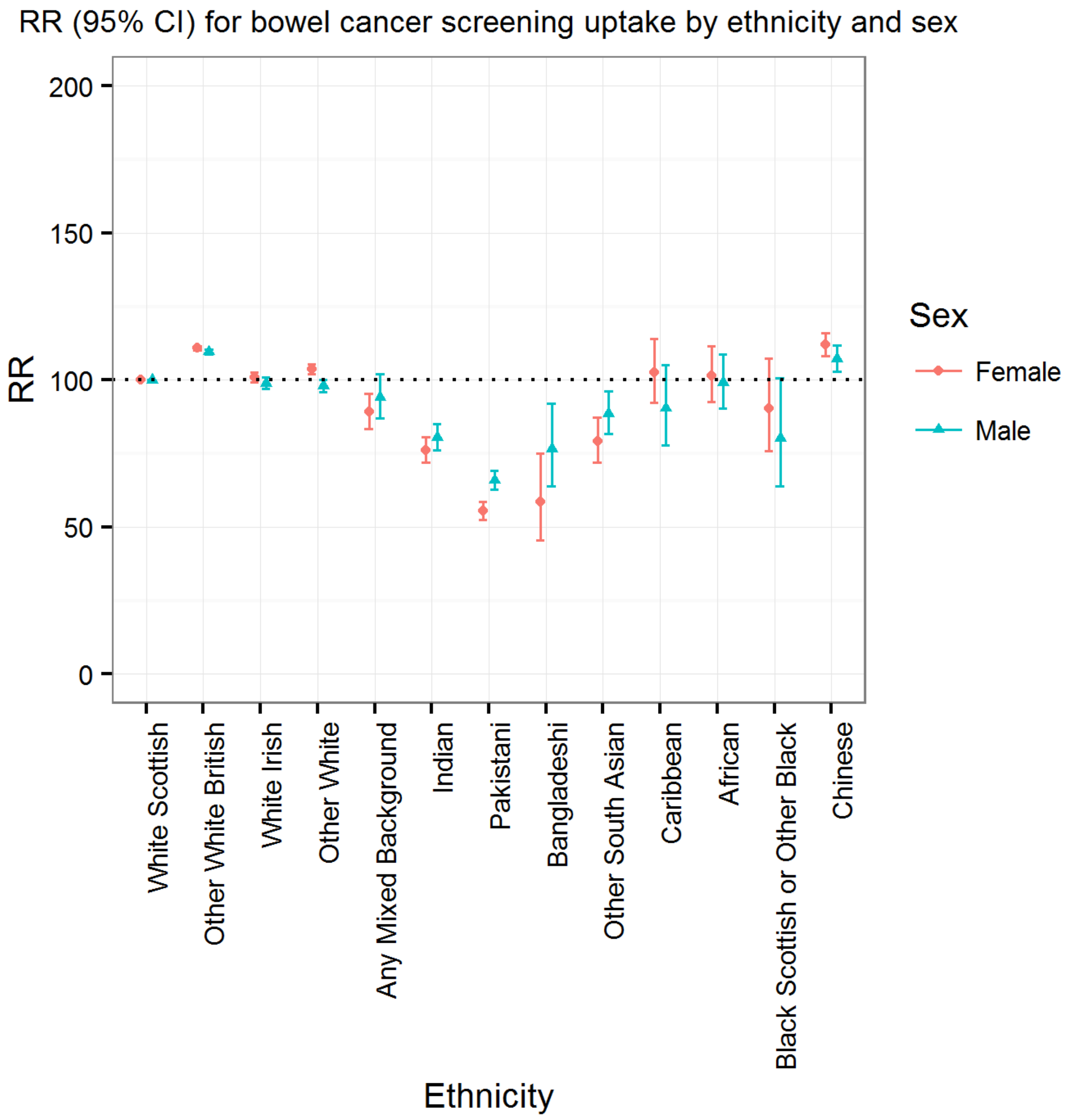

16/08/19

Campbell et al Ethnic and religious variations in participation in bowel cancer screening in Scotland

Figure 2 **Age-adjusted rates ratios for uptake by religion for Round 1 relative to the reference population (Church of Scotland)**

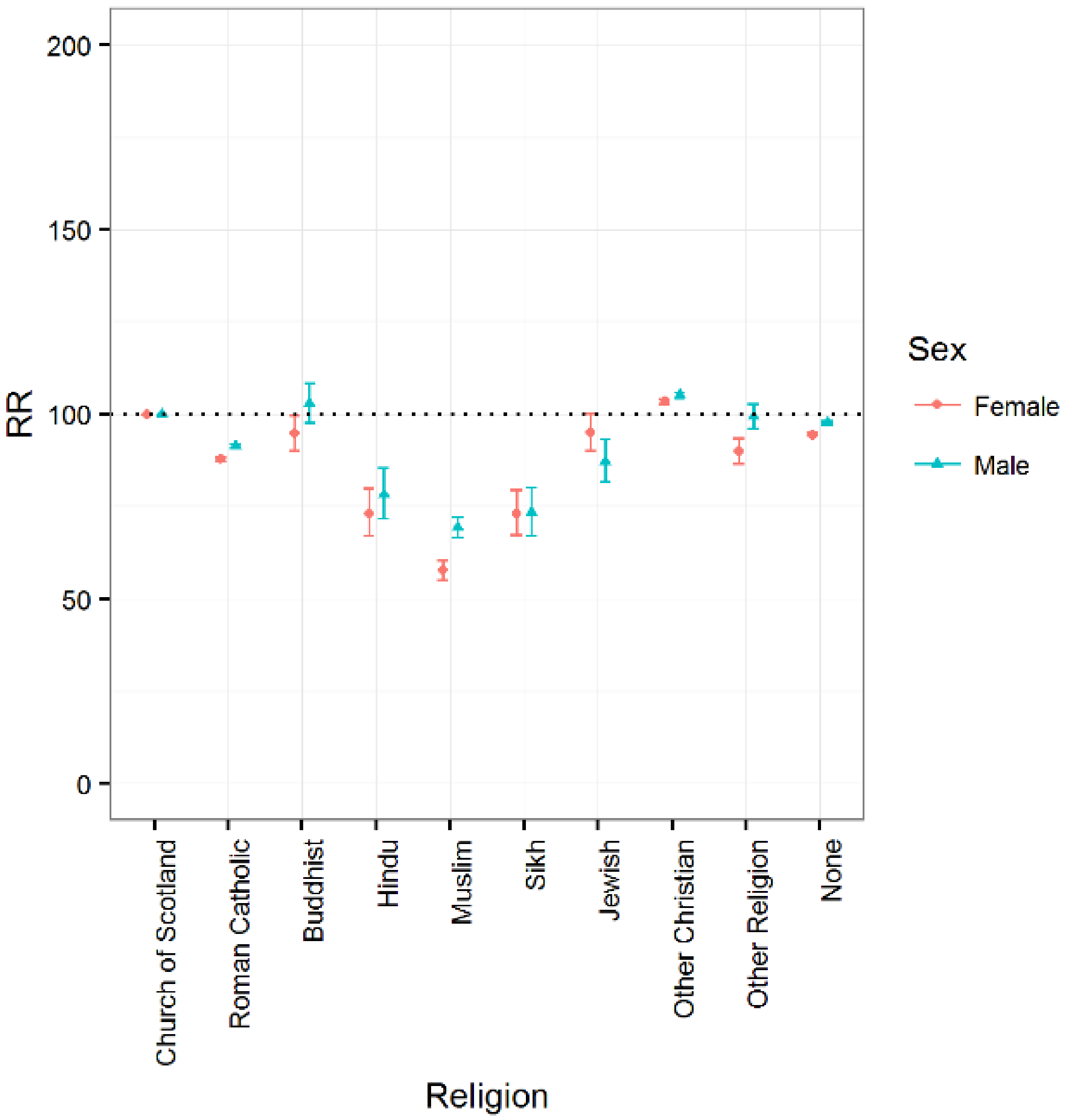

16/08/19

Supplementary Materials Tables for Campbell et al Ethic and religious variations in participation in bowel screening in Scotland

Supplementary Material Table 1A. Age adjusted rates and relative risks (RR) for bowel cancer screening uptake (Round 1) by ethnic group in men. RRs are adjusted for age, UK-born (versus born outside UK) and socio-economic status (household tenure and combined individual and household level education) with 95% confidence intervals (CIs).

				RRs (95% CIs): adjustment			
**Ethnic group**	**Completed screen kit returned**	**Invited into screening**	**Rates/ 100,000**	**Age**	**Age and UK/RoI-born**	**Age and 2 socio-economic variables**	**Age, UK/RoI-born and 2 socio-economic variables**
MEN							
White Scottish	362865	698715	51933	100	100	100	100
Other White British	37040	64855	57121	109.6 (108.8, 110.3)	109.5 (108.7, 110.2)	103 (102.2, 103.7)	102.9 (102.2, 103.7)
White Irish	4220	8155	51646	98.9 (96.9, 101)	98.9 (96.8, 101)	98.2 (96.2, 100.2)	98.2 (96.2, 100.2)
Other White	4045	8035	50320	98.1 (95.9, 100.2)	95.7 (93.4, 98.2)	94.7 (92.7, 96.8)	94.5 (92.2, 96.8)
Any Mixed Background	310	645	48094	94.1 (86.9, 102)	92.9 (85.7, 100.7)	94.5 (87.4, 102.3)	94.4 (87.2, 102.2)
Indian	705	1700	41495	80.5 (76.1, 85.1)	78 (73.6, 82.7)	75.4 (71.3, 79.7)	75.1 (70.9, 79.6)
Pakistani	1015	3040	33337	65.9 (62.7, 69.3)	63.7 (60.4, 67.2)	65.6 (62.4, 69)	65.4 (62, 68.9)
Bangladeshi	70	180	39013	76.6 (63.9, 91.9)	74.1 (61.8, 89)	77.9 (65.2, 93.1)	77.6 (64.9, 92.9)
Other South Asian	325	735	44492	88.6 (81.8, 96.1)	85.9 (79.1, 93.2)	86.4 (79.7, 93.7)	86.1 (79.4, 93.5)
Caribbean	90	200	45502	90.4 (77.7, 105.1)	88.5 (76.1, 102.9)	89.3 (76.7, 103.9)	89.1 (76.5, 103.7)
African	225	450	49560	99.1 (90.3, 108.7)	96.1 (87.5, 105.5)	99 (90.3, 108.6)	98.7 (89.9, 108.4)
Black Scottish or Other Black	45	110	40911	80.2 (64, 100.6)	79.2 (63.1, 99.4)	84 (67.2, 105)	83.9 (67.1, 104.9)
Chinese	990	1815	54579	107.2 (102.8, 111.8)	103.6 (99, 108.4)	107.4 (103, 112)	107 (102.3, 111.9)

170120

For peer review only - http://bmjopen.bmj.com/site/about/guidelines.xhtml

Supplementary Materials Tables for Campbell et al Ethic and religious variations in participation in bowel screening in Scotland

Supplementary Material Table 1B. Age adjusted rates and relative risks (RR) for bowel cancer screening uptake (round 1) by ethnic group in women. RRs are adjusted for age, UK-born (versus born outside UK) and socio-economic status (household tenure and combined individual and household level education) with 95% confidence intervals (CIs).

				RRs (95% CIs): adjustment			
**Ethnic group**	**Completed screen kit returned**	**Invited into screening**	**Rates/ 100000**	**Age**	**Age and UK/RoI-born**	**Age and 2 socio-economic variables**	**Age, UK/RoI-born and 2 socio-economic variables**
WOMEN							
White Scottish	444425	773555	57452	100	100	100	100
Other White British	42950	67160	63955	110.9 (110.2, 111.6)	110.8 (110.1, 111.4)	104.4 (103.8, 105.1)	104.4 (103.8, 105.1)
White Irish	5255	9005	58370	100.9 (99.2, 102.7)	100.9 (99.2, 102.7)	98.6 (96.9, 100.3)	98.6 (96.9, 100.3)
Other White	5840	9825	59472	103.8 (102.1, 105.5)	101 (99, 103)	97.8 (96.3, 99.4)	97.5 (95.6, 99.4)
Any Mixed Background	435	855	50564	89.2 (83.5, 95.4)	87.9 (82.3, 94)	89.2 (83.6, 95.2)	89 (83.4, 95.1)
Indian	690	1585	43409	76.1 (72, 80.5)	73.6 (69.5, 78)	72.3 (68.4, 76.4)	72 (68, 76.2)
Pakistani	870	2785	31233	55.5 (52.5, 58.6)	53.6 (50.6, 56.7)	56.7 (53.6, 59.9)	56.4 (53.3, 59.7)
Bangladeshi	40	130	32988	58.5 (45.6, 75.1)	56.7 (44.1, 72.8)	59.1 (46.2, 75.5)	58.8 (46, 75.2)
Other South Asian	225	500	45144	79.3 (72, 87.4)	77 (69.9, 84.8)	78.5 (71.5, 86.3)	78.2 (71.1, 86.1)
Caribbean	145	250	57998	102.6 (92.4, 113.9)	100.4 (90.4, 111.5)	97.3 (87.8, 107.8)	97 (87.5, 107.6)
African	190	330	57445	101.6 (92.6, 111.5)	98.4 (89.6, 108.1)	99.6 (90.8, 109.3)	99.2 (90.4, 109)
Black Scottish or Other Black	60	120	51665	90.2 (75.8, 107.2)	88.9 (74.7, 105.7)	92.7 (78.4, 109.6)	92.6 (78.3, 109.4)
Chinese	1210	1915	63190	112 (108.2, 115.9)	108 (104, 112.1)	111.7 (107.9, 115.6)	111.2 (107.1, 115.4)

170120

For peer review only

For peer review only - http://bmjopen.bmj.com/site/about/guidelines.xhtml

Supplementary Materials Tables for Campbell et al Ethic and religious variations in participation in bowel screening in Scotland

Supplementary Material Table 2A. Age adjusted rates and risk ratios (RRs) for bowel cancer screening uptake (Round 2) by ethnic group in men. RRs are adjusted for age, UK/RoI-born (versus born outside UK/RoI) and socio-economic status (household tenure and combined individual and household level education) with 95% confidence intervals (CIs).

				RRs (95% CIs): adjustment			
**Ethnic group**	**Completed screen kit returned**	**Invited into screening**	**Rates/ 100,000**	**Age**	**Age and UK/RoI-born**	**Age and 2 socio-economic variables**	**Age, UK/RoI-born and 2 socio-economic variables**
MEN							
White Scottish	324520	590540	54952.7	100	100	100	100
Other White British	33190	55265	60056.8	108.5 (107.5, 109.6)	108.4 (107.3, 109.4)	102.4 (101.2, 103.7)	102.4 (101.2, 103.6)
White Irish	3770	6850	55073	99.7 (97.5, 101.9)	99.6 (97.5, 101.9)	99.2 (97, 101.4)	99.2 (97, 101.4)
Other White	3595	6650	54091.4	108 (91.8, 127.1)	104.3 (90.9, 119.6)	103.7 (89.5, 120.1)	102.3 (90.4, 115.8)
Any Mixed Background	265	505	52870.6	96.9 (89.2, 105.3)	95 (87.3, 103.4)	97.4 (89.7, 105.7)	96.6 (88.9, 105)
Indian	645	1440	44729	81.3 (76.8, 86.2)	77.6 (72.3, 83.3)	77.1 (72.8, 81.6)	75.7 (70.6, 81.1)
Pakistani	905	2565	35298.3	65.2 (61.7, 68.8)	62 (57.7, 66.7)	65.6 (62.1, 69.2)	64.3 (59.7, 69.2)
Bangladeshi	60	145	41666.1	76.5 (62.9, 92.9)	72.8 (59.6, 88.8)	77.1 (63.9, 93)	75.6 (62.4, 91.6)
Other South Asian	295	620	47895.8	89 (81.8, 96.8)	84.9 (77.4, 93.3)	87.1 (80.1, 94.6)	85.5 (78, 93.7)
Caribbean	75	160	46874.4	86.6 (73.5, 102.2)	83.8 (70.8, 99.1)	85.4 (72.4, 100.8)	84.3 (71.3, 99.7)
African	180	345	51872.5	96.1 (86.6, 106.6)	91.8 (82.1, 102.5)	95.7 (86.3, 106)	94 (84.2, 104.8)
Black Scottish or Other Black	45	90	46738.5	86.2 (69.3, 107.3)	84.6 (68, 105.3)	89.7 (72.3, 111.2)	89 (71.8, 110.4)
Chinese	890	1505	59162.6	108 (103.2, 113.1)	102.7 (96.3, 109.6)	109.6 (104.7, 114.8)	107.4 (100.5, 114.9)

170120

For peer review only

Supplementary Materials Tables for Campbell et al Ethic and religious variations in participation in bowel screening in Scotland

Supplementary Material Table 2B. Age adjusted rates and risk ratios (RRs) for bowel cancer screening uptake (Round 2) by ethnic group in women. RRs are adjusted for age, UK/RoI-born (versus born outside UK/RoI) and socio-economic status (household tenure and combined individual and household level education) with 95% confidence intervals (CIs).

				RRs (95% CIs): adjustment			
**Ethnic group**	**Completed screen kit returned**	**Invited into screening**	**Rates/ 100.000**	**Age**	**Age and UK/RoI-born**	**Age and 2 socio-economic variables**	**Age, UK/RoI-born and 2 socio-economic variables**
WOMEN							
White Scottish	388730	654990	59348.8	100	100	100	100
Other White British	37600	57050	66369.9	110.7 (110, 111.4)	110.4 (109.8, 111.1)	104.5 (103.9, 105.1)	104.4 (103.8, 105.1)
White Irish	4610	7625	60891.6	101 (99.2, 102.9)	101 (99.2, 102.9)	98.9 (97.1, 100.7)	98.9 (97.1, 100.7)
Other White	5090	8215	107045.9	104.7 (102.9, 106.5)	100.2 (98.2, 102.3)	98.8 (97.2, 100.5)	97 (95, 98.9)
Any Mixed Background	390	710	55407.6	94.1 (88, 100.5)	91.8 (85.8, 98.1)	94.3 (88.3, 100.6)	93.2 (87.3, 99.5)
Indian	620	1355	45997	77.6 (73.3, 82.2)	73.5 (69.3, 78)	73.8 (69.6, 78.1)	72 (67.9, 76.4)
Pakistani	795	2330	34324.4	58.9 (55.6, 62.3)	55.6 (52.5, 59)	59.9 (56.7, 63.4)	58.5 (55.2, 62)
Bangladeshi	40	110	35432.3	60.7 (46.8, 78.7)	57.6 (44.4, 74.7)	60.4 (46.9, 77.8)	59 (45.8, 76.1)
Other South Asian	200	405	49852.6	84.2 (76.4, 92.9)	80.2 (72.7, 88.6)	83.1 (75.3, 91.6)	81.3 (73.6, 89.7)
Caribbean	120	210	57819.5	98.2 (87.5, 110.2)	94.6 (84.2, 106.1)	93.2 (83.1, 104.6)	91.7 (81.7, 102.8)
African	165	270	62375.3	105.8 (96.3, 116.2)	100.4 (91.3, 110.4)	103.1 (93.7, 113.5)	100.8 (91.5, 111.1)
Black Scottish or Other Black	45	95	49809.7	83.8 (68.6, 102.4)	81.7 (66.9, 99.8)	84.9 (70.1, 102.8)	83.9 (69.2, 101.6)
Chinese	1080	1605	67867.3	115.7 (111.8, 119.8)	109.1 (105.1, 113.3)	115.2 (111.3, 119.2)	112.2 (108.1, 116.5)

170120

For peer review only

Supplementary Materials Tables for Campbell et al Ethic and religious variations in participation in bowel screening in Scotland

Supplementary Material Table 3A. Age adjusted rates and risk ratios (RRs) for bowel cancer screening uptake (Round 1) by country of birth in men. RRs are adjusted for age and socio-economic status (household tenure and combined individual and household level education) with 95% confidence intervals (CIs).

				RRs (95% CIs): adjustment	
**Country of birth**	**Completed screen kit returned**	**Invited into screening**	**Rates/ 100,000**	**Age**	**Age and 2 socio-economic variables**
MEN					
Scotland	355310	685975	51796.4	100	100
Northern Ireland	2720	4890	55376.5	107 (104.4, 109.7)	102.9 (100.4, 105.4)
Rep Ireland	1695	3240	52218.9	97.8 (94.6, 101.1)	99.3 (96.2, 102.6)
Other UK	42015	73785	56952.8	109.6 (108.9, 110.3)	103.2 (102.5, 103.9)
India	1070	2240	47726.8	90.1 (86.3, 94)	83.8 (80.3, 87.4)
Pakistan	855	2670	32067.9	63.8 (60.4, 67.4)	63.5 (60.1, 67.1)
Bangladesh	80	195	40936.1	81 (68.4, 96.1)	80.9 (68.4, 95.6)
China	185	345	53606.9	102.3 (92.8, 112.8)	100.1 (90.9, 110.2)
Hong Kong	815	1470	55631.6	110 (105.1, 115.2)	110 (105.1, 115.1)
Africa	1715	3330	51506.8	102.1 (98.8, 105.5)	96.2 (93.1, 99.4)
Caribbean and West Indies	130	245	52873.4	102.8 (91.5, 115.5)	96.2 (85.6, 108)
Rest of the World	5740	11135	51544.3	101.6 (99.8, 103.5)	97.7 (96, 99.5)

170120

Supplementary Materials Tables for Campbell et al Ethic and religious variations in participation in bowel screening in Scotland

Supplementary Material Table 3B. Age adjusted rates and risk ratios (RRs) for bowel cancer screening uptake (Round 1) by country of birth in women. RRs are adjusted for age and socio-economic status (household tenure and combined individual and household level education) with 95% confidence intervals (CIs).

Country of birth	Completed screen kit returned	Invited into screening	Rates/ 100,000	RRs (95% CIs): adjustment<br>Age	RRs (95% CIs): adjustment<br>Age and 2 socio-economic variables
WOMEN					
Scotland	435740	760005	57333.7	100	100
Northern Ireland	3410	5490	62120	108.1 (105.9, 110.4)	103.4 (101.4, 105.6)
Rep Ireland	2615	4365	59889.6	102.8 (100.3, 105.3)	101.7 (99.3, 104.1)
Other UK	47935	75540	63453.7	110.4 (109.7, 111)	104.3 (103.7, 104.9)
India	1065	2110	50385.7	86.9 (83.3, 90.6)	81.1 (77.8, 84.6)
Pakistan	780	2455	31807.7	56.6 (53.4, 60)	57.8 (54.6, 61.2)
Bangladesh	45	120	38227.6	67.8 (53.9, 85.4)	66.9 (53.3, 83.9)
China	275	435	62469.5	110.7 (103, 119.1)	108.5 (101, 116.7)
Hong Kong	850	1350	62987.5	112.6 (108, 117.3)	112.9 (108.4, 117.7)
Africa	1860	3110	59869.6	106 (103, 109.1)	99.5 (96.7, 102.4)
Caribbean and West Indies	220	335	66064.1	115.8 (107.3, 125.1)	105.7 (97.9, 114.1)
Rest of the World	8115	13755	59017.1	104.1 (102.7, 105.6)	98.9 (97.5, 100.2)

170120

Supplementary Materials Tables for Campbell et al Ethic and religious variations in participation in bowel screening in Scotland

Supplementary Material Table 4A. Age adjusted rates and risk ratios (RRs) for bowel cancer screening uptake (Round 1) by religion of upbringing in men. RRs are adjusted for age, UK/RoI-born (versus born outside UK/RoI) and socio-economic status (household tenure and combined individual and household level education) with 95% confidence intervals (CIs).

				RRs (95% CIs): adjustment			
**Religion of upbringing**	**Completed screen kit returned**	**Invited into screening**	**Rates/ 100,000**	**Age**	**Age and UK/RoI-born**	**Age and 2 socio-economic variables**	**Age, UK/RoI-born and 2 socio-economic variables**
MEN							
Church of Scotland	243955	450020	54209.6	100	100	100	100
Roman Catholic	66100	134685	48802.5	91.9 (91.3, 92.4)	91.8 (91.2, 92.3)	94.6 (94, 95.1)	94.6 (94, 95.1)
Buddhist	250	495	50604.3	94.8 (86.9, 103.4)	91.9 (84.2, 100.3)	96.2 (88.2, 105)	96.1 (88, 105)
Hindu	325	730	44185.5	81.2 (74.9, 88)	78.5 (72.3, 85.2)	74.3 (68.5, 80.5)	74.2 (68.3, 80.6)
Muslim	1615	4410	36626.2	70 (67.4, 72.8)	67.7 (64.9, 70.5)	70.4 (67.7, 73.2)	70.4 (67.5, 73.3)
Sikh	310	800	38923.2	73.6 (67.5, 80.3)	71.5 (65.6, 78.1)	72.2 (66.3, 78.7)	72.2 (66.2, 78.7)
Jewish	585	1240	46939.9	86.3 (81.4, 91.6)	86.1 (81.1, 91.3)	81.6 (77, 86.6)	81.6 (77, 86.6)
Other Christian	42550	72280	58868.5	108.2 (107.5, 108.9)	108 (107.3, 108.7)	103 (102.3, 103.7)	103 (102.3, 103.7)
Other Religion	370	825	44848	85.2 (79, 91.9)	84.8 (78.6, 91.4)	84.3 (78.3, 90.9)	84.3 (78.3, 90.9)
None	38330	83260	46036.6	88.1 (87.4, 88.8)	88 (87.3, 88.7)	91.5 (90.8, 92.2)	91.5 (90.8, 92.2)

170120

Supplementary Materials Tables for Campbell et al Ethic and religious variations in participation in bowel screening in Scotland

Supplementary Material Table 4B. Age adjusted rates and risk ratios (RRs) for bowel cancer screening uptake (Round 1) by religion of upbringing in women. RRs are adjusted for age, UK/RoI-born (versus born outside UK/RoI) and socio-economic status (household tenure and combined individual and household level education) with 95% confidence intervals (CIs).

				RRs (95% CIs): adjustment			
**Religion of upbringing**	**Completed screen kit returned**	**Invited into screening**	**Rates/ 100,000**	**Age**	**Age and UK/RoI-born**	**Age and 2 socio-economic variables**	**Age, UK/RoI-born and 2 socio-economic variables**
WOMEN							
Church of Scotland	300940	499325	60269	100	100	100	100
Roman Catholic	83420	156940	53154	88.9 (88.4, 89.4)	88.8 (88.3, 89.2)	91.5 (91.1, 92)	91.5 (91, 92)
Buddhist	400	710	56336.8	95.4 (89.4, 101.8)	90.1 (84.4, 96.2)	96.5 (90.5, 102.9)	95 (89, 101.4)
Hindu	280	645	43584.8	72.4 (66.3, 79)	68.3 (62.5, 74.6)	67.1 (61.5, 73.3)	66.1 (60.5, 72.2)
Muslim	1090	3265	33363.3	56.5 (53.8, 59.3)	53.3 (50.7, 56.1)	58.4 (55.6, 61.2)	57.4 (54.7, 60.4)
Sikh	340	790	43061.2	72.3 (66.7, 78.3)	68.9 (63.6, 74.7)	72.7 (67.2, 78.8)	71.8 (66.2, 77.8)
Jewish	705	1235	57014.2	93.7 (89.2, 98.3)	92.9 (88.5, 97.5)	88.4 (84.3, 92.8)	88.2 (84.1, 92.6)
Other Christian	55265	85070	64964.8	107.4 (106.9, 108)	107.1 (106.5, 107.7)	102.9 (102.4, 103.5)	102.8 (102.3, 103.4)
Other Religion	365	700	51924.7	87.2 (81.2, 93.6)	86.3 (80.4, 92.6)	88.6 (82.6, 95)	88.3 (82.4, 94.7)
None	34110	68420	49855.9	85 (84.3, 85.6)	84.8 (84.1, 85.5)	90.3 (89.6, 91)	90.2 (89.5, 90.9)

170120

For peer review only - http://bmjopen.bmj.com/site/about/guidelines.xhtml

Supplementary Materials Tables for Campbell et al Ethic and religious variations in participation in bowel screening in Scotland

Supplementary Material Table 5A. Age adjusted rates and risk ratios (RRs) for positive screen test results (Round 2) by ethnic group in men. RRs are adjusted for age, UK/RoI-born (versus born outside UK/RoI) and socio-economic status (household tenure and combined individual and household level education) with 95% confidence intervals (CIs).

				RRs (95% CIs): adjustment			
**Ethnic group**	**Positive screen test results**	**Completed screen kit returned**	**Rates/ 100,000**	**Age**	**Age and UK/RoI-born**	**Age and 2 socio-economic variables**	**Age, UK/RoI-born and 2 socio-economic variables**
MEN							
White Scottish	10540	324520	3248.2	100	100	100	100
Other White British	705	33190	2124	65.3 (60.5, 70.4)	65.5 (60.8, 70.6)	74.3 (68.8, 80.2)	74.4 (68.9, 80.3)
White Irish	145	3770	3870.6	117.2 (100.1, 137.3)	117.3 (100.1, 137.3)	118 (100.8, 138.2)	118 (100.8, 138.2)
Other White	105	3595	2892.1	91.8 (75.9, 110.9)	101.2 (80.6, 126.9)	100 (82.7, 120.9)	104.7 (82.9, 132.1)
Any Mixed Background	10	265	4494.3	144.4 (83, 251.3)	152.4 (87, 267.1)	146.8 (84, 256.6)	150.3 (85.4, 264.6)
Indian	20	645	2790.7	86.3 (54.6, 136.4)	98.5 (60.5, 160.3)	100.1 (63.5, 157.9)	106.3 (65.4, 172.8)
Pakistani	25	905	2761.6	85.1 (57.6, 125.5)	97.8 (63.6, 150.4)	84.1 (57, 124.1)	89.6 (58.2, 138.2)
Other South Asian	10	355	3370.8	120 (68.8, 209.2)	137 (76.8, 244.3)	134.5 (77, 234.8)	142.7 (80, 254.6)
African origin	10	300	3020.1	107.3 (56.3, 204.2)	119.9 (62.1, 231.2)	113.9 (59.8, 216.9)	119.8 (62.1, 231)
Chinese	30	890	3254.7	111.8 (78.6, 159.1)	128.7 (87, 190.3)	107.6 (75.8, 152.8)	114.8 (77.6, 169.8)

170120

For peer review only - http://bmjopen.bmj.com/site/about/guidelines.xhtml

Supplementary Materials Tables for Campbell et al Ethic and religious variations in participation in bowel screening in Scotland

Supplementary Material Table 5B. Age adjusted rates and risk ratios (RRs) for positive screen test results (Round 2) by ethnic group in women. RRs are adjusted for age, UK/RoI-born (versus born outside UK/RoI) and socio-economic status (household tenure and combined individual and household level education) with 95% confidence intervals (CIs).

				RRs (95% CIs): adjustment			
**Ethnic group**	**Positive screen test results**	**Completed screen kit returned**	**Rates/ 100,000**	**Age**	**Age and UK/RoI-born**	**Age and 2 socio-economic variables**	**Age, UK/RoI-born and 2 socio-economic variables**
WOMEN							
White Scottish	8050	388730	2070.9	100	100	100	100
Other White British	490	37600	1300.5	63.8 (58.3, 69.8)	64.2 (58.6, 70.2)	71.6 (65.4, 78.4)	71.8 (65.6, 78.7)
White Irish	90	4610	1973.9	91.8 (75, 112.3)	91.8 (75, 112.4)	93.9 (76.7, 114.9)	93.9 (76.7, 114.9)
Other White	70	5090	1375.5	68.3 (54.2, 86.2)	78.7 (60, 103.3)	76.8 (60.9, 96.9)	84 (63.7, 110.8)
Any Mixed Background	10	390	2561.7	132.2 (72.5, 241)	143.8 (77.9, 265.3)	134.2 (73.2, 246.1)	140.9 (76.1, 261.1)
Indian	10	620	1291.6	64.5 (32.2, 129)	77.1 (37.3, 159.3)	69.9 (35, 139.6)	78.1 (37.8, 161.3)
Pakistani	20	795	2644.8	141.7 (92.6, 216.9)	169.7 (107.5, 268)	132.7 (86.8, 202.8)	148.6 (93.8, 235.4)
Other South Asian	.	240					
African origin	10	330	2108.4	109.7 (52.6, 228.8)	127 (60.4, 267.4)	119.1 (57.3, 247.5)	130.4 (62.1, 273.6)
Chinese	20	1080	2032.6	112.6 (74.3, 170.7)	136.5 (85.8, 217.2)	108.5 (71.5, 164.7)	122.5 (76.7, 195.8)

170120

Supplementary Materials Tables for Campbell et al Ethic and religious variations in participation in bowel screening in Scotland

Supplementary Material Table 6A. Age adjusted rates and risk ratios (RRs) for colonoscopy performed (Round 1) by ethnic group in men. Results exclude Grampian health board. RRs are adjusted for age, UK/RoI-born (versus born outside UK/RoI) and socio-economic status (household tenure and combined individual and household level education) with 95% confidence intervals (CIs).

				RRs (95% CIs): adjustment			
**Ethnic group**	**Colonoscopy performed**	**Positive screen test results**	**Rates/ 100,000**	**Age**	**Age and UK/RoI-born**	**Age and 2 socio-economic variables**	**Age, UK/RoI-born and 2 socio-economic variables**
MEN							
White Scottish	8380	10125	82765.4	100	100	100	100
Other White British	495	595	83172.7	100.6 (98.1, 103.1)	100.5 (98, 103)	100 (97.5, 102.5)	99.9 (97.4, 102.4)
White Irish	100	125	78144.9	94.5 (88, 101.5)	94.6 (88.1, 101.5)	94.7 (88.2, 101.7)	94.7 (88.2, 101.7)
Other White	80	95	83447.6	100.8 (95, 107)	97.6 (91.4, 104.2)	100.5 (94.7, 106.7)	97.5 (91.4, 104.1)
Any Mixed Background	.	10					
Indian	10	10	89866.7	108.9 (107.9, 109.8)	103.6 (99.7, 107.7)	108 (106.8, 109.1)	103.2 (99.2, 107.4)
Pakistani	15	20	76386.7	92.2 (76.7, 111)	88 (73, 106.2)	92 (76.3, 110.9)	88.2 (72.9, 106.5)
Other South Asian	10	10	79881.5	96.5 (76.4, 121.8)	92.8 (73.4, 117.4)	96.8 (76.3, 122.7)	93.4 (73.5, 118.7)
African origin	10	10	77028.6	93.1 (68.8, 125.9)	90.5 (67, 122.2)	93.1 (68.4, 126.6)	90.7 (66.8, 123.2)
Chinese	20	30	81307.9	98.3 (85.6, 112.9)	93.8 (81.4, 108.2)	98.1 (85.6, 112.5)	94 (81.6, 108.2)

170120

For peer review only

Supplementary Materials Tables for Campbell et al Ethic and religious variations in participation in bowel screening in Scotland

Supplementary Material Table 6B. Age adjusted rates and risk ratios (RRs) for colonoscopy performed (Round 1) by ethnic group in women. Results exclude Grampian health board. RRs are adjusted for age, UK/RoI-born (versus born outside UK/RoI) and socio-economic status (household tenure and combined individual and household level education) with 95% confidence intervals (CIs).

				RRs (95% CIs): adjustment			
**Ethnic group**	**Colonoscopy performed**	**Positive screen test results**	**Rates/ 100,000**	**Age**	**Age and UK/RoI-born**	**Age and 2 socio-economic variables**	**Age, UK/RoI-born and 2 socio-economic variables**
WOMEN							
White Scottish	5870	7295	80466.1	100	100	100	100
Other White British	365	450	78820.6	98 (94.6, 101.4)	97.7 (94.3, 101.2)	97.4 (94.1, 100.9)	97.2 (93.9, 100.7)
White Irish	60	80	81980.5	102 (95.2, 109.2)	101.7 (95, 108.9)	102.5 (95.7, 109.7)	102.2 (95.5, 109.5)
Other White	60	85	75242.4	93.3 (84.7, 102.8)	89.7 (80.2, 100.4)	92.6 (84.1, 102)	89.3 (79.8, 99.9)
Any Mixed Background	10	10	77517.8	97.1 (74.6, 126.2)	95.6 (73.7, 124.1)	99 (76.3, 128.5)	97.6 (75.4, 126.4)
Indian	10	10	70873.4	87.9 (64.6, 119.6)	82.7 (60.3, 113.3)	86.6 (64.1, 116.9)	81.7 (60, 111.2)
Pakistani	10	15	88591.8	109.8 (108.2, 111.4)	103.3 (96.3, 110.8)	109.7 (107.3, 112.2)	103.5 (96.3, 111.3)
Other South Asian	.	.					
African origin	.	10					
Chinese	15	25	78820.6	93.3 (77.5, 112.3)	87.7 (72, 106.9)	92.3 (76.6, 111.2)	87.1 (71.4, 106.2)

170120

For peer review only

Supplementary Materials Tables for Campbell et al Ethic and religious variations in participation in bowel screening in Scotland

Supplementary Material Table 6C. Age adjusted rates and risk ratios (RRs) for colonoscopy performed (Round 2) by ethnic group in men. Results exclude Grampian health board. RRs are adjusted for age, UK/RoI-born (versus born outside UK/RoI) and socio-economic status (household tenure and combined individual and household level education) with 95% confidence intervals (CIs).

				RRs (95% CIs): adjustment			
**Ethnic group**	**Colonoscopy performed**	**Positive screen test results**	**Rates**	**Age/ 100,000**	**Age and UK/RoI-born**	**Age and 2 socio-economic variables**	**Age, UK/RoI-born and 2 socio-economic variables**
MEN							
White Scottish	7935	9505	83482.4	100	100	100	100
Other White British	505	595	82759.7	99.3 (96.7, 101.9)	99.3 (96.7, 101.9)	98.8 (96.2, 101.5)	98.8 (96.2, 101.5)
White Irish	115	140	84825.9	101.7 (97.1, 106.5)	101.7 (97.1, 106.5)	102 (97.4, 106.9)	102 (97.4, 106.9)
Other White	85	95	84584.3	100.9 (95.5, 106.7)	101.3 (95.2, 107.7)	100.7 (95.1, 106.5)	101.4 (95.2, 108)
Any Mixed Background	10	10	90626	108.4 (107.4, 109.5)	108.6 (107.1, 110.1)	108.8 (107.2, 110.5)	109.1 (107.1, 111.1)
Indian	15	15	84961.9	101.3 (89.3, 115)	101.8 (89.3, 116.1)	101.1 (89.6, 114.1)	102.2 (90, 116)
Pakistani	20	25	78805.2	93.8 (80, 110.1)	94.3 (80.1, 110.9)	93.3 (79.5, 109.6)	94.3 (80.1, 111)
Other South Asian	10	10	90626	108.1 (107, 109.1)	108.6 (103.5, 113.9)	107.9 (105.9, 109.9)	109.1 (103.7, 114.6)
African origin	10	10	90626	107.4 (106.4, 108.5)	107.8 (103.7, 112.2)	107.8 (105.5, 110.2)	108.8 (104.1, 113.6)
Chinese	25	25	90626	108.3 (107.5, 109.2)	108.9 (103.6, 114.5)	108.6 (107.3, 109.9)	109.9 (104.4, 115.6)

170120

For peer review only

Supplementary Materials Tables for Campbell et al Ethic and religious variations in participation in bowel screening in Scotland

Supplementary Material Table 6D. Age adjusted rates and risk ratios (RRs) for colonoscopy performed (Round 2) by ethnic group in women. Results exclude Grampian health board. RRs are adjusted for age, UK/RoI-born (versus born outside UK/RoI) and socio-economic status (household tenure and combined individual and household level education) with 95% confidence intervals (CIs).

				RRs (95% CIs): adjustment			
**Ethnic group**	**Colonoscopy performed**	**Positive screen test results**	**Rates/ 100,000**	**Age**	**Age and UK/RoI-born**	**Age and 2 socio-economic variables**	**Age, UK/RoI-born and 2 socio-economic variables**
WOMEN							
White Scottish	6080	7355	82664.9	100	100	100	100
Other White British	365	435	82495	99.8 (96.8, 102.9)	99.9 (96.9, 103)	99.4 (96.4, 102.5)	99.5 (96.5, 102.6)
White Irish	70	85	79995.2	96.9 (89.7, 104.8)	96.9 (89.6, 104.8)	96.8 (89.5, 104.7)	96.8 (89.5, 104.7)
Other White	55	65	84093.3	101.8 (95.2, 108.8)	104 (94.9, 114)	101.9 (95.3, 109)	104.3 (95.2, 114.1)
Any Mixed Background	10	10	89994.6	109.1 (107.8, 110.3)	110.1 (106.7, 113.5)	109.2 (107.9, 110.6)	110.3 (106.9, 113.8)
Indian	10	10	89994.6	107.6 (106.4, 108.8)	110.1 (102.7, 118.2)	107.1 (105.4, 108.8)	109.7 (102.2, 117.8)
Pakistani	15	20	64996.1	78.1 (58.6, 104.1)	80.6 (59.6, 108.9)	78.1 (58.7, 103.9)	80.6 (59.7, 108.9)
Other South Asian	.	.					
African origin	.	10					
Chinese	20	20	85258	102.6 (92.2, 114.1)	105.6 (92.1, 121.1)	101.8 (91.5, 113.3)	105 (91.6, 120.4)

170120

For peer review only

Supplementary Materials Tables for Campbell et al Ethic and religious variations in participation in bowel screening in Scotland

Supplementary Material Table 7A. Age adjusted rates and risk ratios (RRs) for pathology detected (polyps, adenoma, cancer) (Rounds 1 & 2) by ethnic group in men. Results exclude Grampian health board. RRs are adjusted for age, UK/RoI-born (versus born outside UK/RoI) and socio-economic status (household tenure and combined individual and household level education) with 95% confidence intervals (CIs).

				RRs (95% CIs): adjustment			
**Ethnic group**	**Pathology detected**	**Positive screen test results**	**Rates**	**Age/ 100,000**	**Age and UK/RoI-born**	**Age and 2 socio-economic variables**	**Age, UK/RoI-born and 2 socio-economic variables**
MEN							
White Scottish	10140	19630	51655.6	100	100	100	100
Other White British	615	1190	51922	100 (94.4, 105.8)	99.9 (94.4, 105.8)	97 (91.6, 102.7)	97 (91.5, 102.7)
White Irish	140	265	52073.3	100 (89, 112.4)	100 (89.1, 112.4)	100.9 (89.9, 113.3)	100.9 (89.9, 113.3)
Other White	95	190	50291.3	98.3 (85.3, 113.3)	96.8 (82, 114.3)	96.4 (83.7, 111.1)	96.2 (81.6, 113.4)
Any Mixed Background	10	15	46756.9	90.3 (56.4, 144.6)	89.7 (55.8, 144.1)	91 (57, 145.1)	90.9 (56.9, 145.2)
Indian	15	30	48832.3	95.6 (65.8, 139.1)	93.4 (63, 138.7)	93.3 (64.2, 135.5)	93 (62.7, 138)
Pakistani	15	45	32456.4	64.5 (42.5, 97.7)	63 (40.8, 97.4)	64.2 (42.5, 97)	64.1 (41.6, 98.6)
Other South Asian	10	20	36533	73.1 (40.7, 131.3)	71.6 (39.3, 130.4)	71.2 (39.5, 128.1)	71 (38.9, 129.6)
African origin	10	15	49766.5	99.1 (60.7, 162.1)	97.4 (59, 160.9)	98.4 (60.4, 160.4)	98.2 (59.7, 161.6)
Chinese	25	55	43452	84.5 (62.5, 114.3)	82.5 (59.5, 114.4)	85.5 (63.2, 115.8)	85.3 (61.4, 118.5)

170120

For peer review only

Supplementary Materials Tables for Campbell et al Ethic and religious variations in participation in bowel screening in Scotland

Supplementary Material Table 7B. Age adjusted rates and risk ratios (RRs) for pathology detected (polyps, adenoma, cancer) (Rounds 1 & 2) by ethnic group in women. Results exclude Grampian health board. RRs are adjusted for age, UK/RoI-born (versus born outside UK/RoI) and socio-economic status (household tenure and combined individual and household level education) with 95% confidence intervals (CIs).

				RRs (95% CIs): adjustment			
**Ethnic group**	**Pathology detected**	**Positive screen test results**	**Rates**	**Age/ 100,000**	**Age and UK/RoI-born**	**Age and 2 socio-economic variables**	**Age, UK/RoI-born and 2 socio-economic variables**
WOMEN							
White Scottish	5275	14650	36006.8	100	100	100	100
Other White British	340	880	38451.2	107 (98.2, 116.7)	107 (98.1, 116.7)	103.4 (94.8, 112.8)	103.4 (94.8, 112.9)
White Irish	70	165	40946.5	111.9 (93, 134.5)	111.8 (93, 134.5)	111.9 (93.2, 134.4)	111.9 (93.2, 134.4)
Other White	50	150	32755.6	92.2 (73.3, 115.8)	91.5 (69.9, 119.7)	89.9 (71.6, 112.9)	90.2 (69.1, 117.9)
Any Mixed Background	10	20	30072.4	85 (44, 164.2)	84.7 (43.8, 163.9)	86.4 (44, 169.9)	86.6 (44, 170.4)
Indian	10	20	41942.6	126.4 (74.2, 215.1)	125.1 (71.4, 219.2)	124 (73, 210.3)	124.6 (71.3, 217.7)
Pakistani	.	35					
Other South Asian	.	.					
African origin	.	10					
Chinese	15	45	28605.7	83.9 (52.9, 132.9)	82.9 (50.1, 137.4)	82.3 (52, 130.5)	82.8 (50.1, 137)

170120

For peer review only - http://bmjopen.bmj.com/site/about/guidelines.xhtml

Supplementary Materials - Campbell et al Ethic and religious variations in bowel cancer screening in Scotland

Supplementary Material Figure 1

Directed Acrylic Graph (DAG) of factors influencing ethnicity and bowel screening uptake

A Directed Acrylic Graph (DAG) was developed, based on evidence form the literature, and discussion of study results. Initially 26 variables were identified as potential influencing factors to bowel screening uptake and a cross-matrix via expert opinion about the direction of influence between each of these variables was developed and tested for the causal effect identification in DAGitty v2.3 (http://dagitty.net/dags.html).

However, the initial model didn't allow estimation of the direct effect, and most of these variables were unobserved (latent) variables. The model was then stepwise reduced until a model was identified which allowed the estimation of the direct effecs. The figure shows the final model for the minimal sufficient adjustment set for estimating the direct effect of ethnicity on bowel screeming uptake, which included the variables age, country of birth, education, houshold tenure, religion, socioeconomic status (SES), and sex. This model informed the interpretation of our results and highlights areas for further investigation.

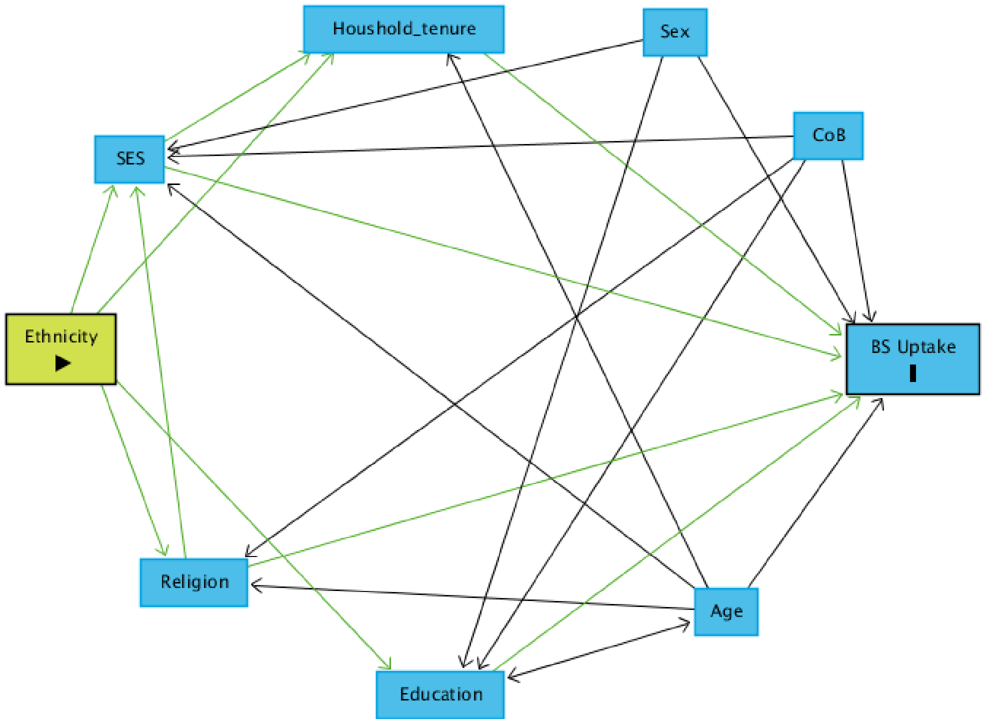

Initial factor set of 26 identified possible variables (observed variables in bold):

- **Ethnicity (Exposure)**
- **Bowel screening uptake (Outcome)**
- **Age**
- **Sex**
- Language
- Knowledge of screening
- Knowledge of cancer
- Previous experience of screening
- Health literacy
- **Education**
- Fatalism
- Perceptions of risk
- Fear of cancer
- Other priorities
- Self-efficacy
- Family history of cancer /illness
- **Country of birth (CoB)**
- **Socio-economic status**
- Marital status/ partnership
- **Religion**
- Cultural - including social norms
- Acculturation
- Frailty / co-morbidity
- **Rurality**
- Clinical support
- access to services

For peer review only

STROBE Statement—Checklist of items that should be included in reports of ***cohort studies***

	Item No	Recommendation	Page No
**Title and abstract**	1	(*a*) Indicate the study's design with a commonly used term in the title or the abstract	1
		(*b*) Provide in the abstract an informative and balanced summary of what was done and what was found	3
**Introduction**			
Background/rationale	2	Explain the scientific background and rationale for the investigation being reported	5
Objectives	3	State specific objectives, including any prespecified hypotheses	6
**Methods**			
Study design	4	Present key elements of study design early in the paper	7
Setting	5	Describe the setting, locations, and relevant dates, including periods of recruitment, exposure, follow-up, and data collection	7-9
Participants	6	(*a*) Give the eligibility criteria, and the sources and methods of selection of participants. Describe methods of follow-up<br>(*b*) For matched studies, give matching criteria and number of exposed and unexposed	7-8
Variables	7	Clearly define all outcomes, exposures, predictors, potential confounders, and effect modifiers. Give diagnostic criteria, if applicable	8
Data sources/ measurement	8*	For each variable of interest, give sources of data and details of methods of assessment (measurement). Describe comparability of assessment methods if there is more than one group	7/8
Bias	9	Describe any efforts to address potential sources of bias	7
Study size	10	Explain how the study size was arrived at	7
Quantitative variables	11	Explain how quantitative variables were handled in the analyses. If applicable, describe which groupings were chosen and why	8
Statistical methods	12	(*a*) Describe all statistical methods, including those used to control for confounding<br>(*b*) Describe any methods used to examine subgroups and interactions<br>(*c*) Explain how missing data were addressed<br>(*d*) If applicable, explain how loss to follow-up was addressed<br>(*e*) Describe any sensitivity analyses	8,9
**Results**			
Participants	13*	(a) Report numbers of individuals at each stage of study—eg numbers potentially eligible, examined for eligibility, confirmed eligible, included in the study, completing follow-up, and analysed<br>(b) Give reasons for non-participation at each stage<br>(c) Consider use of a flow diagram	10
Descriptive data	14*	(a) Give characteristics of study participants (eg demographic, clinical, social) and information on exposures and potential confounders<br>(b) Indicate number of participants with missing data for each variable of interest<br>(c) Summarise follow-up time (eg, average and total amount)	10
Outcome data	15*	Report numbers of outcome events or summary measures over time	10

For peer review only - http://bmjopen.bmj.com/site/about/guidelines.xhtml

Main results	16	(*a*) Give unadjusted estimates and, if applicable, confounder-adjusted estimates and their precision (eg, 95% confidence interval). Make clear which confounders were adjusted for and why they were included<br>(*b*) Report category boundaries when continuous variables were categorized<br>(*c*) If relevant, consider translating estimates of relative risk into absolute risk for a meaningful time period	10-16
Other analyses	17	Report other analyses done—eg analyses of subgroups and interactions, and sensitivity analyses	8
**Discussion**			
Key results	18	Summarise key results with reference to study objectives	19
Limitations	19	Discuss limitations of the study, taking into account sources of potential bias or imprecision. Discuss both direction and magnitude of any potential bias	19-20
Interpretation	20	Give a cautious overall interpretation of results considering objectives, limitations, multiplicity of analyses, results from similar studies, and other relevant evidence	20-21
Generalisability	21	Discuss the generalisability (external validity) of the study results	21
**Other information**			
Funding	22	Give the source of funding and the role of the funders for the present study and, if applicable, for the original study on which the present article is based	22

*Give information separately for exposed and unexposed groups.

Note: An Explanation and Elaboration article discusses each checklist item and gives methodological background and published examples of transparent reporting. The STROBE checklist is best used in conjunction with this article (freely available on the Web sites of PLoS Medicine at http://www.plosmedicine.org/, Annals of Internal Medicine at http://www.annals.org/, and Epidemiology at http://www.epidem.com/). Information on the STROBE Initiative is available at http://www.strobe-statement.org.

For peer review only

For peer review only - http://bmjopen.bmj.com/site/about/guidelines.xhtml

BMJ Open

Are there ethnic and religious variations in uptake of bowel cancer screening? A retrospective cohort study among 1.7 million people in Scotland

Journal:	*BMJ Open*
Manuscript ID	bmjopen-2020-037011.R1
Article Type:	Original research
Date Submitted by the Author:	01-Jul-2020
Complete List of Authors:	Campbell, Christine; The University of Edinburgh, Usher Institute<br>Douglas, Anne; The University of Edinburgh, Centre for Population Health Sciences<br>Williams, Linda; The University of Edinburgh, Usher Institute<br>Cezard, Genevieve; University of St Andrews<br>Brewster, David; The University of Edinburgh, Centre for Population Heath Sciences<br>Buchanan, Duncan; ISD Scotland<br>Robb, Kathryn; University of Glasgow<br>Stanners, Greig; NHS Scotland<br>Weller, David; The University of Edinburgh, General Practice<br>Steele, Robert; University of Dundee, Surgery and Molecular Oncology<br>Steiner, Markus; University of Aberdeen<br>Bhopal, Raj; The University of Edinburgh, Centre for Population Health Sciences
**Primary Subject Heading**:	Public health
Secondary Subject Heading:	Epidemiology, Oncology
Keywords:	Gastrointestinal tumours < ONCOLOGY, PUBLIC HEALTH, Health policy < HEALTH SERVICES ADMINISTRATION & MANAGEMENT, PREVENTIVE MEDICINE

SCHOLARONE™ Manuscripts

I, the Submitting Author has the right to grant and does grant on behalf of all authors of the Work (as defined in the below author licence), an exclusive licence and/or a non-exclusive licence for contributions from authors who are: i) UK Crown employees; ii) where BMJ has agreed a CC-BY licence shall apply, and/or iii) in accordance with the terms applicable for US Federal Government officers or employees acting as part of their official duties; on a worldwide, perpetual, irrevocable, royalty-free basis to BMJ Publishing Group Ltd ("BMJ") its licensees and where the relevant Journal is co-owned by BMJ to the co-owners of the Journal, to publish the Work in this journal and any other BMJ products and to exploit all rights, as set out in our licence.

The Submitting Author accepts and understands that any supply made under these terms is made by BMJ to the Submitting Author unless you are acting as an employee on behalf of your employer or a postgraduate student of an affiliated institution which is paying any applicable article publishing charge ("APC") for Open Access articles. Where the Submitting Author wishes to make the Work available on an Open Access basis (and intends to pay the relevant APC), the terms of reuse of such Open Access shall be governed by a Creative Commons licence – details of these licences and which Creative Commons licence will apply to this Work are set out in our licence referred to above.

Other than as permitted in any relevant BMJ Author's Self Archiving Policies, I confirm this Work has not been accepted for publication elsewhere, is not being considered for publication elsewhere and does not duplicate material already published. I confirm all authors consent to publication of this Work and authorise the granting of this licence.

For peer review only

Are there ethnic and religious variations in uptake of bowel cancer screening? A retrospective cohort study among 1.7 million people in Scotland

Running title: Ethnic variation in bowel screening uptake

Christine Campbell[1], Anne Douglas[1], Linda Williams[1], Geneviève Cezard[2], David H Brewster[3], Duncan Buchanan[4], Kathryn A. Robb[5], Greig Stanners[6], David Weller[1], Robert Steele[7], Markus Steiner[8], Raj Bhopal[1]

[1] Usher Institute, University of Edinburgh, Edinburgh, EH8 9DX, Scotland, UK

[2] Population and Health research group, School of Geography and Sustainable Development, University of St Andrews, Irvine building, St Andrews, KY16 9AL, Scotland, UK

[3]Scottish Cancer Registry, Information Services Division, Public Health & Intelligence, NHS National Services Scotland, Gyle Square, Edinburgh, EH12 9EB, Scotland, UK

[4] Information Services, NHS National Services Scotland, Gyle Square, Edinburgh, EH12 9EB, Scotland, UK

[5] Institute of Health & Wellbeing, University of Glasgow, Gartnavel Royal Hospital, 1055 Great Western Road, Glasgow G12 0XH, Scotland, UK

[6] Population Health Team, Information Services Division, NHS National Services Scotland, Meridian Court, Glasgow G2 6QE, Scotland, UK

[7] Centre for Research into Cancer Prevention and Screening (CRiPS), Medical Research Institute, Ninewells Hospital and Medical School, Dundee, DD1 9SY, Scotland, UK

[8]School of Medicine, University of Aberdeen, Aberdeen, AB25 2ZG, Scotland, UK

Corresponding Author:

For peer review only - http://bmjopen.bmj.com/site/about/guidelines.xhtml

Dr Christine Campbell, Usher Institute, University of Edinburgh, Teviot Place, Edinburgh, EH8 9DX, Scotland, UK; tel: +44 (0)131 650 9252; Christine.Campbell@ed.ac.uk

Key terms: colorectal screening, ethnic minority, religion, uptake

Words = 4,007

For peer review only

ABSTRACT

Objective

Cancer screening should be equitably accessed by all populations. Uptake of colorectal cancer screening was examined using the Scottish Health and Ethnicity Linkage Study that links the Scottish Census 2001 to health data by individual-level self-reported ethnicity and religion.

Setting

Data on 1.7 million individuals in two rounds of the Scottish Bowel Cancer Screening Programme (2007- 2013) were linked to the 2001 Census using the Scottish Community Health Index number.

Main outcome measure

Uptake of colorectal cancer screening, reported as age-adjusted risk ratios (RRs) by ethnic group and religion were calculated for men and women with 95% confidence intervals (CI).

Results

In the first, incidence screening round, compared to White Scottish men, Other White British (RR 109.6, 95% CI 108.8, 110.3) and Chinese (107.2, CI 102.8, 111.8) men had higher uptake. In contrast, men of all South Asian groups had lower uptake (Indian RR 80.5, CI 76.1, 85.1; Pakistani RR 65.9, CI 62.7, 69.3; Bangladeshi RR 76.6, CI 63.9, 91.9; Other South Asian RR 88.6, CI 81.8, 96.1). Comparable patterns were seen among women in all ethnic groups, e.g. Pakistani (RR 55.5, CI 52.5, 58.8). Variation in uptake was also observed by religion, with lower rates among Hindu (RR [95%CI]: 78.4 [71.8; 85.6]), Muslim (69.5 [66.7; 72.3]), and Sikh (73.4 [67.1; 80.3]) men compared to the reference population (Church of Scotland), with similar variation among women: lower rates were also seen among those who reported being Jewish, Roman Catholic, or with no religion.

For peer review only - http://bmjopen.bmj.com/site/about/guidelines.xhtml

Conclusions

There are important variations in uptake of bowel cancer screening by ethnic group and religion in Scotland, for both sexes that require further research and targeted interventions.

Trial registration: n/a

Strengths and limitations of this study

- The most fine-grained analyses of bowel screening uptake by ethnicity reported to date, with a nationally tested classification of ethnic groups
- The study benefits from high overall linkage rates of census and NHS CHI numbers, with a large national population, a high linkage rate with the Bowel Screening data
- The small numbers of outcomes for some non-White populations has required aggregation of data for some ethnic groups, restricting reporting of invasive cancer for some ethnic groups due potentially disclosive numbers
- Patterns of immigration to Scotland over the last 18 years have changed, in particular among those from Eastern Europe, and we do not report on bowel screening uptake among these populations
- The reported screening uptake rates are descriptive and not explanatory: although we adjusted for determinants of ethnic inequalities in bowel screening such as SES and UK-birth but these made little difference to the patterns observed, and further potential mechanisms need to be explored

For peer review only - http://bmjopen.bmj.com/site/about/guidelines.xhtml

BACKGROUND

Bowel cancer is the third most commonly diagnosed cancer in both men and women in Scotland.[1] Bowel cancer screening using the faecal occult blood test (FOBt) was started across all NHS boards (health authorities) in Scotland between June 2007 and December 2009, with those aged between 50- 74 years and registered with a general practice invited to participate every two years.[2] Routine use of a faecal immunochemical test (FIT) was introduced in November 2017. Although progress has been made, substantial variation in uptake is still observed by deprivation,[3] however, variation by ethnicity in Scotland has not been studied.

There is growing recognition of the challenges to minimising inequalities in cancer outcomes in minority ethnic populations across the UK. Recent work has demonstrated lower awareness of the breast and cervical screening programmes compared to White survey participants and very low (less than 30% of respondents) awareness of bowel screening overall.[4] Lower attendance among Asian invitees in the UK Flexible Sigmoidoscopy Trial has also been reported.[5] The reasons for these differences are likely to include the approach of services, cultural beliefs and attitudes, and health communication and literacy barriers.[6,7]

Reporting of inequalities in uptake of cancer screening by minority ethnic group has been limited by a failure in most health systems to routinely code ethnicity accurately. As a consequence, our understanding is based on area-based measures,[8,9] responders to surveys including items on ethnicity,[5] or name recognition software (e.g. Nam Pehchan).[10] Existing evidence is further limited by the use of very broad categories for ethnicity e.g. Indian subcontinent;[8] White/Black/Asian;[5] Hindu-Gujerati/Hindu-Other/Muslim/Sikh/Other Asian.[10] Within these constraints, variation in uptake has been observed internationally,[11] in the FOBt bowel screening pilots in England,[10,12,13] and has been reported in the English Screening Programme.[14] UK bowel screening databases (including Scotland) do not routinely include an ethnic code[15] so reported estimated uptake rates by ethnicity are based on area-level

For peer review only

characteristics rather than individual level data. Findings from other parts of the UK may not be generalisable due to differences in composition of ethnic minority groups and religious affiliations, cultural background and service provision. A better understanding of both screening uptake and screening outcomes, analysed by minority ethnic groups and by religion, has the potential to inform more targeted education and informed choice strategies (while recognizing that ethnicity, religion and cultural background are over-lapping although not synonymous identities).

This study made use of a unique UK resource, the Scottish Health and Ethnicity Linkage Study (SHELS): linkage of the 2001 Census in Scotland (with individual level self-reported ethnicity, country of birth, religion and a range of socio-demographic characteristics), with the Community Health Index (CHI) register number and through that to other health databases.[16,17] Linkage to the breast screening programme dataset enabled SHELS[18] to demonstrate lower uptake of breast screening among minority ethnic women in Scotland, even when adjusted for several confounding factors. The primary aim of this paper was to describe bowel cancer screening uptake rates in detail by self-reported ethnic group, including White Scottish, Other White British, White Irish, Other White, Indian, Pakistani, Bangladeshi, Other South Asian, Caribbean, African Other Black, Chinese, in addition to self-reported religious affiliation. Further, as previous SHELS linkage has shown lower directly age-standardised rates and ratios of colorectal cancer in the South Asian population in Scotland (especially in Pakistani men), as well as in Chinese men[19], linkage of census data with cancer registry has allowed us to examine test positivity, pathology and cancer outcomes by ethnic groups where available.

For peer review only

METHODS

Data linkage

Methods of SHELS retrospective cohort studies have been published.[16,17] We followed a strict protocol that preserved anonymity and maintained separation of personal data from the Census and National Health Service (NHS), and clinical data (Figure 1). SHELS used computerised, probability matching of names, addresses, sex and dates of birth to link the Census 2001 for Scotland, to the CHI, which is a register of patients using the NHS. This created a file containing the linked encrypted CHI and encrypted census numbers for a cohort of 4.62 million people (95% of those completing the census and 90% of the estimated Scottish population in 2001). We used this file to link census variables to a previously linked Scottish Bowel Cancer Screening Programme (SBCSP) and Scottish Cancer Registry (SMR06) database.

Ethnicity and religious data

The Scottish Census 2001 provided ethnic group as reported by either individuals or the householder completing the form based on a question followed by a choice of 14 categories. Unless stated otherwise we have used the official categories, capitalising them as in census reports. Ethnic group is a legally required field that was well completed (95.8%) and, after imputation (4.3%), available for 100% of those completing the census form.[20] If necessary because of small numbers, we aggregated the Bangladeshi group with the Other South Asian group; and the Caribbean, African, and Black Scottish or other Black groups into one African origin group in order to comply with data release stipulations of the data controller.. Any Mixed Background is one of the distinct ethnic categories in the Census, designed for use by people who perceive themselves as belonging to more than one ethnic group, usually with each parent in a different ethnic group Following our analytical strategy, ethnic groups were only omitted to avoid potential disclosure of identity. We did not report results for the 'All other' ethnic group as this is an exceptionally diverse group of people and it is difficult to interpret

For peer review only - http://bmjopen.bmj.com/site/about/guidelines.xhtml

results in any meaningful way. Religion was recorded on the Scottish Census 2001 in specific categories based on both self-reported current religion and self-reported religion of upbringing.

Screening uptake

Individuals aged 50-74 years are invited to participate in bowel screening in Scotland every two years (a screening 'round'). Analyses were restricted by age to 50-74 years as the age range invited to participate in the screening programme, but we also examined screening uptake in the over 75s who chose to 'opt-in'.[2] Uptake of bowel screening was defined as a completed screening round using the FOBt (i.e. screenee received a positive or negative test result).

Socio-demographic data

Census data included age, sex, country of birth (UK/Republic of Ireland (RoI) born or born outside UK/RoI) and socio-economic status. Four socio-economic indicators were used: (1) the postcode-based Scottish Index of Multiple Deprivation (SIMD), (2) highest qualification of the individual, (3) a combined measure of highest qualification (individual level for people aged 16-74 and household level for children and elderly, as individual data are not collected for these groups) and (4) household tenure.

99% of the White Scottish group, 50% of the Indian group, 59% of the Pakistani group, 42% of the Other South Asian group, 41% of the African origin group, 36% of the Chinese group and 28% of the Other White group were born in the UK/RoI in our linked census database.

Outcomes

We primarily analysed uptake (persons successfully completing a kit and getting a final result i.e. an outright positive or negative result) of bowel cancer screening between 2007 and 2013 in Scotland. First and second round (i.e. where eligible participants are invited every two years) of screening were analysed separately. We further analysed the rate of positive screening test

For peer review only - http://bmjopen.bmj.com/site/about/guidelines.xhtml

results in this participating population, and bowel cancer detection rates. The cohort of screening invitees analysed were those included in the Scottish Census 2001 who subsequently were still living in Scotland at the time of screening invitation. For analyses of screen detected invasive cancer, Round 1 and Round 2(i.e. where eligible participants are invited every two years) data were combined: Round 2 figures include many of the same people as in Round 1 results plus some newly entering the eligible age group, and who were resident at the 2001 Census.

For peer review only

Data analysis

We followed a pre-specified analysis plan (https://www.ed.ac.uk/usher/scottish-health-ethnicity-linkage/key-information). We calculated, for each outcome, by sex and ethnic group: uptake in screening in both Round 1 and Round 2; age-adjusted rates per 100,000 population; risk ratios (RRs) and their 95% confidence intervals (CI) using Poisson regression with robust variance adjusted for age and subsequently adjusted for socio-economic status and country of birth. We multiplied the estimates by 100 to facilitate the interpretation of the results as percentages, as per the SHELS policy and analysis plan. We adopted a previously published approach for choosing variables that were potential confounding showing consistency across ethnic groups.[16] Two socioeconomic status (SES) indicators (household tenure and combined qualification) were consistently associated with the outcome across ethnic groups. The standard reference population was the White Scottish population. We also compared uptake rates by religion separately for men and women.

Data were analysed using SAS V.9.4 (SAS Institute Inc, Cary, North Carolina, USA).

Limited availability of Grampian data

For technical reasons, data on pathology (polyps, adenoma, cancer) and invasive cancer were unavailable from Grampian Health Board.

Ethics and disclosure

The work was approved by the Multicentre Research Ethics Committee for Scotland and the Privacy Advisory Committee of NHS National Services Scotland. Caldicott Guardian approval was obtained for access to the SBCSP data. The ethical and other permissions and related issues have been reported in detail including an independent assessment of SHELS' approach by an ethicist.[16,17,21] To comply with the Data Protection Act and safe-setting rules the data set only contained specific disease outcomes. Other outcomes were excluded to minimise risks of inadvertent disclosure of identity. The analysis was conducted on a standalone computer in a locked room in the National Records of Scotland (NRS), by named researchers with appropriate clearance and training (LW, GC, and MS) and following a strict disclosure protocol.

Outputs leaving the safe setting as well as this manuscript were reviewed by the NRS Disclosure Committee. The analysis was done on exact numbers. However, the released numerators and denominators were rounded to the nearest 5.

Authors developed a Directed Acyclic Graph (DAG) to aid the interpretation of results and help generate areas for further investigation (Supplementary Materials Figure 1).

Patient and Public Involvement

SHELS established a Public Engagement Panel, comprised of a mix of ethnic groups, sexes and ages. This Public Engagement Panel provided PPI perspectives on SHELS methodological approach, including the research questions and design of this study. At the end of the study, results were shared with the Panel who commented on the findings and contributed to the dissemination plan.

For peer review only - http://bmjopen.bmj.com/site/about/guidelines.xhtml

RESULTS

Linkage

Linked data were available for 1,666,575 of 1,926,060 individuals invited to participate in Round 1 screening, a linkage rate of 86.5%. Of the 1.67 million matched at Round 1, 1,407,835 individuals were invited to Round 2. We present here Round 1 results, with Round 2 results and additional analyses available in Supplementary Materials (Supplementary Tables 1A, 1B, 2A, 2B, 3A, 3B, 4A, 4B, 5A, 5B, 6A, 6B, 6C, 6D, 7A, 7B).

Uptake of bowel screening by ethnic group

Uptake in specific ethnic groups were compared to the White Scottish population, unless specified otherwise. Figure 2 shows bowel cancer screening uptake in men and women for round 1 by ethnic group. For men, age-adjusted RRs were higher in the Other White British (RR [95%CI]: 109.6 [108.8; 110.3]) and Chinese (107.2 [102.8; 111.8]) groups as they were more likely to return their kit once invited to screening compared to White Scottish men. Uptake was comparatively lower in other ethnic groups and especially so in Indian (80.5 [76.1; 85.1]), Pakistani (65.9 [62.7, 69.3]), Bangladeshi (76.6 [63.9; 91.9]) and Other South Asian (88.6 [81.8; 96.1]) men. Further adjustment for UK/RoI-birth and socio-economic status did not greatly alter the associations apart from adjustment for UK/RoI-birth in Chinese men making their uptake converge towards the levels of uptake of White Scottish men (Supplementary Materials Table 1A).

Similarly in women (Figure 2), age-adjusted RRs were higher in both Other White British (110.9 [110.2; 111.6]) and Chinese (112 [108.2; 115.9) women compared to White Scottish women, and again uptake was comparatively lower in women from Indian (76.1 [72; 80.5]), Pakistani (55.5 [52.5; 58.8]), Bangladeshi 58.5 [45.6; 75.1]) and Other South Asian (79.3 [72; 107.2]) ethnic groups. Further adjustment for UK-birth and socio-economic status did not alter the associations observed (Supplementary Materials Table 1B).

For peer review only - http://bmjopen.bmj.com/site/about/guidelines.xhtml

Screening uptake rates by ethnic group for Round 2 showed similar patterns for both men and women.

Uptake of bowel screening by current religion and religion of upbringing

Figure 3 shows bowel cancer screening uptake in men and women for round 1 by self-reported current religion as in the 2001 Census. Age-adjusted RRs were lower among Hindu (RR [95%CI]: 78.4 [71.8; 85.6]), Muslim (69.5 [66.7; 72.3]0, and Sikh (73.4 [67.1; 80.3]) men compared to those who identified current religion as Church of Scotland. Smaller differences compared to the reference population were observed among those who reported being Jewish (87.3 [81.8, 93.2]), Roman Catholic (91.4 [90.8, 92]), or None (no religion) (97.7 [97.2, 98.2]). Further adjustment for UK/RoI-birth and socio-economic status did not alter the trends observed.

In women, there was generally a lower uptake across groups compared to those who identified current religion as Church of Scotland apart from the Other Christian, with age-adjusted RRs being lower among Hindu (73.2 [67; 80]), Muslim (57.8 [55.2; 60.5]), and Sikh (73.2 [67.4; 79.5]) women compared to the reference Church of Scotland population. Age-adjusted RRs for Roman Catholic women were lower (87.9 [87.4; 88.4]) compared to the reference Church of Scotland populations; further adjustment for UK-birth and socio-economic status only modestly reduced the differences.

Screening uptake rates by religion of upbringing for men (Supplementary Materials Table 2A) and women (Supplementary Materials Table 2B) showed overall similar patterns.

Screening uptake in Round 2 for both current religion and religion of upbringing showed similar patterns.

Positivity of bowel screening test by ethnic group

For peer review only - http://bmjopen.bmj.com/site/about/guidelines.xhtml

Table 1A shows that age-adjusted RRs for positivity of FOBt were lower in Other White British (60.5 [56.1; 65.3]) men compared to White Scottish men in round 1. For women, positivity by ethnic group in round 1 (Table 1B) also showed lower positivity for the other White British group (67.3 [61.6; 73.5]) compared to White Scottish women. However for selected other ethnic groups including Indian and Pakistani women there is some indication of lower test positivity rates compared to White Scottish women but CIs were wide due to the small sample size and straddling the reference value of 100. Similar patterns were seen in Round 2 for both men and women.

Bowel cancer detection, and pathology by ethnic group

Table 2 shows bowel cancer detection rates via the screening test by ethnic group, for rounds 1 and 2, and for men and women combined, as this was necessary given the small numbers. Compared to the White Scottish population, Other White British individuals had a lower age-adjusted risk ratio of a diagnosis of screen-detected invasive cancer (84 [71; 99.3]); this result was not greatly altered after adjustment for UK-born and socio-economic status. Over the two rounds of screening, the number of invasive cancers found in individuals from other ethnic groups were too small to report for the risk of disclosure.

Supplementary Materials Tables 3A and 3B show age-adjusted rates and risk ratios for pathology detected, for polyps, adenomas and cancer combined, for men and women respectively. In comparison to the White Scottish population, numbers were small in each of the other ethnic groups. Only for Pakistani men was a lower rate of pathology detected (64.5 [42.5; 97.7]) compared to White Scottish men.

Uptake of bowel screening in older individuals

Individuals aged 75 and older are able to opt-in to bowel screening in Scotland. Table 3A and 3B show age-adjusted risk ratios for screening uptake by ethnic group for men and women

For peer review only - http://bmjopen.bmj.com/site/about/guidelines.xhtml

respectively. Chinese men had higher uptake (112.8 [113.3; 114.3]) compared to White Scottish men (Table 3A), as did Chinese women compared to White Scottish women (Table 3B: 116.7 [115; 118.5]). Adjustment for SES did not greatly affect this association in either Chinese men or women, however further adjustment for UK-birth in Chinese men the risk ratio converged towards that of White Scottish men.

For peer review only

For peer review only - http://bmjopen.bmj.com/site/about/guidelines.xhtml

DISCUSSION

Summary of findings

Although ethnic variation in colorectal screening uptake is increasingly recognised internationally,[22] detailed description in relation to specific ethnic groups is lacking. We report complex patterns of variation in colorectal cancer screening uptake by ethnic group in Scotland, with pronounced lower screening uptake among the South Asian groups compared to the White Scottish population, and higher uptake among the Chinese and Other White British populations. We found little variation by ethnicity in later stages of the screening process.

Strengths and Limitations of the study

Our results are to our knowledge the most fine-grained analyses of bowel screening uptake by ethnicity reported to date, and with a nationally tested classification of ethnic groups. For the first time, national Scottish Census 2001 data were used to show differences in uptake for separate Indian, Pakistani and Bangladeshi groups, for separate White groups, and for the first time showing uptake among Caribbean, African, and Chinese groups as well as by religious groups. Additionally, SHELS benefits from high overall linkage rates of census and NHS CHI numbers (95%), with a large national population (4.62 million people), and in this study a high linkage rate with the Bowel Screening data (86%). However, we acknowledge that the small numbers of outcomes for some non-white populations has required aggregation of heterogeneous ethnic groups; e.g. African, Caribbean, Black, Black Scottish or Black British. For invasive cancers we were unable to report on some ethnic groups due to reporting restrictions on potentially disclosive numbers (Table 3). Given the constraints of data release for reasons of patient confidentiality, understanding patterns of uptake in some ethnic groups will require additional research in other settings where numbers within distinct ethnic groups are sufficiently large.

For peer review only - http://bmjopen.bmj.com/site/about/guidelines.xhtml

We are reporting on 2001 Census data. Immigration to Scotland over the last 18 years has affected the distribution of ethnic groups within Scotland:[23,24] in particular, we do not report on bowel screening uptake among the Polish population, now one of Scotland's largest ethnic groups, where breast screening uptake is low.[25] Such analyses are not possible routinely and require a new study with linkage of bowel screening data to the 2011 Census. Nonetheless, the results reported here provide important insights into recent uptake patterns and set a benchmark for any future variation in bowel screening uptake rates as the population profile changes.

Finally, we recognise that the reported screening uptake rates are descriptive and not explanatory. We adjusted for determinants of ethnic inequalities in bowel screening such as SES and UK-birth but these made little difference to the patterns observed. Further potential mechanisms need to be explored, including cultural and religious beliefs, and the influence (if any) of knowledge of or exposure to screening programmes in other health systems (see Supplementary Figure 1 for potential variables influencing participation).

Data on a number of variables (pathology (polyps, adenoma, cancer) and invasive cancers) were unavailable from Grampian Health Board: sensitivity analyses (available on request) indicates that approximately 10-12% of the denominator in the Scottish population were missing for these variables in the Scottish population. Grampian Health Board comprises only 10.1% of the Scottish population, and with a non-White Scottish population of 15% compared to 12% in Scotland overall, there is no reason to expect that inclusion of these data would have altered the observed patterns in Table 2[26]. Data on uptake rates were complete.

Existing literature

We found lower rates of screening uptake in South Asian populations, reflected in both ethnic group and current religion Lower screening uptake among South Asian communities in the UK has been a feature of the screening programme since its inception[8,9,12] and the factors

For peer review only

influencing this are increasingly being understood. Many factors such as lack of awareness and understanding of the purpose of screening, and fear and fatalism about cancer are seen across all ethnic groups.[6,27-30] However, limitations with English-language screening materials (translated materials often require request), the need to rely on younger family members, cultural difficulties associated with handling of faeces, and social norms are additional barriers among South Asian ethnic groups.[7,30] Differences in breast screening uptake by ethnic group in Scotland have been reported previously by our group (higher non-attendance rates to breast cancer screening among Pakistani, Black, Other South Asian and Indian women),[18] as has variation in relation to numerous other health outcomes.[31-33]

As mentioned, lower directly age-standardised rates and ratios of colorectal cancer in the South Asian population have been reported in Scotland.[19] The RRs we report here suggesting lower RRs of FOBt positivity in Indian and Pakistani men (Tables 1A and 1B) are consistent with this, although need to be interpreted with care due to wide confidence intervals. Lower colorectal cancer rates in some ethnic communities may result in less perceived personal relevance and hence tailored educational interventions will need to acknowledge lower colorectal cancer rates while also addressing the identified barriers and facilitators.[34-37] There is a need for open discussion within bowel screening programmes and policy making of potentially variable benefits for different ethnic groups of screening uptake. The lower uptake rates *may* be appropriate for some groups, and genuine informed consent may require acknowledgement that some have less to gain in terms of absolute risk reduction. At a programmatic level, there is a balance between lower cancer risk and uptake of screening, and further work is warranted to address how issues of equality of access, cost-effectiveness, and effectiveness are maintained. Although at a population level the risk may be lower, messages aimed at the individual level need to communicate clearly the potential advantages of screening uptake within an informed choice framework.

For peer review only - http://bmjopen.bmj.com/site/about/guidelines.xhtml

The relatively high uptake rates among both Chinese men and women compared to the White Scottish men and women were unexpected, and not previously recognised in the Scottish population. Bansal and colleagues found that age-adjusted RRs for breast screening uptake were similar among Chinese women compared to White Scottish women.[18] High FOBt positivity rates were observed in both Chinese men and women in both rounds 1 and 2; this is despite the lack of evidence of higher incidence of colorectal cancer in the Chinese community in earlier SHELS work.[19] Further research is warranted, not only to determine if these findings can be replicated in other Chinese communities in the UK, but also to explore any cultural or other factors underlying high screening uptake. Low awareness of colorectal cancer screening was found among Chinese participants in an EthniBus survey.[4] Importantly, though, as noted above low rates of colorectal screening uptake (by flexible sigmoidoscopy) have been reported in areas of high non-White ethnicity but these were not broken down by ethnic group.[38]

While numbers were relatively small, only limited variation in colorectal screening uptake was seen in the over 75 population; there is, however, some indication that South Asian men and women were less likely to opt-in.This is a self-selecting group of individuals who are likely to differ from their peers in terms of other health behaviours, motivation and levels of co-morbidity. The low overall number of opt-ins is consistent with findings from the Bowel Screening Pilot in England.[8]

Implications for policy and practice

Addressing observed inequalities in screening uptake will require multi-faceted interventions. Telephone-based interventions have been shown to increase colorectal screening uptake in ethnically-diverse areas of London[39] but have resource implications. Patient navigators have been shown to be effective in some settings.[40] Further exploratory work and engagement with local communities is needed to develop, refine, and test culturally-appropriate interventions with salience to different ethnic groups; critically, these must ensure principles of informed

For peer review only - http://bmjopen.bmj.com/site/about/guidelines.xhtml

choice are respected and incorporated throughout.[41] Our reported variations in uptake by religion are, seemingly, novel: in particular, the lower uptake among Roman Catholic populations compared to the reference population, persisting even when adjusted for socio-economic variables, is puzzling. Addressing such variation by religion may be amenable to targeted faith-based interventions.[42] Others have found variable influence of religiosity on screening uptake, with social support only partially mediating the relationship between religiosity and bowel screening uptake.[43] Comparing facilitators and barriers across groups may provide fresh insight into potential interventions.[44] Further, the introduction of the faecal immunochemical test (FIT) in the Scottish Bowel Screening Programme in late 2017 has been shown to increase overall screening uptake:[2] this provides an impetus to monitor the impact within ethnic groups over time (work currently underway by authors).

For peer review only - http://bmjopen.bmj.com/site/about/guidelines.xhtml

ADDITIONAL INFORMATION

Ethics approval and consent to participate

The work was approved by the Multicentre Research Ethics Committee for Scotland (16/12/2013, Scotland A REC Ref: 13/SS/0225) and the Privacy Advisory Committee of NHS National Services Scotland**.** The study was performed in accordance with the Declaration of Helsinki.

Availability of data and material

Researchers who wish to access SHELS data should apply to National Records of Scotland (https://www.nrscotland.gov.uk/) and ISD (http://www.isdscotland.org/). They are maintained in a secure environment and governed by ethical and other restrictions on access.

Conflict of interest

The authors declare no conflict of interest

Funding

This work was supported by the Chief Scientist's Office (grant number CZH/4/878), Cancer Research UK (grant number C3743/A16594), and supplementary funding from NHS Health Scotland. ISD and National Records of Scotland both made 'in-house' contributions to the work.

Authors' contributions

All authors meet authorship criteria. RB was the PI of SHELS, and CC was the PI of this specific component of the research. RB, CC, AD, DHB and DW conceived the study and planned it along with KAR, GS, and RS. LW, GC and MS carried out data analysis. DB provided statistical advice. All authors contributed to the writing of the paper. CC is the guarantor.

For peer review only - http://bmjopen.bmj.com/site/about/guidelines.xhtml

REFERENCES

1. Information Statistics Division (ISD) Scotland Cancer Statistics http://www.isdscotland.org/Health-Topics/Cancer/Cancer-Statistics/Colorectal/ *Accessed 23/08/19*

2. National Services Division NHS Scotland Bowel Cancer Screening Programme http://www.nsd.scot.nhs.uk/services/screening/bowelscreening/index.html *Accessed 23/08/19*

3. Quyn AJ, Fraser CG, Stanners G, Carey FA, Carden C, Shaukat A, et al. Uptake trends in the Scottish Bowel Screening Programme and the influences of age, sex, and deprivation. J Med Screen. 2018 Mar;25(1):24-31. doi: 10.1177/0969141317694065. Epub 2017 Mar 24.

4. Robb K, Wardle J, Stubbings S, Ramirez A, Austoker J, Macleod U, et al. Ethnic disparities in knowledge of cancer screening programmes in the UK. J Med Screen. 2010;17(3):125-31. doi: 10.1258/jms.2010.009112.

5. Robb KA, Power E, Atkin W, Wardle J. Ethnic differences in participation in flexible sigmoidoscopy screening in the UK. J Med Screen. 2008;15(3):130-6. doi: 10.1258/jms.2008.007112.

6. Austin KL, Power E, Solarin I, Atkin WS, Wardle J, Robb KA. Perceived barriers to flexible sigmoidoscopy screening for colorectal cancer among UK ethnic minority groups: a qualitative study. J Med Screen. 2009;16(4):174-9. doi: 10.1258/jms.2009.009080.

7. Lo SH, Waller J, Vrinten C, Kobayashi L, von Wagner C. Social Cognitive Mediators of Sociodemographic Differences in Colorectal Cancer Screening Uptake. Biomed Res Int. 2015;2015:165074. doi: 10.1155/2015/165074. Epub 2015 Oct 4.

8. Weller D, Coleman D, Robertson R, Butler P, Melia J, Campbell C, et al. The UK colorectal cancer screening pilot: results of the second round of screening in England. Br J Cancer. 2007 Dec 17;97(12):1601-5. Epub 2007 Nov 20.

9. von Wagner C, Baio G, Raine R, Snowball J, Morris S, Atkin W, et al. Inequalities in participation in an organized national colorectal cancer screening programme: results from

For peer review only - http://bmjopen.bmj.com/site/about/guidelines.xhtml

the first 2.6 million invitations in England. Int J Epidemiol. 2011 Jun;40(3):712-8. doi: 10.1093/ije/dyr008. Epub 2011 Feb 17.

10. Szczepura A, Johnson M, Orbell S, Gumber A, O'Sullivan I, Clay D, et al. Ethnicity: UK Colorectal Cancer Screening Pilot. 2003 https://webarchive.nationalarchives.gov.uk/20120511035604/http://www.cancerscreening.nhs.uk/bowel/ethnicity-finalreport.pdf *Accessed 23/08/19*

11. Lansdorp-Vogelaar I, Kuntz KM, Knudsen AB, van BM, Zauber AG, Jemal A. Contribution of screening and survival differences to racial disparities in colorectal cancer rates. Cancer Epidemiol Biomarkers Prev 2012 May;21(5):728-36

12. Price CL, Szczepura AK, Gumber AK, Patnick J. Comparison of breast and bowel cancer screening uptake patterns in a common cohort of South Asian women in England. BMC Health Serv Res. 2010 Apr 27;10:103.

13. Moss SM, Campbell C, Melia J, Coleman D, Smith S, Parker R, et al. Performance measures in three rounds of the English bowel cancer screening pilot. Gut. 2012 Jan;61(1):101-7. Epub 2011 May 10.

14. Logan RF, Patnick J, Nickerson C, Coleman L, Rutter MD, von Wagner C; on behalf of the English Bowel Cancer Screening Evaluation Committee. Outcomes of the Bowel Cancer Screening Programme (BCSP) in England after the first 1 million tests. Gut 2011 Dec 7. [Epub ahead of print]

15. Allaby M, Mulhall C, Lines C. Analyses of inequality in national screening programmes using existing data systems. A supplementary report for the UK National Screening Committee. Oxford: Public Health Resource Unit February 2009

16. Bhopal R, Fischbacher C, Povey C, Chalmers J, Mueller G, Steiner M, et al. Cohort profile: Scottish health and ethnicity linkage study of 4.65 million people exploring ethnic variations in disease in Scotland. Int J Epidemiol. 2011 Oct;40(5):1168-75. Epub 2010 Jul 24

17. Fischbacher CM, Bhopal R, Povey C, Steiner M, Chalmers J, Mueller G, et al. Record linked retrospective cohort study of 4.6 million people exploring ethnic variations in disease: myocardial infarction in South Asians. BMC Public Health. 2007 Jul 5;7:142.

For peer review only - http://bmjopen.bmj.com/site/about/guidelines.xhtml

18. Bansal N, Bhopal RS, Steiner MF, Brewster DH; Scottish Health and Ethnicity Linkage Study. Major ethnic group differences in breast cancer screening uptake in Scotland are not extinguished by adjustment for indices of geographical residence, area deprivation, long-term illness and education. Br J Cancer. 2012 Apr 10;106(8):1361-6. doi: 10.1038/bjc.2012.83. Epub 2012 Mar 13

19. Bhopal RS, Bansal N, Steiner M, Brewster DH; Scottish Health and Ethnicity Linkage Study. Does the 'Scottish effect' apply to all ethnic groups? All-cancer, lung, colorectal, breast and prostate cancer in the Scottish Health and Ethnicity Linkage Cohort Study. BMJ Open. 2012 Sep 25;2(5). pii: e001957. doi: 10.1136/bmjopen-2012-001957. Print 2012.

20. National Records of Scotland 2001 Census Information https://www.nrscotland.gov.uk/statistics-and-data/census/2001-census *Accessed 23/08/19*

21. Boyd KM. Ethnicity and the ethics of data linkage. BMC Public Health 2007;7:318

22. de Klerk CM, Gupta S, Dekker E, Essink-Bot ML; Expert Working Group 'Coalition to reduce inequities in colorectal cancer screening' of the World Endoscopy Organization. Socioeconomic and ethnic inequities within organised colorectal cancer screening programmes worldwide. Gut. 2018 Apr;67(4):679-687. doi: 10.1136/gutjnl-2016-313311. Epub 2017 Jan 10.

23. National Records of Scotland Census 2011: Population Estimates for Scotland https://www.nrscotland.gov.uk/news/2012/census-2011-population-estimates-for-scotland *Accessed 23/08/19*

24. Census 2011: Detailed characteristics on Ethnicity, Identity, Language and Religion in Scotland – Release 3A https://www.scotlandscensus.gov.uk/news/census-2011-detailed-characteristics-ethnicity-identity-language-and-religion-scotland-%E2%80%93 *Accessed 23/08/19*

For peer review only - http://bmjopen.bmj.com/site/about/guidelines.xhtml

25. Gorman DR, Porteous LA. Influences on Polish migrants' breast screening uptake in Lothian, Scotland. Public Health. 2018 May;158:86-92. doi: 10.1016/j.puhe.2017.11.026. Epub 2018 Feb 13

26. Scotland's Census 2001 Key Statistics for Council areas and Health Board areas Scotland. General Register Office for Scotland Edinburgh. February 2003. https://www.nrscotland.gov.uk/files/statistics/key_stats_chareas.pdf *Accessed 18/06/20*

27. Dharni N, Armstrong D, Chung-Faye G, Wright AJ. Factors influencing participation in colorectal cancer screening—a qualitative study in an ethnic and socio-economically diverse inner city population. Health Expectations. 2017 Aug;20(4):608-17

28. Vrinten C, Waller J, von Wagner C, Wardle J. Cancer fear: facilitator and deterrent to participation in colorectal cancer screening. Cancer Epidemiol Biomarkers Prev. 2015 Feb;24(2):400-5. doi: 10.1158/1055-9965.EPI-14-0967.

29. Taskila T, Wilson S, Damery S, Roalfe A, Redman V, Ismail T, et al. Factors affecting attitudes toward colorectal cancer screening in the primary care population. Br J Cancer. 2009 Jul 21;101(2):250-5. doi: 10.1038/sj.bjc.6605130. Epub 2009 Jun 23.

30. Palmer CK, Thomas MC, McGregor LM, von Wagner C, Raine R. Understanding low colorectal cancer screening uptake in South Asian faith communities in England--a qualitative study. BMC Public Health. 2015 Oct 1;15:998. doi: 10.1186/s12889-015-2334-9.

31. Cezard GI, Bhopal RS, Ward HJ, Bansal N, Bhala N; SHELS researchers. Ethnic variations in upper gastrointestinal hospitalizations and deaths: the Scottish Health and Ethnicity Linkage Study. Eur J Public Health. 2016 Apr;26(2):254-60. doi: 10.1093/eurpub/ckv182. Epub 2015 Oct 9.

32. Bhopal RS, Bansal N, Fischbacher C, Brown H, Capewell S; Scottish Health and Ethnicity Linkage Study (SHELS). Ethnic variations in chest pain and angina in men and women: Scottish Ethnicity and Health Linkage Study of 4.65 million people. Eur J Prev Cardiol. 2012 Dec;19(6):1250-7. doi: 10.1177/1741826711425775. Epub 2011 Oct 5

For peer review only - http://bmjopen.bmj.com/site/about/guidelines.xhtml

33. Bhopal RS, Cezard G, Bansal N, Ward HJ, Bhala N; SHELS researchers. Ethnic variations in five lower gastrointestinal diseases: Scottish health and ethnicity linkage study. BMJ Open. 2014 Oct 21;4(10):e006120. doi: 10.1136/bmjopen-2014-006120

34. Padmanabhan H, Widlak M, Nevill A, McKaig B, Brookes M, Veitch A. Ethnic variation in colorectal cancer risk following a positive faecal occult blood test in an English bowel cancer screening programme centre. Eur J Gastroenterol Hepatol. 2015 Nov;27(11):1281-5. doi: 10.1097/MEG.0000000000000443.

35. Trinh QD, Nguyen PL, Leow JJ, Dalela D, Chao GF, Mahal BA, et al. Cancer-specific mortality of Asian Americans diagnosed with cancer: a nationwide population-based assessment. J Natl Cancer Inst. 2015 Mar 20;107(6):djv054. doi: 10.1093/jnci/djv054. Print 2015 Jun.

36. Jerant A, To P, Franks P. The effects of tailoring knowledge acquisition on colorectal cancer screening self-efficacy. J Health Commun. 2015;20(6):697-709. doi: 10.1080/10810730.2015.1018562. Epub 2015 Apr 30.

37. Gaduputi V, Chandrala C, Tariq H, Sakam S, Dev A, Chilimuri S. Influence of perception of colorectal cancer risk and patient bowel preparation behaviors: a study in minority populations. Clin Exp Gastroenterol. 2015 Jan 28;8:69-75. doi: 10.2147/CEG.S75593. eCollection 2015.

38. McGregor LM, Bonello B, Kerrison RS, Nickerson C, Baio G, Berkman L, et al. Uptake of Bowel Scope (Flexible Sigmoidoscopy) Screening in the English National Programme: the first 14 months. J Med Screen. 2015 Sep 20. pii: 0969141315604659. [Epub ahead of print]

39. Shankleman J, Massat NJ, Khagram L, Ariyanayagam S, Garner A, Khatoon S, et al. Evaluation of a service intervention to improve awareness and uptake of bowel cancer screening in ethnically-diverse areas. Br J Cancer. 2014 Sep 23;111(7):1440-7. doi: 10.1038/bjc.2014.363. Epub 2014 Jul 1.

40. Devereux PG, Gray J, Robinson S, Galvin J, Gutierrez J. Using Community Engagement and Navigators to Increase Colon Cancer Screening and Patient Outcomes. Health Promot Pract. 2019 Jan;20(1):85-93. doi: 10.1177/1524839918757485. Epub 2018 Feb 9.

For peer review only - http://bmjopen.bmj.com/site/about/guidelines.xhtml

41. van Agt HM, Korfage IJ, Essink-Bot ML. Interventions to enhance informed choices among invitees of screening programmes-a systematic review. *Eur J Public Health*. 2014;24(5):789-801. doi:10.1093/eurpub/ckt205

42. Hou SI, Cao X. A Systematic Review of Promising Strategies of Faith-

Based Cancer Education and Lifestyle Interventions Among Racial/Ethnic Minority Groups. J Cancer Educ. 2018 Dec;33(6):1161-1175. doi: 10.1007/s13187-017-1277-5. Review

43. Leyva B, Nguyen AB, Allen JD, Taplin SH, Moser RP. Is Religiosity Associated with Cancer Screening? Results from a National Survey. J Relig Health. 2015 Jun;54(3):998-1013. doi: 10.1007/s10943-014-9843-1.

44. Davidson EM, Liu JJ, Bhopal R, White M, Johnson MR, Netto G, et al. Behavior change interventions to improve the health of racial and ethnic minority populations: a tool kit of adaptation approaches. Milbank Q. 2013 Dec;91(4):811-51. doi: 10.1111/1468-0009.12034. Review.

For peer review only

Table 1A. Positive screen test results (Round 1) by ethnic group in men: age adjusted rates and risk ratios (RRs). RRs are adjusted for age, UK/RoI-born (versus born outside UK/RoI) and socio-economic status (household tenure and combined individual and household level education) with 95% confidence intervals (CIs).

Ethnic group	Positive screen test results	Completed screen kit returned	Rates/ 100,000	RRs (95% CIs): adjustment<br>Age	Age and UK/RoI-born	Age and 2 socio-economic variables	Age, UK/RoI-born and 2 socio-economic variables
MEN							
White Scottish	11100	362865	3060	100	100	100	100
Other White British	685	37040	1844	60.5 (56.1, 65.3)	60.9 (56.4, 65.7)	68.9 (63.8, 74.5)	69.2 (64.1, 74.7)
White Irish	130	4220	3081	98.6 (83.2, 116.8)	98.7 (83.3, 116.9)	99.5 (84, 117.8)	99.5 (84, 117.9)
Other White	110	4045	2770	96 (80, 115.2)	111 (90.3, 136.5)	104.6 (87.2, 125.5)	115.3 (93.3, 142.5)
Any Mixed Background	10	310	2913	101.6 (53.1, 194.5)	111.8 (58.4, 213.9)	108.3 (56.8, 206.6)	114.7 (60.2, 218.7)
Indian	15	705	1844	58.7 (34.3, 100.5)	71.6 (41.1, 124.6)	67.6 (39.5, 115.7)	76.9 (44.1, 134)
Pakistani	20	1015	2172	74.8 (49.9, 112.3)	92 (59.7, 141.6)	74.5 (49.6, 111.7)	85.3 (55.3, 131.6)
Other South Asian	10	400	2764	105.2 (59.1, 187)	126.9 (70.4, 228.6)	113.2 (64, 200.2)	127.3 (71.2, 227.7)
African origin	10	360	2500	97.4 (51.1, 185.7)	114.7 (59.4, 221.2)	102.5 (53.8, 195.4)	113.7 (58.9, 219.4)
Chinese	30	990	3128	114.2 (80.9, 161.3)	141.1 (96.7, 205.7)	109.4 (77.4, 154.5)	125.7 (86, 183.7)

Table 1B. Positive screen test results (Round 1) by ethnic group in women: age adjusted rates and risk ratios (RRs). RRs are adjusted for age, UK/RoI-born (versus born outside UK/RoI) and socio-economic status (household tenure and combined individual and household level education) with 95% confidence intervals (CIs).

				RRs (95% CIs): adjustment			
**Ethnic group**	**Positive screen test results**	**Completed screen kit returned**	**Rates/ 100,000**	**Age**	**Age and UK/RoI-born**	**Age and 2 socio-economic variables**	**Age, UK/RoI-born and 2 socio-economic variables**
WOMEN							
White Scottish	8015	444425	1803	100	100	100	100
Other White British	510	42950	1187	67.3 (61.6, 73.5)	67.3 (61.5, 73.5)	76.4 (69.9, 83.5)	76.2 (69.7, 83.3)
White Irish	80	5255	1542	82.8 (66.7, 102.7)	82.8 (66.7, 102.7)	86.2 (69.5, 107)	86.2 (69.5, 107)
Other White	90	5840	1523	86.9 (70.7, 106.8)	86.8 (67.1, 112.3)	98.8 (80.3, 121.5)	92.8 (71.2, 120.8)
Any Mixed Background	10	435	2771	169.8 (96.7, 297.9)	169.6 (95.7, 300.7)	173.6 (99.4, 303.4)	167.5 (94.9, 295.4)
Indian	10	690	1744	100.8 (58.2, 174.5)	100.6 (57.1, 177.3)	110.5 (63.8, 191.4)	102.6 (58.1, 181.3)
Pakistani	15	870	1724	106.5 (64.6, 175.5)	106.2 (62.9, 179.3)	98.6 (59.8, 162.6)	91.2 (53.9, 154.5)
Other South Asian	.	265					
African origin	10	395	1515	98.2 (44.3, 217.6)	98.1 (44, 218.7)	107.2 (48.5, 236.9)	101.1 (45.4, 225.2)
Chinese	25	1210	2066	130.6 (88.9, 191.8)	130.3 (85, 199.9)	125.2 (85.4, 183.5)	115.2 (75, 177)

For peer review only

Table 2. Screen detected invasive cancer by ethnic group (Rounds 1 & 2; men & women combined): age adjusted rates and risk ratios (RRs). Results exclude Grampian health board. RRs are adjusted for sex, age, UK/RoI-born (versus born outside UK/RoI) and socio-economic status (household tenure and combined individual and household level education) with 95% confidence intervals (CIs).

				RRs (95% CIs): adjustment			
**Ethnic group**	**Cancers**	**Invited into screening**	**Rates/ 100,000**	**Age**	**Age and UK/RoI-born**	**Age and 2 socio-economic variables**	**Age, UK/RoI-born and 2 socio-economic variables**
White Scottish	2025	2428585	83.4	100	100	100	100
Other White British	145	205420	70.5	84 (71, 99.3)	85 (71.8, 100.5)	79.5 (67.1, 94.1)	80.4 (67.9, 95.2)
White Irish	25	29770	77.3	86.5 (57.4, 130.4)	86.6 (57.5, 130.5)	86.7 (57.5, 130.6)	86.7 (57.5, 130.7)
Other White *	20	28620	71.2	92.5 (59.6, 143.7)	130 (71.5, 236.4)	88.9 (57.2, 138)	127.1 (70.4, 229.7)

* Results for Any Mixed Background, South Asian (Indian, Pakistan or other), or Chinese ethnic groups are not provided as they are so few as to be potentially disclosive

(see Methods)

For peer review only - http://bmjopen.bmj.com/site/about/guidelines.xhtml

Table 3A. Bowel cancer screening uptake (Round 1) by ethnic group in men aged 75 years and over: age adjusted rates and risk ratios (RRs) RRs are adjusted for UK/RoI-born (versus born outside UK/RoI) and socio-economic status (household tenure and combined individual and household level education) with 95%

				RRs (95% CIs): adjustment			
**Ethnic group**	**Completed screen kit returned**	**Requested screening**	**Rates/ 100,000**	**Unadjusted**	**UK/RoI-born**	**2 socio-economic variables**	**UK/RoI-born and 2 socio-economic variables**
MEN							
White Scottish	2460	2780	88489	100	100	100	100
Other White British	180	215	84186	95 (89.5, 100.8)	94.7 (89.2, 100.5)	94.4 (88.8, 100.3)	94.2 (88.6, 100)
White Irish	30	30	87500	98.7 (86.6, 112.6)	98.5 (86.4, 112.3)	98.5 (86.3, 112.4)	98.3 (86.1, 112.2)
Other White	20	25	88000	99.3 (85.9, 114.8)	93.2 (78.9, 110.1)	98.2 (85, 113.4)	92.6 (78.6, 109.2)
Any Mixed Background	.	.					
South Asian	15	20	76190.5	86 (67.7, 109.2)	78.3 (60.8, 100.8)	85.1 (67.3, 107.7)	78.1 (60.8, 100.4)
Chinese	10	10	100000	112.8 (111.3, 114.3)	102.3 (93.6, 111.7)	115.1 (110.3, 120.1)	105.2 (95.3, 116)

For peer review only

Table 3B. Bowel cancer screening uptake (Round 1) by ethnic group in women aged 75 years and over: age adjusted rates and risk ratios (RRs). RRs are adjusted for UK/RoI-born (versus born outside UK/RoI) and socio-economic status (household tenure and combined individual and household level education) with 95% confidence intervals (CIs).

				RRs (95% CIs): adjustment			
**Ethnic group**	**Completed screen kit returned**	**Requested screening**	**Rates 100,000**	**Unadjusted**	**UK/RoI-born**	**2 socio-economic variables**	**UK/RoI-born and 2 socio-economic variables**
WOMEN							
White Scottish	2470	2885	85615.3	100	100	100	100
Other White British	170	195	87113.4	101.7 (96.1, 107.5)	101.6 (96.1, 107.5)	100.9 (95.3, 106.8)	100.9 (95.3, 106.8)
White Irish	30	30	90322.6	105.4 (93.9, 118.4)	105.4 (93.9, 118.4)	105.7 (94.1, 118.9)	105.7 (94.1, 118.9)
Other White	40	40	95000	110.9 (103.1, 119.2)	110.3 (99.7, 122.2)	110.3 (102.5, 118.7)	109.9 (99.2, 121.8)
Any Mixed Background	.	.					
South Asian	10	15	64705.9	75.5 (53.1, 107.3)	75.1 (52.2, 107.9)	75.3 (53.1, 106.8)	75 (52.3, 107.6)
Chinese	10	10	100000	116.7 (115, 118.5)	116 (104.3, 129)	117.7 (114.5, 121)	117.2 (105.1, 130.7)

For peer review only - http://bmjopen.bmj.com/site/about/guidelines.xhtml

FIGURE LEGENDS

Figure 1 **Scottish Health and Ethnicity Linkage Study – linkage of Health and Census datasets**

Figure 2 **Bowel cancer screening uptake by ethnicity for Round 1 relative to the White Scottish population: age-adjusted risk ratios**

Figure 3 **Bowel cancer screening uptake by religion for Round 1 relative to the reference population (Church of Scotland): age-adjusted risk ratios**

For peer review only

Figure 1 **Scottish Health and Ethnicity Linkage Study – linkage of Health and Census datasets**

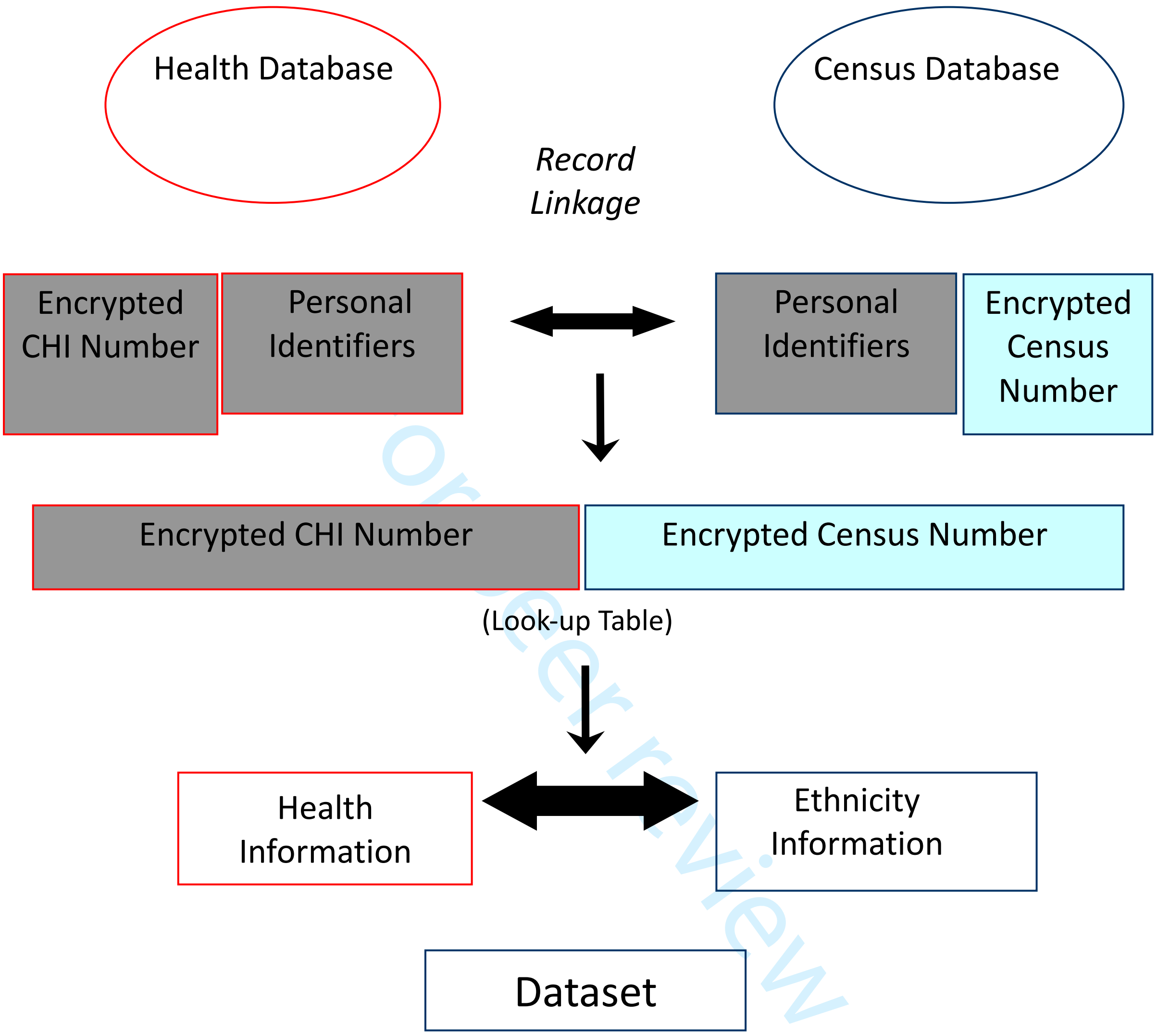

Campbell et al Ethnic and religious variations in participation in bowel cancer screening in Scotland

Figure 2 **Bowel screening uptake by ethnicity for Round 1 relative to the White Scottish population: age-adjusted risk ratios**

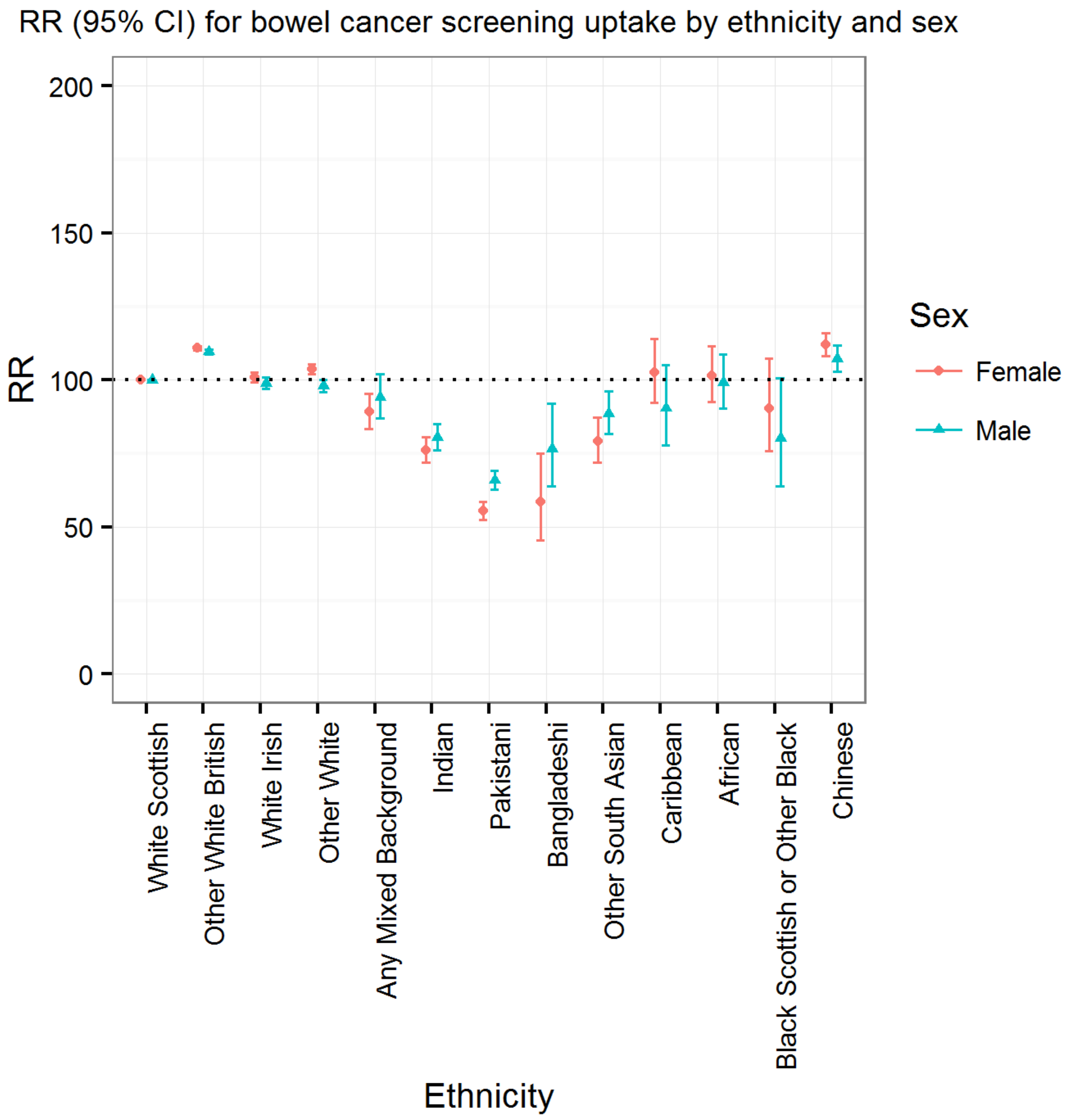

18/06/20

Campbell et al Ethnic and religious variations in participation in bowel cancer screening in Scotland

Figure 3 **Bowel cancer screening uptake by religion for Round 1 relative to the reference population (Church of Scotland): age-adjusted risk ratios**

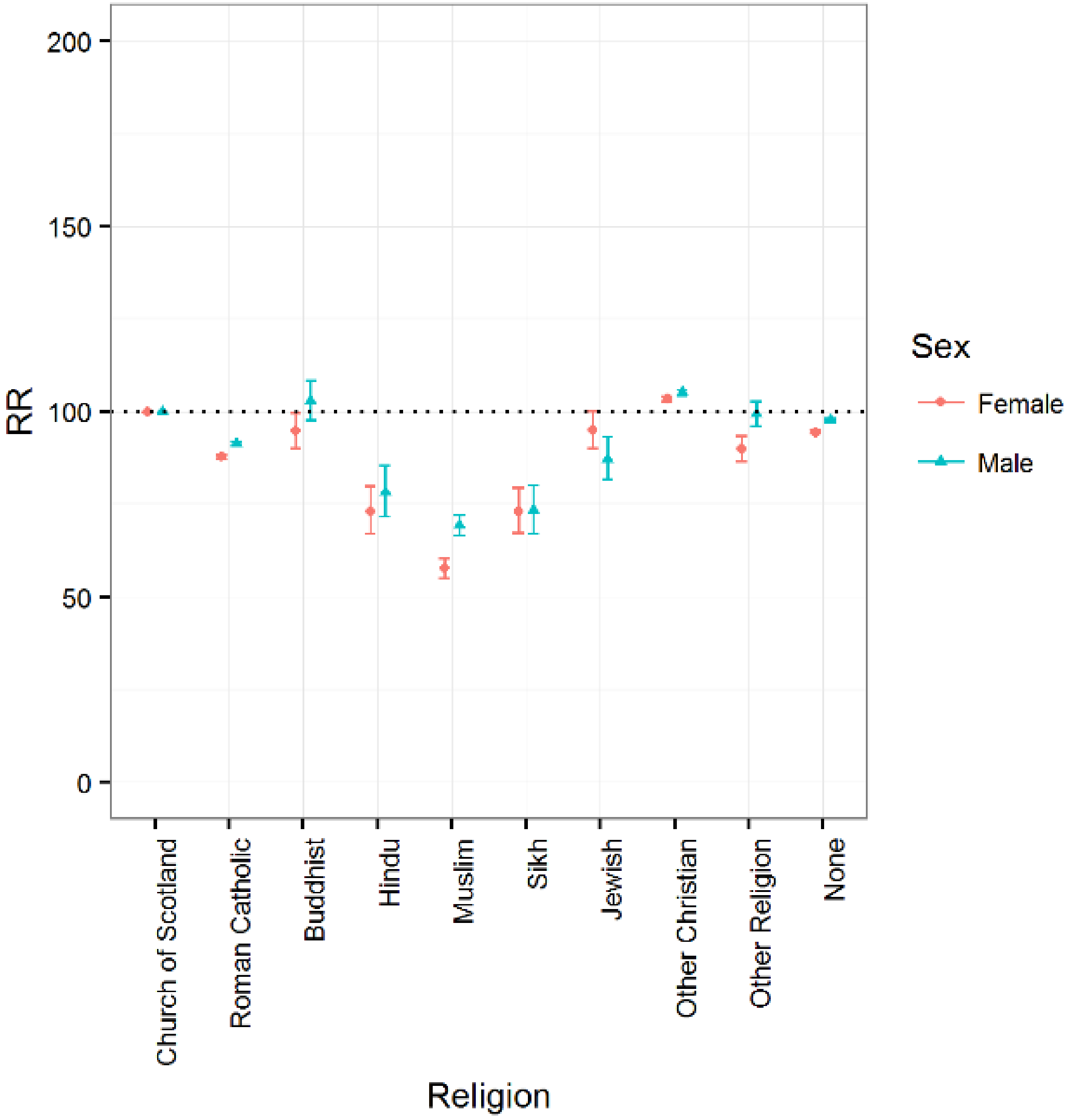

18/06/20

Supplementary Materials - Campbell et al Ethic and religious variations in bowel cancer screening in Scotland

Supplementary Material Figure 1

Directed Acrylic Graph (DAG) of factors influencing ethnicity and bowel screening uptake

A Directed Acrylic Graph (DAG) was developed, based on evidence form the literature, and discussion of study results. Initially 26 variables were identified as potential influencing factors to bowel screening uptake and a cross-matrix via expert opinion about the direction of influence between each of these variables was developed and tested for the causal effect identification in DAGitty v2.3 (http://dagitty.net/dags.html).

However, the initial model didn't allow estimation of the direct effect, and most of these variables were unobserved (latent) variables. The model was then stepwise reduced until a model was identified which allowed the estimation of the direct effecs. The figure shows the final model for the minimal sufficient adjustment set for estimating the direct effect of ethnicity on bowel screeming uptake, which included the variables age, country of birth, education, houshold tenure, religion, socioeconomic status (SES), and sex. This model informed the interpretation of our results and highlights areas for further investigation.

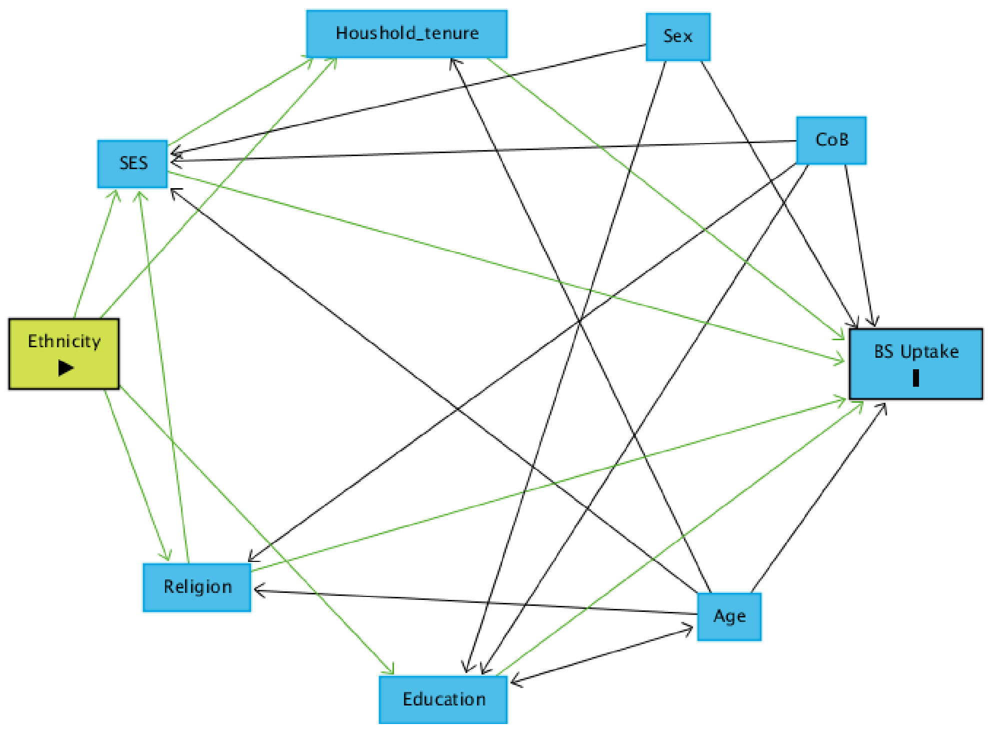

Initial factor set of 26 identified possible variables (observed variables in bold):

- **Ethnicity (Exposure)**
- **Bowel screening uptake (Outcome)**
- **Age**
- **Sex**
- Language
- Knowledge of screening
- Knowledge of cancer
- Previous experience of screening
- Health literacy
- **Education**
- Fatalism
- Perceptions of risk
- Fear of cancer
- Other priorities
- Self-efficacy
- Family history of cancer /illness
- **Country of birth (CoB)**
- **Socio-economic status**
- Marital status/ partnership
- **Religion**
- Cultural - including social norms
- Acculturation
- Frailty / co-morbidity
- **Rurality**
- Clinical support
- access to services

For peer review only

For peer review only - http://bmjopen.bmj.com/site/about/guidelines.xhtml

Supplementary Materials Tables for Campbell et al Are there ethnic and religious variations in uptake of bowel cancer screening in Scotland?

Supplementary Material Table 1A. Bowel cancer screening uptake (Round 1) by ethnic group in men: age adjusted rates and risk ratios (RR). RRs are adjusted for age, UK-born (versus born outside UK) and socio-economic status (household tenure and combined individual and household level education) with 95% confidence intervals (CIs).

				RRs (95% CIs): adjustment			
**Ethnic group**	**Completed screen kit returned**	**Invited into screening**	**Rates/ 100,000**	**Age**	**Age and UK/RoI-born**	**Age and 2 socio-economic variables**	**Age, UK/RoI-born and 2 socio-economic variables**
MEN							
White Scottish	362865	698715	51933	100	100	100	100
Other White British	37040	64855	57121	109.6 (108.8, 110.3)	109.5 (108.7, 110.2)	103 (102.2, 103.7)	102.9 (102.2, 103.7)
White Irish	4220	8155	51646	98.9 (96.9, 101)	98.9 (96.8, 101)	98.2 (96.2, 100.2)	98.2 (96.2, 100.2)
Other White	4045	8035	50320	98.1 (95.9, 100.2)	95.7 (93.4, 98.2)	94.7 (92.7, 96.8)	94.5 (92.2, 96.8)
Any Mixed Background	310	645	48094	94.1 (86.9, 102)	92.9 (85.7, 100.7)	94.5 (87.4, 102.3)	94.4 (87.2, 102.2)
Indian	705	1700	41495	80.5 (76.1, 85.1)	78 (73.6, 82.7)	75.4 (71.3, 79.7)	75.1 (70.9, 79.6)
Pakistani	1015	3040	33337	65.9 (62.7, 69.3)	63.7 (60.4, 67.2)	65.6 (62.4, 69)	65.4 (62, 68.9)
Bangladeshi	70	180	39013	76.6 (63.9, 91.9)	74.1 (61.8, 89)	77.9 (65.2, 93.1)	77.6 (64.9, 92.9)
Other South Asian	325	735	44492	88.6 (81.8, 96.1)	85.9 (79.1, 93.2)	86.4 (79.7, 93.7)	86.1 (79.4, 93.5)
Caribbean	90	200	45502	90.4 (77.7, 105.1)	88.5 (76.1, 102.9)	89.3 (76.7, 103.9)	89.1 (76.5, 103.7)
African	225	450	49560	99.1 (90.3, 108.7)	96.1 (87.5, 105.5)	99 (90.3, 108.6)	98.7 (89.9, 108.4)
Black Scottish or Other Black	45	110	40911	80.2 (64, 100.6)	79.2 (63.1, 99.4)	84 (67.2, 105)	83.9 (67.1, 104.9)
Chinese	990	1815	54579	107.2 (102.8, 111.8)	103.6 (99, 108.4)	107.4 (103, 112)	107 (102.3, 111.9)

240620

For peer review only - http://bmjopen.bmj.com/site/about/guidelines.xhtml

Supplementary Materials Tables for Campbell et al Are there ethnic and religious variations in uptake of bowel cancer screening in Scotland?

Supplementary Material Table 1B. Bowel cancer screening uptake (round 1) by ethnic group in women: age adjusted rates and risk ratios (RR). RRs are adjusted for age, UK-born (versus born outside UK) and socio-economic status (household tenure and combined individual and household level education) with 95% confidence intervals (CIs).

				RRs (95% CIs): adjustment			
**Ethnic group**	**Completed screen kit returned**	**Invited into screening**	**Rates/ 100000**	**Age**	**Age and UK/RoI-born**	**Age and 2 socio-economic variables**	**Age, UK/RoI-born and 2 socio-economic variables**
WOMEN							
White Scottish	444425	773555	57452	100	100	100	100
Other White British	42950	67160	63955	110.9 (110.2, 111.6)	110.8 (110.1, 111.4)	104.4 (103.8, 105.1)	104.4 (103.8, 105.1)
White Irish	5255	9005	58370	100.9 (99.2, 102.7)	100.9 (99.2, 102.7)	98.6 (96.9, 100.3)	98.6 (96.9, 100.3)
Other White	5840	9825	59472	103.8 (102.1, 105.5)	101 (99, 103)	97.8 (96.3, 99.4)	97.5 (95.6, 99.4)
Any Mixed Background	435	855	50564	89.2 (83.5, 95.4)	87.9 (82.3, 94)	89.2 (83.6, 95.2)	89 (83.4, 95.1)
Indian	690	1585	43409	76.1 (72, 80.5)	73.6 (69.5, 78)	72.3 (68.4, 76.4)	72 (68, 76.2)
Pakistani	870	2785	31233	55.5 (52.5, 58.6)	53.6 (50.6, 56.7)	56.7 (53.6, 59.9)	56.4 (53.3, 59.7)
Bangladeshi	40	130	32988	58.5 (45.6, 75.1)	56.7 (44.1, 72.8)	59.1 (46.2, 75.5)	58.8 (46, 75.2)
Other South Asian	225	500	45144	79.3 (72, 87.4)	77 (69.9, 84.8)	78.5 (71.5, 86.3)	78.2 (71.1, 86.1)
Caribbean	145	250	57998	102.6 (92.4, 113.9)	100.4 (90.4, 111.5)	97.3 (87.8, 107.8)	97 (87.5, 107.6)
African	190	330	57445	101.6 (92.6, 111.5)	98.4 (89.6, 108.1)	99.6 (90.8, 109.3)	99.2 (90.4, 109)
Black Scottish or Other Black	60	120	51665	90.2 (75.8, 107.2)	88.9 (74.7, 105.7)	92.7 (78.4, 109.6)	92.6 (78.3, 109.4)
Chinese	1210	1915	63190	112 (108.2, 115.9)	108 (104, 112.1)	111.7 (107.9, 115.6)	111.2 (107.1, 115.4)

240620

For peer review only

Supplementary Materials Tables for Campbell et al Are there ethnic and religious variations in uptake of bowel cancer screening in Scotland?

Supplementary Material Table 2A. Bowel cancer screening uptake (Round 2) by ethnic group in men: age adjusted rates and risk ratios (RRs). RRs are adjusted for age, UK/RoI-born (versus born outside UK/RoI) and socio-economic status (household tenure and combined individual and household level education) with 95% confidence intervals (CIs).

				RRs (95% CIs): adjustment			
**Ethnic group**	**Completed screen kit returned**	**Invited into screening**	**Rates/ 100,000**	**Age**	**Age and UK/RoI-born**	**Age and 2 socio-economic variables**	**Age, UK/RoI-born and 2 socio-economic variables**
MEN							
White Scottish	324520	590540	54952.7	100	100	100	100
Other White British	33190	55265	60056.8	108.5 (107.5, 109.6)	108.4 (107.3, 109.4)	102.4 (101.2, 103.7)	102.4 (101.2, 103.6)
White Irish	3770	6850	55073	99.7 (97.5, 101.9)	99.6 (97.5, 101.9)	99.2 (97, 101.4)	99.2 (97, 101.4)
Other White	3595	6650	54091.4	108 (91.8, 127.1)	104.3 (90.9, 119.6)	103.7 (89.5, 120.1)	102.3 (90.4, 115.8)
Any Mixed Background	265	505	52870.6	96.9 (89.2, 105.3)	95 (87.3, 103.4)	97.4 (89.7, 105.7)	96.6 (88.9, 105)
Indian	645	1440	44729	81.3 (76.8, 86.2)	77.6 (72.3, 83.3)	77.1 (72.8, 81.6)	75.7 (70.6, 81.1)
Pakistani	905	2565	35298.3	65.2 (61.7, 68.8)	62 (57.7, 66.7)	65.6 (62.1, 69.2)	64.3 (59.7, 69.2)
Bangladeshi	60	145	41666.1	76.5 (62.9, 92.9)	72.8 (59.6, 88.8)	77.1 (63.9, 93)	75.6 (62.4, 91.6)
Other South Asian	295	620	47895.8	89 (81.8, 96.8)	84.9 (77.4, 93.3)	87.1 (80.1, 94.6)	85.5 (78, 93.7)
Caribbean	75	160	46874.4	86.6 (73.5, 102.2)	83.8 (70.8, 99.1)	85.4 (72.4, 100.8)	84.3 (71.3, 99.7)
African	180	345	51872.5	96.1 (86.6, 106.6)	91.8 (82.1, 102.5)	95.7 (86.3, 106)	94 (84.2, 104.8)
Black Scottish or Other Black	45	90	46738.5	86.2 (69.3, 107.3)	84.6 (68, 105.3)	89.7 (72.3, 111.2)	89 (71.8, 110.4)
Chinese	890	1505	59162.6	108 (103.2, 113.1)	102.7 (96.3, 109.6)	109.6 (104.7, 114.8)	107.4 (100.5, 114.9)

240620

For peer review only - http://bmjopen.bmj.com/site/about/guidelines.xhtml

Supplementary Materials Tables for Campbell et al Are there ethnic and religious variations in uptake of bowel cancer screening in Scotland?

Supplementary Material Table 2B. Bowel cancer screening uptake (Round 2) by ethnic group in women: age adjusted rates and risk ratios (RRs). RRs are adjusted for age, UK/RoI-born (versus born outside UK/RoI) and socio-economic status (household tenure and combined individual and household level education) with 95% confidence intervals (CIs).

				RRs (95% CIs): adjustment			
**Ethnic group**	**Completed screen kit returned**	**Invited into screening**	**Rates/ 100.000**	**Age**	**Age and UK/RoI-born**	**Age and 2 socio-economic variables**	**Age, UK/RoI-born and 2 socio-economic variables**
WOMEN							
White Scottish	388730	654990	59348.8	100	100	100	100
Other White British	37600	57050	66369.9	110.7 (110, 111.4)	110.4 (109.8, 111.1)	104.5 (103.9, 105.1)	104.4 (103.8, 105.1)
White Irish	4610	7625	60891.6	101 (99.2, 102.9)	101 (99.2, 102.9)	98.9 (97.1, 100.7)	98.9 (97.1, 100.7)
Other White	5090	8215	107045.9	104.7 (102.9, 106.5)	100.2 (98.2, 102.3)	98.8 (97.2, 100.5)	97 (95, 98.9)
Any Mixed Background	390	710	55407.6	94.1 (88, 100.5)	91.8 (85.8, 98.1)	94.3 (88.3, 100.6)	93.2 (87.3, 99.5)
Indian	620	1355	45997	77.6 (73.3, 82.2)	73.5 (69.3, 78)	73.8 (69.6, 78.1)	72 (67.9, 76.4)
Pakistani	795	2330	34324.4	58.9 (55.6, 62.3)	55.6 (52.5, 59)	59.9 (56.7, 63.4)	58.5 (55.2, 62)
Bangladeshi	40	110	35432.3	60.7 (46.8, 78.7)	57.6 (44.4, 74.7)	60.4 (46.9, 77.8)	59 (45.8, 76.1)
Other South Asian	200	405	49852.6	84.2 (76.4, 92.9)	80.2 (72.7, 88.6)	83.1 (75.3, 91.6)	81.3 (73.6, 89.7)
Caribbean	120	210	57819.5	98.2 (87.5, 110.2)	94.6 (84.2, 106.1)	93.2 (83.1, 104.6)	91.7 (81.7, 102.8)
African	165	270	62375.3	105.8 (96.3, 116.2)	100.4 (91.3, 110.4)	103.1 (93.7, 113.5)	100.8 (91.5, 111.1)
Black Scottish or Other Black	45	95	49809.7	83.8 (68.6, 102.4)	81.7 (66.9, 99.8)	84.9 (70.1, 102.8)	83.9 (69.2, 101.6)
Chinese	1080	1605	67867.3	115.7 (111.8, 119.8)	109.1 (105.1, 113.3)	115.2 (111.3, 119.2)	112.2 (108.1, 116.5)

240620

For peer review only

Supplementary Materials Tables for Campbell et al Are there ethnic and religious variations in uptake of bowel cancer screening in Scotland?

Supplementary Material Table 3A. Bowel cancer screening uptake (Round 1) by country of birth in men: age adjusted rates and risk ratios (RRs). RRs are adjusted for age and socio-economic status (household tenure and combined individual and household level education) with 95% confidence intervals (CIs).

				RRs (95% CIs): adjustment	
**Country of birth**	**Completed screen kit returned**	**Invited into screening**	**Rates/ 100,000**	**Age**	**Age and 2 socio-economic variables**
MEN					
Scotland	355310	685975	51796.4	100	100
Northern Ireland	2720	4890	55376.5	107 (104.4, 109.7)	102.9 (100.4, 105.4)
Rep Ireland	1695	3240	52218.9	97.8 (94.6, 101.1)	99.3 (96.2, 102.6)
Other UK	42015	73785	56952.8	109.6 (108.9, 110.3)	103.2 (102.5, 103.9)
India	1070	2240	47726.8	90.1 (86.3, 94)	83.8 (80.3, 87.4)
Pakistan	855	2670	32067.9	63.8 (60.4, 67.4)	63.5 (60.1, 67.1)
Bangladesh	80	195	40936.1	81 (68.4, 96.1)	80.9 (68.4, 95.6)
China	185	345	53606.9	102.3 (92.8, 112.8)	100.1 (90.9, 110.2)
Hong Kong	815	1470	55631.6	110 (105.1, 115.2)	110 (105.1, 115.1)
Africa	1715	3330	51506.8	102.1 (98.8, 105.5)	96.2 (93.1, 99.4)
Caribbean and West Indies	130	245	52873.4	102.8 (91.5, 115.5)	96.2 (85.6, 108)
Rest of the World	5740	11135	51544.3	101.6 (99.8, 103.5)	97.7 (96, 99.5)

240620

For peer review only - http://bmjopen.bmj.com/site/about/guidelines.xhtml

Supplementary Materials Tables for Campbell et al Are there ethnic and religious variations in uptake of bowel cancer screening in Scotland?

Supplementary Material Table 3B. Bowel cancer screening uptake (Round 1) by country of birth in women: age adjusted rates and risk ratios (RRs). RRs are adjusted for age and socio-economic status (household tenure and combined individual and household level education) with 95% confidence intervals (CIs).

Country of birth	Completed screen kit returned	Invited into screening	Rates/ 100,000	RRs (95% CIs): adjustment Age	Age and 2 socio-economic variables
WOMEN					
Scotland	435740	760005	57333.7	100	100
Northern Ireland	3410	5490	62120	108.1 (105.9, 110.4)	103.4 (101.4, 105.6)
Rep Ireland	2615	4365	59889.6	102.8 (100.3, 105.3)	101.7 (99.3, 104.1)
Other UK	47935	75540	63453.7	110.4 (109.7, 111)	104.3 (103.7, 104.9)
India	1065	2110	50385.7	86.9 (83.3, 90.6)	81.1 (77.8, 84.6)
Pakistan	780	2455	31807.7	56.6 (53.4, 60)	57.8 (54.6, 61.2)
Bangladesh	45	120	38227.6	67.8 (53.9, 85.4)	66.9 (53.3, 83.9)
China	275	435	62469.5	110.7 (103, 119.1)	108.5 (101, 116.7)
Hong Kong	850	1350	62987.5	112.6 (108, 117.3)	112.9 (108.4, 117.7)
Africa	1860	3110	59869.6	106 (103, 109.1)	99.5 (96.7, 102.4)
Caribbean and West Indies	220	335	66064.1	115.8 (107.3, 125.1)	105.7 (97.9, 114.1)
Rest of the World	8115	13755	59017.1	104.1 (102.7, 105.6)	98.9 (97.5, 100.2)

240620

Supplementary Materials Tables for Campbell et al Are there ethnic and religious variations in uptake of bowel cancer screening in Scotland?

Supplementary Material Table 4A. Bowel cancer screening uptake (Round 1) by religion of upbringing in men: age adjusted rates and risk ratios (RRs). RRs are adjusted for age, UK/RoI-born (versus born outside UK/RoI) and socio-economic status (household tenure and combined individual and household level education) with 95% confidence intervals (CIs).

				RRs (95% CIs): adjustment			
**Religion of upbringing**	**Completed screen kit returned**	**Invited into screening**	**Rates/ 100,000**	**Age**	**Age and UK/RoI-born**	**Age and 2 socio-economic variables**	**Age, UK/RoI-born and 2 socio-economic variables**
MEN							
Church of Scotland	243955	450020	54209.6	100	100	100	100
Roman Catholic	66100	134685	48802.5	91.9 (91.3, 92.4)	91.8 (91.2, 92.3)	94.6 (94, 95.1)	94.6 (94, 95.1)
Buddhist	250	495	50604.3	94.8 (86.9, 103.4)	91.9 (84.2, 100.3)	96.2 (88.2, 105)	96.1 (88, 105)
Hindu	325	730	44185.5	81.2 (74.9, 88)	78.5 (72.3, 85.2)	74.3 (68.5, 80.5)	74.2 (68.3, 80.6)
Muslim	1615	4410	36626.2	70 (67.4, 72.8)	67.7 (64.9, 70.5)	70.4 (67.7, 73.2)	70.4 (67.5, 73.3)
Sikh	310	800	38923.2	73.6 (67.5, 80.3)	71.5 (65.6, 78.1)	72.2 (66.3, 78.7)	72.2 (66.2, 78.7)
Jewish	585	1240	46939.9	86.3 (81.4, 91.6)	86.1 (81.1, 91.3)	81.6 (77, 86.6)	81.6 (77, 86.6)
Other Christian	42550	72280	58868.5	108.2 (107.5, 108.9)	108 (107.3, 108.7)	103 (102.3, 103.7)	103 (102.3, 103.7)
Other Religion	370	825	44848	85.2 (79, 91.9)	84.8 (78.6, 91.4)	84.3 (78.3, 90.9)	84.3 (78.3, 90.9)
None	38330	83260	46036.6	88.1 (87.4, 88.8)	88 (87.3, 88.7)	91.5 (90.8, 92.2)	91.5 (90.8, 92.2)

240620

For peer review only - http://bmjopen.bmj.com/site/about/guidelines.xhtml

Supplementary Materials Tables for Campbell et al Are there ethnic and religious variations in uptake of bowel cancer screening in Scotland?

Supplementary Material Table 4B. Bowel cancer screening uptake (Round 1) by religion of upbringing in women: age adjusted rates and risk ratios (RRs). RRs are adjusted for age, UK/RoI-born (versus born outside UK/RoI) and socio-economic status (household tenure and combined individual and household level education) with 95% confidence intervals (CIs).

				RRs (95% CIs): adjustment			
**Religion of upbringing**	**Completed screen kit returned**	**Invited into screening**	**Rates/ 100,000**	**Age**	**Age and UK/RoI-born**	**Age and 2 socio-economic variables**	**Age, UK/RoI-born and 2 socio-economic variables**
WOMEN							
Church of Scotland	300940	499325	60269	100	100	100	100
Roman Catholic	83420	156940	53154	88.9 (88.4, 89.4)	88.8 (88.3, 89.2)	91.5 (91.1, 92)	91.5 (91, 92)
Buddhist	400	710	56336.8	95.4 (89.4, 101.8)	90.1 (84.4, 96.2)	96.5 (90.5, 102.9)	95 (89, 101.4)
Hindu	280	645	43584.8	72.4 (66.3, 79)	68.3 (62.5, 74.6)	67.1 (61.5, 73.3)	66.1 (60.5, 72.2)
Muslim	1090	3265	33363.3	56.5 (53.8, 59.3)	53.3 (50.7, 56.1)	58.4 (55.6, 61.2)	57.4 (54.7, 60.4)
Sikh	340	790	43061.2	72.3 (66.7, 78.3)	68.9 (63.6, 74.7)	72.7 (67.2, 78.8)	71.8 (66.2, 77.8)
Jewish	705	1235	57014.2	93.7 (89.2, 98.3)	92.9 (88.5, 97.5)	88.4 (84.3, 92.8)	88.2 (84.1, 92.6)
Other Christian	55265	85070	64964.8	107.4 (106.9, 108)	107.1 (106.5, 107.7)	102.9 (102.4, 103.5)	102.8 (102.3, 103.4)
Other Religion	365	700	51924.7	87.2 (81.2, 93.6)	86.3 (80.4, 92.6)	88.6 (82.6, 95)	88.3 (82.4, 94.7)
None	34110	68420	49855.9	85 (84.3, 85.6)	84.8 (84.1, 85.5)	90.3 (89.6, 91)	90.2 (89.5, 90.9)

240620

For peer review only

Supplementary Materials Tables for Campbell et al Are there ethnic and religious variations in uptake of bowel cancer screening in Scotland?

Supplementary Material Table 5A. Positive screen test results (Round 2) by ethnic group in men: age adjusted rates and risk ratios (RRs). RRs are adjusted for age, UK/RoI-born (versus born outside UK/RoI) and socio-economic status (household tenure and combined individual and household level education) with 95% confidence intervals (CIs).

				RRs (95% CIs): adjustment			
**Ethnic group**	**Positive screen test results**	**Completed screen kit returned**	**Rates/ 100,000**	**Age**	**Age and UK/RoI-born**	**Age and 2 socio-economic variables**	**Age, UK/RoI-born and 2 socio-economic variables**
MEN							
White Scottish	10540	324520	3248.2	100	100	100	100
Other White British	705	33190	2124	65.3 (60.5, 70.4)	65.5 (60.8, 70.6)	74.3 (68.8, 80.2)	74.4 (68.9, 80.3)
White Irish	145	3770	3870.6	117.2 (100.1, 137.3)	117.3 (100.1, 137.3)	118 (100.8, 138.2)	118 (100.8, 138.2)
Other White	105	3595	2892.1	91.8 (75.9, 110.9)	101.2 (80.6, 126.9)	100 (82.7, 120.9)	104.7 (82.9, 132.1)
Any Mixed Background	10	265	4494.3	144.4 (83, 251.3)	152.4 (87, 267.1)	146.8 (84, 256.6)	150.3 (85.4, 264.6)
Indian	20	645	2790.7	86.3 (54.6, 136.4)	98.5 (60.5, 160.3)	100.1 (63.5, 157.9)	106.3 (65.4, 172.8)
Pakistani	25	905	2761.6	85.1 (57.6, 125.5)	97.8 (63.6, 150.4)	84.1 (57, 124.1)	89.6 (58.2, 138.2)
Other South Asian	10	355	3370.8	120 (68.8, 209.2)	137 (76.8, 244.3)	134.5 (77, 234.8)	142.7 (80, 254.6)
African origin	10	300	3020.1	107.3 (56.3, 204.2)	119.9 (62.1, 231.2)	113.9 (59.8, 216.9)	119.8 (62.1, 231)
Chinese	30	890	3254.7	111.8 (78.6, 159.1)	128.7 (87, 190.3)	107.6 (75.8, 152.8)	114.8 (77.6, 169.8)

240620

For peer review only - http://bmjopen.bmj.com/site/about/guidelines.xhtml

Supplementary Materials Tables for Campbell et al Are there ethnic and religious variations in uptake of bowel cancer screening in Scotland?

Supplementary Material Table 5B. Positive screen test results (Round 2) by ethnic group in women: age adjusted rates and risk ratios (RRs). RRs are adjusted for age, UK/RoI-born (versus born outside UK/RoI) and socio-economic status (household tenure and combined individual and household level education) with 95% confidence intervals (CIs).

				RRs (95% CIs): adjustment			
**Ethnic group**	**Positive screen test results**	**Completed screen kit returned**	**Rates/ 100,000**	**Age**	**Age and UK/RoI-born**	**Age and 2 socio-economic variables**	**Age, UK/RoI-born and 2 socio-economic variables**
WOMEN							
White Scottish	8050	388730	2070.9	100	100	100	100
Other White British	490	37600	1300.5	63.8 (58.3, 69.8)	64.2 (58.6, 70.2)	71.6 (65.4, 78.4)	71.8 (65.6, 78.7)
White Irish	90	4610	1973.9	91.8 (75, 112.3)	91.8 (75, 112.4)	93.9 (76.7, 114.9)	93.9 (76.7, 114.9)
Other White	70	5090	1375.5	68.3 (54.2, 86.2)	78.7 (60, 103.3)	76.8 (60.9, 96.9)	84 (63.7, 110.8)
Any Mixed Background	10	390	2561.7	132.2 (72.5, 241)	143.8 (77.9, 265.3)	134.2 (73.2, 246.1)	140.9 (76.1, 261.1)
Indian	10	620	1291.6	64.5 (32.2, 129)	77.1 (37.3, 159.3)	69.9 (35, 139.6)	78.1 (37.8, 161.3)
Pakistani	20	795	2644.8	141.7 (92.6, 216.9)	169.7 (107.5, 268)	132.7 (86.8, 202.8)	148.6 (93.8, 235.4)
Other South Asian	.	240					
African origin	10	330	2108.4	109.7 (52.6, 228.8)	127 (60.4, 267.4)	119.1 (57.3, 247.5)	130.4 (62.1, 273.6)
Chinese	20	1080	2032.6	112.6 (74.3, 170.7)	136.5 (85.8, 217.2)	108.5 (71.5, 164.7)	122.5 (76.7, 195.8)

240620

Supplementary Materials Tables for Campbell et al Are there ethnic and religious variations in uptake of bowel cancer screening in Scotland?

Supplementary Material Table 6A. Colonoscopy performed (Round 1) by ethnic group in men: age adjusted rates and risk ratios (RRs). Results exclude Grampian health board. RRs are adjusted for age, UK/RoI-born (versus born outside UK/RoI) and socio-economic status (household tenure and combined individual and household level education) with 95% confidence intervals (CIs).

				RRs (95% CIs): adjustment			
**Ethnic group**	**Colonoscopy performed**	**Positive screen test results**	**Rates/ 100,000**	**Age**	**Age and UK/RoI-born**	**Age and 2 socio-economic variables**	**Age, UK/RoI-born and 2 socio-economic variables**
MEN							
White Scottish	8380	10125	82765.4	100	100	100	100
Other White British	495	595	83172.7	100.6 (98.1, 103.1)	100.5 (98, 103)	100 (97.5, 102.5)	99.9 (97.4, 102.4)
White Irish	100	125	78144.9	94.5 (88, 101.5)	94.6 (88.1, 101.5)	94.7 (88.2, 101.7)	94.7 (88.2, 101.7)
Other White	80	95	83447.6	100.8 (95, 107)	97.6 (91.4, 104.2)	100.5 (94.7, 106.7)	97.5 (91.4, 104.1)
Any Mixed Background	.	10					
Indian	10	10	89866.7	108.9 (107.9, 109.8)	103.6 (99.7, 107.7)	108 (106.8, 109.1)	103.2 (99.2, 107.4)
Pakistani	15	20	76386.7	92.2 (76.7, 111)	88 (73, 106.2)	92 (76.3, 110.9)	88.2 (72.9, 106.5)
Other South Asian	10	10	79881.5	96.5 (76.4, 121.8)	92.8 (73.4, 117.4)	96.8 (76.3, 122.7)	93.4 (73.5, 118.7)
African origin	10	10	77028.6	93.1 (68.8, 125.9)	90.5 (67, 122.2)	93.1 (68.4, 126.6)	90.7 (66.8, 123.2)
Chinese	20	30	81307.9	98.3 (85.6, 112.9)	93.8 (81.4, 108.2)	98.1 (85.6, 112.5)	94 (81.6, 108.2)

240620

For peer review only

Supplementary Materials Tables for Campbell et al Are there ethnic and religious variations in uptake of bowel cancer screening in Scotland?

Supplementary Material Table 6B. Colonoscopy performed (Round 1) by ethnic group in women: age adjusted rates and risk ratios (RRs). Results exclude Grampian health board. RRs are adjusted for age, UK/RoI-born (versus born outside UK/RoI) and socio-economic status (household tenure and combined individual and household level education) with 95% confidence intervals (CIs).

				RRs (95% CIs): adjustment			
**Ethnic group**	**Colonoscopy performed**	**Positive screen test results**	**Rates/ 100,000**	**Age**	**Age and UK/RoI-born**	**Age and 2 socio-economic variables**	**Age, UK/RoI-born and 2 socio-economic variables**
WOMEN							
White Scottish	5870	7295	80466.1	100	100	100	100
Other White British	365	450	78820.6	98 (94.6, 101.4)	97.7 (94.3, 101.2)	97.4 (94.1, 100.9)	97.2 (93.9, 100.7)
White Irish	60	80	81980.5	102 (95.2, 109.2)	101.7 (95, 108.9)	102.5 (95.7, 109.7)	102.2 (95.5, 109.5)
Other White	60	85	75242.4	93.3 (84.7, 102.8)	89.7 (80.2, 100.4)	92.6 (84.1, 102)	89.3 (79.8, 99.9)
Any Mixed Background	10	10	77517.8	97.1 (74.6, 126.2)	95.6 (73.7, 124.1)	99 (76.3, 128.5)	97.6 (75.4, 126.4)
Indian	10	10	70873.4	87.9 (64.6, 119.6)	82.7 (60.3, 113.3)	86.6 (64.1, 116.9)	81.7 (60, 111.2)
Pakistani	10	15	88591.8	109.8 (108.2, 111.4)	103.3 (96.3, 110.8)	109.7 (107.3, 112.2)	103.5 (96.3, 111.3)
Other South Asian	.	.					
African origin	.	10					
Chinese	15	25	78820.6	93.3 (77.5, 112.3)	87.7 (72, 106.9)	92.3 (76.6, 111.2)	87.1 (71.4, 106.2)

240620

Supplementary Materials Tables for Campbell et al Are there ethnic and religious variations in uptake of bowel cancer screening in Scotland?

Supplementary Material Table 6C. Colonoscopy performed (Round 2) by ethnic group in men: age adjusted rates and risk ratios (RRs). Results exclude Grampian health board. RRs are adjusted for age, UK/RoI-born (versus born outside UK/RoI) and socio-economic status (household tenure and combined individual and household level education) with 95% confidence intervals (CIs).

				RRs (95% CIs): adjustment			
**Ethnic group**	**Colonoscopy performed**	**Positive screen test results**	**Rates/ 100,000**	**Age**	**Age and UK/RoI-born**	**Age and 2 socio-economic variables**	**Age, UK/RoI-born and 2 socio-economic variables**
MEN							
White Scottish	7935	9505	83482.4	100	100	100	100
Other White British	505	595	82759.7	99.3 (96.7, 101.9)	99.3 (96.7, 101.9)	98.8 (96.2, 101.5)	98.8 (96.2, 101.5)
White Irish	115	140	84825.9	101.7 (97.1, 106.5)	101.7 (97.1, 106.5)	102 (97.4, 106.9)	102 (97.4, 106.9)
Other White	85	95	84584.3	100.9 (95.5, 106.7)	101.3 (95.2, 107.7)	100.7 (95.1, 106.5)	101.4 (95.2, 108)
Any Mixed Background	10	10	90626	108.4 (107.4, 109.5)	108.6 (107.1, 110.1)	108.8 (107.2, 110.5)	109.1 (107.1, 111.1)
Indian	15	15	84961.9	101.3 (89.3, 115)	101.8 (89.3, 116.1)	101.1 (89.6, 114.1)	102.2 (90, 116)
Pakistani	20	25	78805.2	93.8 (80, 110.1)	94.3 (80.1, 110.9)	93.3 (79.5, 109.6)	94.3 (80.1, 111)
Other South Asian	10	10	90626	108.1 (107, 109.1)	108.6 (103.5, 113.9)	107.9 (105.9, 109.9)	109.1 (103.7, 114.6)
African origin	10	10	90626	107.4 (106.4, 108.5)	107.8 (103.7, 112.2)	107.8 (105.5, 110.2)	108.8 (104.1, 113.6)
Chinese	25	25	90626	108.3 (107.5, 109.2)	108.9 (103.6, 114.5)	108.6 (107.3, 109.9)	109.9 (104.4, 115.6)

240620

For peer review only

Supplementary Materials Tables for Campbell et al Are there ethnic and religious variations in uptake of bowel cancer screening in Scotland?

Supplementary Material Table 6D. Colonoscopy performed (Round 2) by ethnic group in women: age adjusted rates and risk ratios (RRs). Results exclude Grampian health board. RRs are adjusted for age, UK/RoI-born (versus born outside UK/RoI) and socio-economic status (household tenure and combined individual and household level education) with 95% confidence intervals (CIs).

				RRs (95% CIs): adjustment			
**Ethnic group**	**Colonoscopy performed**	**Positive screen test results**	**Rates/ 100,000**	**Age**	**Age and UK/RoI-born**	**Age and 2 socio-economic variables**	**Age, UK/RoI-born and 2 socio-economic variables**
WOMEN							
White Scottish	6080	7355	82664.9	100	100	100	100
Other White British	365	435	82495	99.8 (96.8, 102.9)	99.9 (96.9, 103)	99.4 (96.4, 102.5)	99.5 (96.5, 102.6)
White Irish	70	85	79995.2	96.9 (89.7, 104.8)	96.9 (89.6, 104.8)	96.8 (89.5, 104.7)	96.8 (89.5, 104.7)
Other White	55	65	84093.3	101.8 (95.2, 108.8)	104 (94.9, 114)	101.9 (95.3, 109)	104.3 (95.2, 114.1)
Any Mixed Background	10	10	89994.6	109.1 (107.8, 110.3)	110.1 (106.7, 113.5)	109.2 (107.9, 110.6)	110.3 (106.9, 113.8)
Indian	10	10	89994.6	107.6 (106.4, 108.8)	110.1 (102.7, 118.2)	107.1 (105.4, 108.8)	109.7 (102.2, 117.8)
Pakistani	15	20	64996.1	78.1 (58.6, 104.1)	80.6 (59.6, 108.9)	78.1 (58.7, 103.9)	80.6 (59.7, 108.9)
Other South Asian	.	.					
African origin	.	10					
Chinese	20	20	85258	102.6 (92.2, 114.1)	105.6 (92.1, 121.1)	101.8 (91.5, 113.3)	105 (91.6, 120.4)

240620

For peer review only

Supplementary Materials Tables for Campbell et al Are there ethnic and religious variations in uptake of bowel cancer screening in Scotland?

Supplementary Material Table 7A. Pathology detected (polyps, adenomas, cancers) (Rounds 1 & 2) by ethnic group in men: age adjusted rates and risk ratios (RRs). Results exclude Grampian health board. RRs are adjusted for age, UK/RoI-born (versus born outside UK/RoI) and socio-economic status (household tenure and combined individual and household level education) with 95% confidence intervals (CIs).

				RRs (95% CIs): adjustment			
**Ethnic group**	**Pathology detected**	**Positive screen test results**	**Rates/ 100,000**	**Age**	**Age and UK/RoI-born**	**Age and 2 socio-economic variables**	**Age, UK/RoI-born and 2 socio-economic variables**
MEN							
White Scottish	10140	19630	51655.6	100	100	100	100
Other White British	615	1190	51922	100 (94.4, 105.8)	99.9 (94.4, 105.8)	97 (91.6, 102.7)	97 (91.5, 102.7)
White Irish	140	265	52073.3	100 (89, 112.4)	100 (89.1, 112.4)	100.9 (89.9, 113.3)	100.9 (89.9, 113.3)
Other White	95	190	50291.3	98.3 (85.3, 113.3)	96.8 (82, 114.3)	96.4 (83.7, 111.1)	96.2 (81.6, 113.4)
Any Mixed Background	10	15	46756.9	90.3 (56.4, 144.6)	89.7 (55.8, 144.1)	91 (57, 145.1)	90.9 (56.9, 145.2)
Indian	15	30	48832.3	95.6 (65.8, 139.1)	93.4 (63, 138.7)	93.3 (64.2, 135.5)	93 (62.7, 138)
Pakistani	15	45	32456.4	64.5 (42.5, 97.7)	63 (40.8, 97.4)	64.2 (42.5, 97)	64.1 (41.6, 98.6)
Other South Asian	10	20	36533	73.1 (40.7, 131.3)	71.6 (39.3, 130.4)	71.2 (39.5, 128.1)	71 (38.9, 129.6)
African origin	10	15	49766.5	99.1 (60.7, 162.1)	97.4 (59, 160.9)	98.4 (60.4, 160.4)	98.2 (59.7, 161.6)
Chinese	25	55	43452	84.5 (62.5, 114.3)	82.5 (59.5, 114.4)	85.5 (63.2, 115.8)	85.3 (61.4, 118.5)

240620

Supplementary Materials Tables for Campbell et al Are there ethnic and religious variations in uptake of bowel cancer screening in Scotland?

Supplementary Material Table 7B. Pathology detected (polyps, adenomas, cancers) (Rounds 1 & 2) by ethnic group in women: age adjusted rates and risk ratios (RRs). Results exclude Grampian health board. RRs are adjusted for age, UK/RoI-born (versus born outside UK/RoI) and socio-economic status (household tenure and combined individual and household level education) with 95% confidence intervals (CIs).

Ethnic group	Pathology detected	Positive screen test results	Rates	Age/ 100,000	RRs (95% CIs): adjustment<br>Age and UK/RoI-born	Age and 2 socio-economic variables	Age, UK/RoI-born and 2 socio-economic variables
WOMEN							
White Scottish	5275	14650	36006.8	100	100	100	100
Other White British	340	880	38451.2	107 (98.2, 116.7)	107 (98.1, 116.7)	103.4 (94.8, 112.8)	103.4 (94.8, 112.9)
White Irish	70	165	40946.5	111.9 (93, 134.5)	111.8 (93, 134.5)	111.9 (93.2, 134.4)	111.9 (93.2, 134.4)
Other White	50	150	32755.6	92.2 (73.3, 115.8)	91.5 (69.9, 119.7)	89.9 (71.6, 112.9)	90.2 (69.1, 117.9)
Any Mixed Background	10	20	30072.4	85 (44, 164.2)	84.7 (43.8, 163.9)	86.4 (44, 169.9)	86.6 (44, 170.4)
Indian	10	20	41942.6	126.4 (74.2, 215.1)	125.1 (71.4, 219.2)	124 (73, 210.3)	124.6 (71.3, 217.7)
Pakistani	.	35					
Other South Asian	.	.					
African origin	.	10					
Chinese	15	45	28605.7	83.9 (52.9, 132.9)	82.9 (50.1, 137.4)	82.3 (52, 130.5)	82.8 (50.1, 137)

240620

For peer review only - http://bmjopen.bmj.com/site/about/guidelines.xhtml

STROBE Statement—Checklist of items that should be included in reports of ***cohort studies***

	Item No	Recommendation	Page No
**Title and abstract**	1	(*a*) Indicate the study's design with a commonly used term in the title or the abstract	1
		(*b*) Provide in the abstract an informative and balanced summary of what was done and what was found	3
**Introduction**			
Background/rationale	2	Explain the scientific background and rationale for the investigation being reported	5
Objectives	3	State specific objectives, including any prespecified hypotheses	6
**Methods**			
Study design	4	Present key elements of study design early in the paper	7
Setting	5	Describe the setting, locations, and relevant dates, including periods of recruitment, exposure, follow-up, and data collection	7-9
Participants	6	(*a*) Give the eligibility criteria, and the sources and methods of selection of participants. Describe methods of follow-up	7-8
		(*b*) For matched studies, give matching criteria and number of exposed and unexposed	
Variables	7	Clearly define all outcomes, exposures, predictors, potential confounders, and effect modifiers. Give diagnostic criteria, if applicable	8
Data sources/ measurement	8*	For each variable of interest, give sources of data and details of methods of assessment (measurement). Describe comparability of assessment methods if there is more than one group	7/8
Bias	9	Describe any efforts to address potential sources of bias	7
Study size	10	Explain how the study size was arrived at	7
Quantitative variables	11	Explain how quantitative variables were handled in the analyses. If applicable, describe which groupings were chosen and why	8
Statistical methods	12	(*a*) Describe all statistical methods, including those used to control for confounding	8,9
		(*b*) Describe any methods used to examine subgroups and interactions	
		(*c*) Explain how missing data were addressed	
		(*d*) If applicable, explain how loss to follow-up was addressed	
		(*e*) Describe any sensitivity analyses	
**Results**			
Participants	13*	(a) Report numbers of individuals at each stage of study—eg numbers potentially eligible, examined for eligibility, confirmed eligible, included in the study, completing follow-up, and analysed	10
		(b) Give reasons for non-participation at each stage	
		(c) Consider use of a flow diagram	
Descriptive data	14*	(a) Give characteristics of study participants (eg demographic, clinical, social) and information on exposures and potential confounders	10
		(b) Indicate number of participants with missing data for each variable of interest	
		(c) Summarise follow-up time (eg, average and total amount)	
Outcome data	15*	Report numbers of outcome events or summary measures over time	10

For peer review only

For peer review only - http://bmjopen.bmj.com/site/about/guidelines.xhtml

Main results	16	(*a*) Give unadjusted estimates and, if applicable, confounder-adjusted estimates and their precision (eg, 95% confidence interval). Make clear which confounders were adjusted for and why they were included<br>(*b*) Report category boundaries when continuous variables were categorized<br>(*c*) If relevant, consider translating estimates of relative risk into absolute risk for a meaningful time period	10-16
Other analyses	17	Report other analyses done—eg analyses of subgroups and interactions, and sensitivity analyses	8
**Discussion**			
Key results	18	Summarise key results with reference to study objectives	19
Limitations	19	Discuss limitations of the study, taking into account sources of potential bias or imprecision. Discuss both direction and magnitude of any potential bias	19-20
Interpretation	20	Give a cautious overall interpretation of results considering objectives, limitations, multiplicity of analyses, results from similar studies, and other relevant evidence	20-21
Generalisability	21	Discuss the generalisability (external validity) of the study results	21
**Other information**			
Funding	22	Give the source of funding and the role of the funders for the present study and, if applicable, for the original study on which the present article is based	22

*Give information separately for exposed and unexposed groups.

Note: An Explanation and Elaboration article discusses each checklist item and gives methodological background and published examples of transparent reporting. The STROBE checklist is best used in conjunction with this article (freely available on the Web sites of PLoS Medicine at http://www.plosmedicine.org/, Annals of Internal Medicine at http://www.annals.org/, and Epidemiology at http://www.epidem.com/). Information on the STROBE Initiative is available at http://www.strobe-statement.org.

For peer review only
